# Analysis of Intestinal Microbiota and Metabolic Pathways before and after a 2-Month-Long Hydrolyzed Fish and Rice Starch Hypoallergenic Diet Trial in Pruritic Dogs

**DOI:** 10.3390/vetsci10070478

**Published:** 2023-07-21

**Authors:** Chiara Noli, Antonella Varina, Caterina Barbieri, Alessandra Pirola, Daniela Olivero

**Affiliations:** 1 Servizi Dermatologici Veterinari, Strada Bedale della Ressia 2, 12016 Peveragno, Italy; 2 Ambulatorio Veterinario Varina-Ghidella-Scarfone, Via Fréjus 54, 10139 Torino, Italy; anto.varina@gmail.com; 3 GalSeq s.r.l., Via Ludovico Ariosto 21, 20091 Bresso, Italy; caterina.barbieri@galseq.com (C.B.); bioinformatics@galseq.com (A.P.); 4 Laboratorio Analisi Veterinarie BiEsseA Scilvet, Via Amedeo d’Aosta 7, 20129 Milano, Italy; daniela.olivero@scilvet.com

**Keywords:** dog, allergic dermatitis, food allergy, pruritus, skin, canine atopic dermatitis, intestinal microbiota, metabolic pathways, hydrolyzed protein, rice starch

## Abstract

**Simple Summary:**

Intestinal microbiota alterations were described in allergic people, which may improve with special diets, leading to decrease in allergy symptoms. Farmina Ultra Hypo (FUH) is a hypoallergenic diet able to improve pruritus and skin lesions in allergic dogs. Study objectives were to determine intestinal microbiota alterations in skin allergic dogs and the effect of feeding FUH. Forty skin allergic dogs were fed FUH for 8 weeks and feces were collected before and after the trial. Dogs clinically improving with the diet were considered food allergic, while those not improving were considered allergic to the environment (affected by atopic dermatitis). As previously reported, the gut microbiota in all dogs was dominated by *Bacteroidota*, *Fusobacteriota*, *Firmicutes* and *Proteobacteria*, albeit with large variations between dogs and with some changes after the diet. In general, bacteria producing beneficial short-chain fatty acids were increased in all samples. Dogs affected with atopic dermatitis showed different pre-and post-diet microbiota patterns to food allergic dogs. The number of bacterial types was increased after the diet only in food allergic dogs. Changes in metabolic pathways were observed mainly in atopic dermatitis dogs. FUH may be able to improve intestinal microbiota and thus clinical signs of skin allergy.

**Abstract:**

Intestinal microbiota alterations were described in allergic individuals and may improve with diets. Farmina Ultra Hypo (FUH), a hydrolyzed fish/rice starch hypoallergenic diet, is able to improve clinical signs in allergic dogs. Study objectives were to determine microbiota differences in allergic dogs before and after feeding with FUH for eight weeks. Forty skin allergic dogs were evaluated clinically before and after the diet. Unresponsive dogs were classified as canine atopic dermatitis (CAD); responsive dogs relapsing after challenge with previous foods were classified as being food reactive (AFR), and those not relapsing as doubtful (D). Sequencing of feces collected pre- and post-diet was performed, with comparisons between and within groups, pre- and post-diet, and correlations to possible altered metabolic pathways were sought. Microbiota in all dogs was dominated by *Bacteroidota*, *Fusobacteriota*, *Firmicutes* and *Proteobacteria*, albeit with large interindividual variations and with some prevalence changes after the diet. In general, bacteria producing short-chain fatty acids were increased in all samples. CAD dogs showed pre-and post-diet microbiota patterns different from the other two groups. Bacteria taxa were enriched post-diet only in the AFR group. Changes in metabolic pathways were observed mainly in the CAD group. FUH may be able to improve intestinal microbiota and thus clinical signs of skin allergy.

## 1. Introduction

Adverse food reaction (AFR) and canine atopic dermatitis (CAD) are frequent dermatologic conditions in dogs: it is estimated that up to 30% of all dogs may be affected [1]. The most common dermatological signs of AFR and CAD are pruritus; self-induced alopecia due to scratching and rubbing; erythema of the muzzle, ventral areas and extremities; excoriations following scratching; possible otitis; and possible complicating bacterial and Malassezia dermatitis [2,3].

Currently, the best way to differentiate AD from AFR is to exclusively feed an 8-week-long hypoallergenic diet [4,5]. Hydrolyzed diets with starch are considered the most reliable ones for this purpose, as they are composed of very short peptides and amino acids that are not recognized by the immune system [6,7], while limited-antigen diets or diets containing whole cereals may not be as efficient due to extensive cross-reactions among food allergens [8,9,10].

Farmina Ultra Hypo (FUH) has proven to be a valuable aid as a diagnostic tool for AFR in dogs [11]. The diagnosis is made by observing the improvement of skin symptoms after the 8-week diet and the deterioration of skin lesions and itching after reintroduction of the old diet to which the dogs are allergic (provocation test). In the abovementioned study using FUH, a considerable number of dogs (60%) improved significantly with the diet; however, of these, many (30%) did not relapse with the provocation test, suggesting a beneficial action of the diet beyond a correction of the food allergy.

It has been shown in humans [12], and to a lesser extent in dogs [13], that a correct microbial balance in the gut (microbiota) is necessary for the health of the immune system in general, and that a correct bacteria–inflammatory cell interaction educates the immune system to the correct response to pathogens, food and environmental antigens [14]. Skin homeostasis and allostasis were found to be linked with gastrointestinal health, indicating a bidirectional relationship between the gut and the skin, and there is evidence to suggest that the metabolic and immunological effects of gut microbiome members can affect skin conditions [15]. Disruption of this mechanism by intestinal dysbiosis has been associated with development of allergies in humans [16] and in dogs [17], and preliminary data on intestinal dysbiosis in allergic dogs have also recently been published [18,19].

In dogs, the intestinal microbiota is greatly influenced by nutrition [20], and nutritional interventions may be prescribed (pre-, pro- and postbiotics as well as prescription diets) to correct intestinal dysbiosis [21]. Restoration of gut dysbiosis could improve signs of allergic skin disease, due to the tolerogenic influence of the virtuous bacterial flora on the immune system.

Several studies were published on the use of pre- and probiotics for human atopic dermatitis with controversial results [22,23], while only a few studies were performed in dogs [24,25]. Prescription diets in dogs are able to influence the fecal microbiome [26,27], but only one study evaluated the efficacy of a hypoallergenic diet, in association with an omega-3/omega-6-rich supplement, on fecal dysbiosis in atopic dogs [18]. No study has yet evaluated the effect of a prescription hydrolysate/starch diet on microbiota and its metabolome in allergic dogs and the correlation with improvement of clinical signs.

The aim of this study was to analyze the gut microbiota and microbiome of dogs with allergic skin disease and to evaluate the changes in the gut microbiota and metabolome after two months of FUH administration, in light of the clinical improvements obtained from the diet, in animals with a confirmed diagnosis of AFR, in those that improve with the diet but do not relapse upon discontinuation (considered “doubtful” (D), as described in Section 2.3 below) and in those with CAD.

Our hypotheses were:

1. Dogs with skin allergies have a different microbiota/microbiome from data published on healthy dogs.
2. A single pathological state (e.g., adverse food reaction, canine atopic dermatitis) could be associated with specific signatures in the gut microbiome.
3. The FUH diet is able to improve microbiota alterations, as it does with clinical symptoms, in skin allergic dogs.
4. Improvement of microbiota/microbiome alterations is significant in dogs that improve clinically, compared to dogs that do not improve on the diet.

## 2. Materials and Methods

### 2.1. Animals

A total of 40 client-owned dogs with signs of skin allergy were included in the study by two veterinary dermatologists, with modalities identical to those described in a previous study [11]. Briefly, dogs should have shown pruritis and/or lesions compatible with nonseasonal allergic dermatitis, having excluded parasitic diseases and skin infections.

### 2.2. Diet

Farmina Ultra Hypo is a commercially available hypoallergenic diet based on hydrolyzed fish and rice starch, supplemented with herring oil. It contains 53.5% carbohydrates, 18% protein, 15% fats, 1.2% fibers, 1.75% omega-3 fatty acids, 2.2% omega-6 fatty acids and 2.05% linoleic acid. It also contains powdered cellulose, potassium chloride, calcium carbonate, monocalcium phosphate, salt, vitamin A supplement, vitamin D3 supplement, vitamin E supplement, ascorbic acid, niacin, calcium pantothenate, riboflavin, pyridoxine hydrochloride, thiamine hydrochloride, biotin, folic acid, vitamin B12 supplement, choline chloride, beta-carotene, zinc methionine hydroxy analogue chelate, manganese methionine hydroxy analogue chelate, ferrous glycine, copper methionine hydroxy analogue chelate, inactivated selenium yeast, calcium iodate, DL-Methionine, taurine and mixed tocopherols (antioxidant).

### 2.3. Clinical Evaluation

Evaluation of dogs was performed at the time of inclusion (V1) and after 8 weeks of FUH administration (V2), with modalities already described in a previous publication [11]. Briefly, owners evaluated pruritus on a 10 cm long visual analogue scale (VAS) with descriptors [28], and veterinarians evaluated lesions by means of the Canine Atopic Dermatitis Lesions Index (CADLI; range 0–50) [29]. No systemic antibiotics were allowed in the two months before and during the food trial. The administration of prednisolone at 0.5–1 mg/kg was allowed for the first 10 days and oclacitinib 0.4–0.6 mg/kg for the first 20 days of the diet, as both were reported not to influence gut microbiota [19].

Whenever the owner-assessed pVAS and/or the CADLI lesion score had decreased by at least 50% compared to V1, owners were instructed to perform a diet provocation test with their old diets to confirm the diagnosis of AFR. In the case of relapse with the prior diet, followed by a new improvement with the elimination diet, the dogs were diagnosed with AFR. Dogs in which pruritus and/or lesions decreased by less than 50% were considered not to have AFR, but CAD. Dogs which improved but did not deteriorate after provocation were considered “doubtful” (D).

### 2.4. Fecal Collection and Analysis

Feces from all dogs were collected with a sterile swab on day V1 and on day V2 and stored in Norgen’s Stool Preservative (Fecal Swab Collection and Preservation System, NORGEN BIOTEK, Thorold, ON, Canada) until processed.

The Stool DNA Isolation Kit (NORGEN BIOTEK, Thorold, Canada) was used to extract DNA from stools. Extracted DNA was quantified with spectrophotometer and fluorimetric methods with Qubit™ dsDNA HS Assay Kit (ThermoFisher Scientific, Waltham, MA, USA). The V4 hypervariable region of the 16S rRNA gene was amplified by PCR. A subsequent limited-cycle amplification step was performed to add multiplexing indices and Illumina sequencing adapters (Illumina, San Diego, CA, USA).

Libraries were quantified with the above mentioned Qubit™ dsDNA HS Assay Kit and analyzed with High Sensitivity D1000 ScreenTape Assay kit on Agilent TapeStation 4200 instrument (Agilent, Santa Clara, CA, USA). Libraries were then sequenced on Illumina Miniseq instrument (Illumina, San Diego, CA, USA) with paired-end reads 150 bp long at a depth of 60 k cluster/sample.

Quality check was performed on FASTQ files, number of total reads and number of reads for each sample. FASTQ files were processed using available tools in QIIME 2 software (version 2020.6) (https://qiime2.org, accessed on 20 July 2023). Data were joined and quality filtered based on quality scores.

Representative sequence sets were used for taxonomy classification using VSEARCH global sequence alignment [30]. Silva reference databases were used for taxonomic classification (https://www.arb-silva.de/, accessed on 20 July 2023). OTU tables and taxa tables with abundance and taxonomy of each OTU for phylum, class, order, family and genus taxonomic level were produced.

### 2.5. Statistical Analysis

Analysis of alpha diversity (within sample) and beta diversity (between groups) of gut microbiota composition, based on OTU data, was performed for the three groups of dogs (AFR, CAD and D). Three multivariate statistical models were used: UniFrac distance matrices (weighted and unweighted) on the abundance of OTUs, and Bray–Curtis matrices and principal coordinate analysis (PcoA) plot on samples at the optimal rarefaction depth. More specifically, PCoA was performed based on Bray–Curtis distances, analysis of similarity (one-way ANOSIM) was performed with Bray–Curtis distance matrices, and indicator species analysis was performed with taxonomic analysis. The alpha diversity of the gut microbiota was assessed with Shannon’s diversity index (reflecting both richness and evenness). Statistical analysis of the differential abundance of species between experimental groups was performed with STAMP software package (Timberlake, London, UK) [31]. STAMP software was used to correlate taxonomic changes in the microbiota to possible altered metabolic pathways of microbiota that could induce dysbiosis. Pathway abundance analysis was performed with PICRUSt bioinformatics software package [32]. Comparisons were made within and between groups before and after the diet (atopic dermatitis, adverse food reaction and doubtful). To determine the differences in bacterial composition between the dog groups, a linear discriminant analysis effect size (LefSe) was utilized. LefSe is able to determine the features (organisms, clades, operational taxonomic units, genes or functions) most likely to explain the differences between classes by coupling standard tests for statistical significance with additional tests encoding biological consistency and effect relevance [33]. ANCOM-BC2 (Analysis of Compositions of Microbiomes with Bias Correction 2) software was used to perform differential analysis on compositional data among individual groups of dogs [34]. Significance was set at *p* < 0.05.

## 3. Results

### 3.1. Dogs

Forty dogs were included in the study and completed the eight-week-long trial with FUH. Eighteen were crossbreeds and 22 were purebreds. The mean age was 4.8 years (range: 8 months–12 years). There were 19 males (one of which was castrated) and 21 females (nine spayed). The mean weight was 22.5 kg (range 6–52 kg). Owners of 30/40 dogs reported the occurrence of occasional to regular gastrointestinal disturbances, such as vomit, regurgitation, diarrhea, soft feces, burping, flatulence or borborygmi. After completion of the eight-week diet, 9/40 dogs did not improve and were considered to have CAD. Thirty-one improved and proceeded with provocation. Of these, 20 showed a relapse of clinical signs with the provocation test and were therefore confirmed as AFR. Eleven dogs improved but did not relapse on the old diet and were considered “doubtful”. All data regarding the pre-and post-diet clinical scores, age of onset of clinical signs and concurrent gastrointestinal disturbances are reported in Table S1.

### 3.2. Analysis of Microbiota

One sample from the pre-diet CAD group was unsuitable for analysis due to loss of storage medium during transport. From the remaining 79 samples, a total of 11,301,718 high-quality demultiplexed sequences were obtained, with the number of reads ranging from 49,181 to 304,681 per sample (median 136,421, mean 143,060). Of the sequenced bases, 96.18% had a Q >= 30.

Rarefaction analysis and alpha diversity measures showed that the bacterial communities were sufficiently sampled, and further sequencing would be unlikely to significantly increase the observed microbial diversity detected (Figure S1).

#### 3.2.1. Alpha Diversity

**Figure 1:**
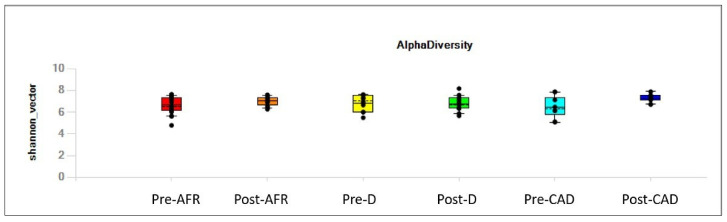
Alpha of the different groups, pre- and post-diet, assessed with Shannon’s diversity index (reflecting both richness and evenness). AFR = adverse food reactions; CAD = canine atopic dermatitis; D = doubtful diagnosis.

Alpha diversity, analyzed using the Shannon index (reflecting both richness and evenness) and considering the subject as well as the group (AFR, CAD or D), is depicted in Figure 1.

No appreciable difference was observed in gut microbiota alpha diversity between pre-diet samples of the three groups of dogs, with regard to evenness (Table 1), nor between pre- and post-diet samples for each group (Table 2) nor between post-diet samples of the three groups (Table 3) with regard to evenness.

**Figure 2:**
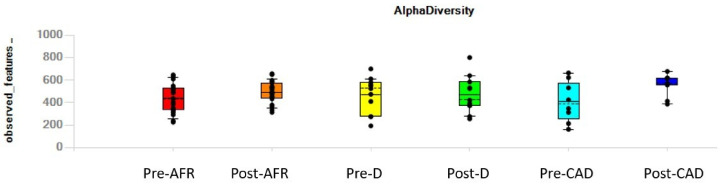
Individual variation in alpha diversity, as indicated in the value of observed features, pre- and post-diet. AFR = adverse food reactions; CAD = canine atopic dermatitis; D = doubtful diagnosis.

Individual dogs, irrespective of their pathologic condition, displayed variation in alpha diversity, as indicated in the value of observed features showed in Figure 2.

#### 3.2.2. Multivariate Comparisons of Gut Microbiota Composition

Differences (beta diversity) in the composition of the gut microbiota in pre- versus post-diet samples were verified for the three groups of dogs. Principal coordinate analysis on unweighted (considering presence/absence OTU) and weighted (community membership and abundance of OTUs) UniFrac distance matrices revealed significant differences in the microbial communities of AFR (*p* = 0.02) and CAD dogs (*p* = 0.018) (Table 4).

Permutational multivariate analysis of variance (PERMANOVA) (Adonis) analysis showed that the type of diet explained ~10% of the variability in beta diversity. No difference was seen in dogs from the D group (Table 4). Furthermore, no significant difference was seen between groups before the diet (Table 5) or after the diet (Table 6).

**Figure 3:**
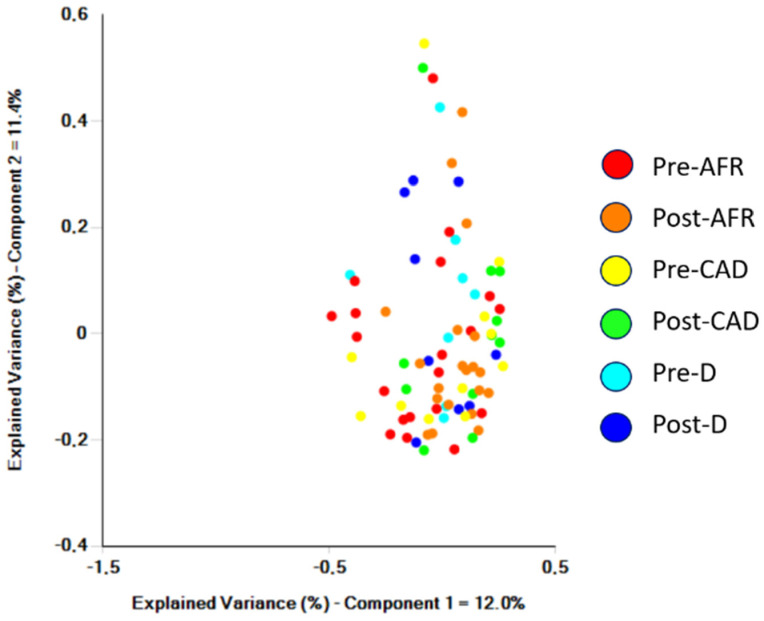
Samples from individual dogs analyzed by Bray-Curtis significance and clustered in a PCoA plot. AFR = adverse food reaction; CAD = canine atopic dermatitis; D = doubtful diagnosis.

Visual inspection of samples from individual dogs clustered in the PCoA plot indicated that the groups do not cluster (Figure 3).

**Figure 4:**
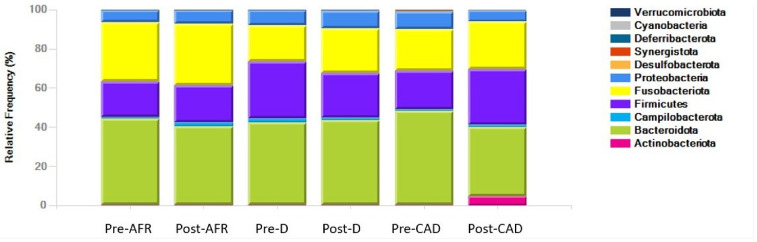
Phyla of bacteria present in three groups of dogs pre- and post-diet with pooled relative abundance. AFR = adverse food reaction; CAD = canine atopic dermatitis; D = doubtful.

**Figure 5:**
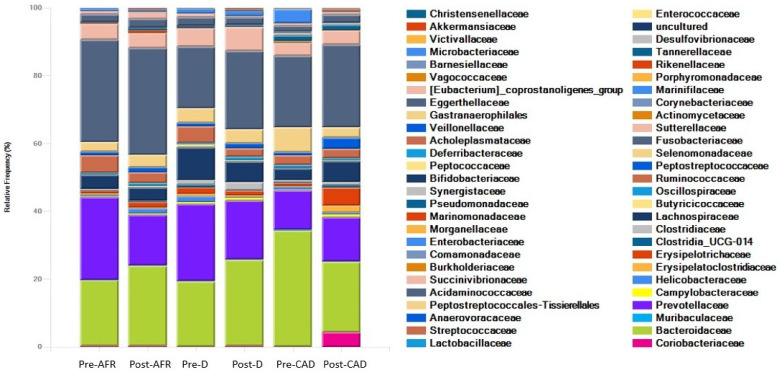
Families of bacteria present in three groups of dogs pre- and post-diet with pooled relative abundance. AFR = adverse food reaction; CAD = canine atopic dermatitis; D = doubtful.

**Figure 6:**
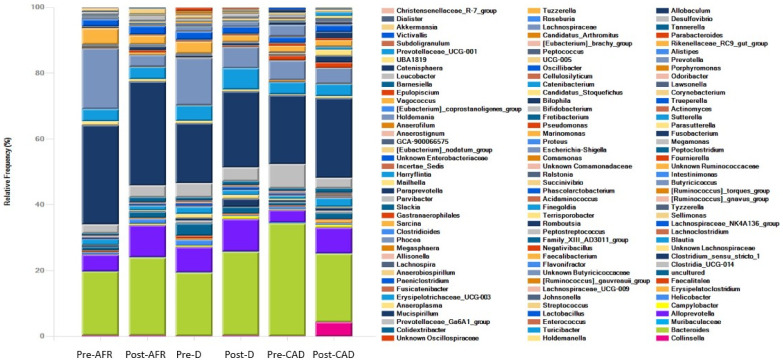
Genera of bacteria present in three groups of dogs pre- and post-diet with pooled relative abundance. AFR = adverse food reaction; CAD = canine atopic dermatitis; D = doubtful.

The phyla, families and genera present in the three groups of dogs are shown with pooled relative abundance in distribution graphs (Figure 4, Figure 5 and Figure 6).

OTU absolute and percentage abundance of bacterial species pre-and post-diet in the three groups are presented in Tables S2 and S3, respectively.

At the phylum level, the microbiota in all dogs was dominated by *Bacteroidota* (mean 42%), *Fusobacteriota* (mean 24.7%), *Firmicutes* (mean 22.9%) and *Proteobacteria* (mean 7.5%), albeit with large interindividual variations in relative abundance of these microbial taxa. In pre-diet samples, *Bacteroidota* were similar for all groups (42–48%), *Fusobacteria* were more abundant in AFR dogs than D and CAD dogs, while *Firmicutes* were more abundant in D dogs. In post-diet samples, *Fusobacteria* remained overall unchanged, while in CAD dogs, *Firmicutes* increased (*p*-value 0.22) and *Bacteroidota* and *Proteobacteria* were reduced (*p*-value 0.22). *Firmicutes* were also decreased in D dogs (Table 7).

At the family level, the dominant phylum for all dogs were *Bacteroidaceae* (mean 25.9%), *Fusobacteriaceae* (mean 24.7%) and *Prevotellaceae* (mean 17.4%). In pre-diet samples, *Bacteroidaceae* levels were higher and *Prevotellaceae* were lower in CAD dogs, while they were similar in the other two groups. *Fusobacteriaceae* levels were higher in pre-diet and post-diet samples in AFR dogs. In post-diet samples, *Fusobacteriaceae* increased mildly in all groups, *Bacteroidaceae* increased mildly in AFR and D dogs and decreased in CAD dogs, while the opposite was true for *Prevotellaceae* (Table 7).

At the genus level, the dominant genera for all dogs were *Fusobacterium* (mean 28.4%), *Bacteroides* (mean 23.9%), *Fecalibacterium* (mean 6.4%), *Prevotella* (mean 5.9%), *Alloprevotella* (mean 5%), *Megamonas* (mean 4.5%) and *Sutterella* (mean 3.1%). In CAD dogs only, 4.2% of bacteria of the genus *Collinsella* was present in post-diet samples (Table 7).

In pre-diet samples, the genus *Prevotella* was much less present in CAD dogs compared to AFR and D dogs (6% versus 14.5–18.5%) while *Bacteroides* was more prevalent (34.3 vs. 19%). After the diet, *Fusobacterium* increased slightly in all groups, *Prevotella* decreased and *Bacteroidetes* increased in AFR and D dogs but not in CAD dogs, in which *Bacteroides* decreased. Thus, the ratio of *Prevotella* + *Bacteroides/Fusobacterium*, relevant for a healthy dog gut microbiome [35], decreased importantly in all groups (Table 7).

**Figure 7:**
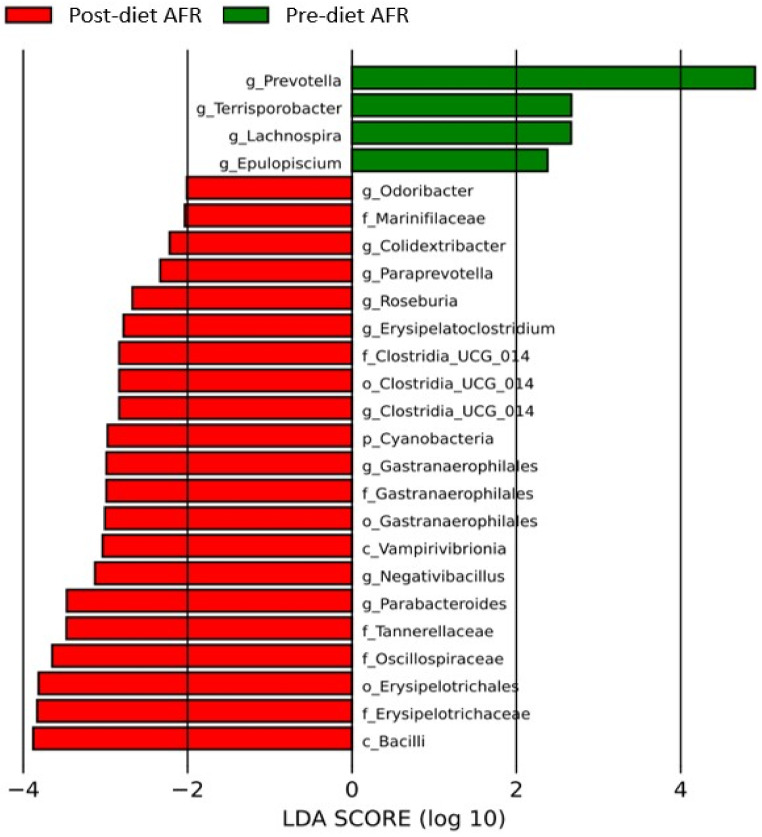
Histogram of the linear discriminant analysis (LDA) scores for differentially abundant bacterial clades in fecal samples of dogs in the AFR group pre-and post-diet. Negative (red bars) LDA scores represent bacterial groups overabundant in post-diet samples, while positive (green bars) represent bacterial groups overrepresented in pre-diet samples.

Evaluation of differences in bacterial composition by LefSe revealed that several bacterial taxa were found to be enriched in the three groups. After the diet, in the AFR group, the microbiota was enriched with two new classes (*Vampirivibrionia* and *Bacilli*), four orders, six bacterial families and nine bacterial genera, including *Clostridium* UCG 14 (*p*-value 0.03), *Paraprevotella* (*p*-value 0.04), *Roseburia* (*p*-value 0.03), *Parabacteroides* (*p*-value 0.001), *Erysipelatoclostridum* (*p*-value 0.02), *Bacillus* (*p*-value 0.04), *Lachnospira* (*p*-value 0.01) and *Epulopiscium* (*p*-value 0.04) (Figure 7 and Table S4).

**Figure 8:**
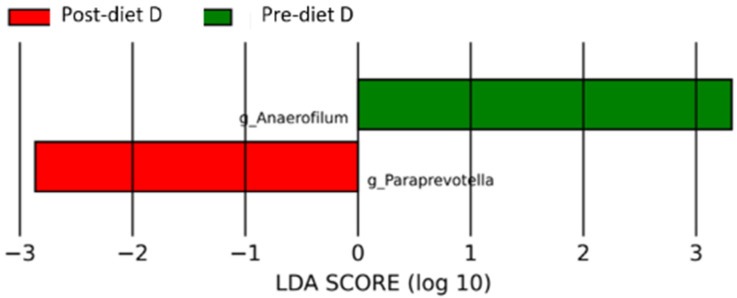
Histogram of the linear discriminant analysis (LDA) scores for differentially abundant bacterial clades in fecal samples of dogs in the D group pre-and post-diet. Negative (red bars) LDA scores represent bacterial groups overabundant in post-diet samples, while positive (green bars) represent bacterial groups overrepresented in pre-diet samples.

In the group of dogs with doubtful diagnosis (D), after the diet, the microbiota was enriched with the genera *Anaerofilum* (*p*-value 0.03) and *Prevotella* (*p*-value 0.03) (Figure 8 and Table S5).

**Figure 9:**
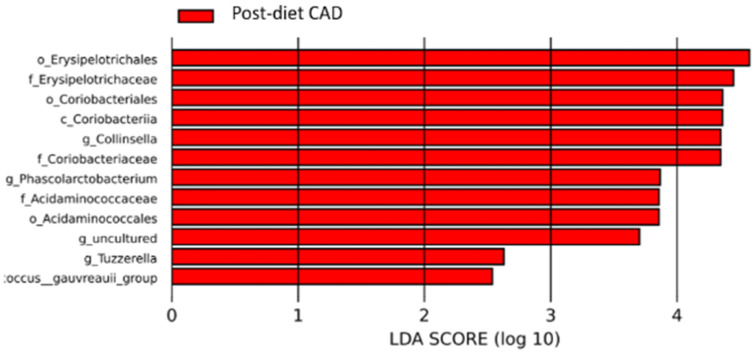
Histogram of the linear discriminant analysis (LDA) scores for differentially abundant bacterial clades in fecal samples of dogs in the CAD group pre-and post-diet. Negative (red bars) LDA scores represent bacterial groups overabundant in post-diet samples. In this case, there are no positive (green bars) representing bacterial groups overrepresented in pre-diet samples.

No microbiome enrichment was observed for the CAD group (Figure 9 and Table S6).

At the species level, the main post-diet changes reported in Table S2 are summarized in Table 8.

#### 3.2.3. Microbial Signatures Are Associated with Specific Pathological States

The fold change value of the pre-diet group versus the post-diet group was evaluated with ANCOM bias software. For the group of AFR dogs, numerous within-group log fold change fluctuations were observed, albeit with only one value statistically significant, related to bacteria of the *Tannarellaceae* family (q value 0.005), genus *Parabacteroides* (q-value 0.01) (Table S7). No significant changes were observed for the remaining two groups (Tables S8 and S9).

### 3.3. Analysis of Metabolic Pathways: Functional Changes in Gut Microbiota

After eating FUH for eight weeks, carbohydrate metabolic pathways decreased in all dogs (*p* = 0.01).

In CAD dogs, the following changes were observed:

- A pre-diet glycosphingolipid pathway deficiency, which improved significantly post-diet (*p* = 0.02).
- A post-diet increase in glycan biosynthesis (*p* = 0.04).
- A pre-diet deficiency in apelin, which also improved with the diet (*p* = 0.02).
- Post-diet increases in nucleoside and nucleotide biosynthesis (*p* = 0.04).
- A post-diet increase in amino acid biosynthesis (*p* = 0.03).
- Post-diet increases in fatty acid and lipid biosynthesis (*p* = 0.03).
- In AFR dogs, the following changes were observed:
- A post-diet reduction in lipid biosynthesis (*p* = 0.04).
- A post-diet increase in metabolic pathways of glycolysis from fructose (*p* = 0.02).

In D dogs, there was a post-diet increase in the biosynthesis of amines and polyamines (*p* = 0.03).

## 4. Discussion

This is the first study analyzing changes in fecal microbiota in dogs with skin allergies fed a diet based on hydrolyzed fish protein and rice starch.

### 4.1. General Pre-Diet Microbiota Findings: Do Dogs with Skin Allergies Have a Different Microbiota/Microbiome?

The main phyla of bacteria present in the fecal microbiota of our dogs is similar to what has been previously published: *Firmicutes*, *Proteobacteria*, *Fusobacteria* and *Bacteroidetes* are the four most represented phyla [36]. Prevalence of each phylum in our study and in previously published works can be very variable. In his original report, Suchodolski reported the majority of *Firmicutes* (47.7%), followed by *Proteobacteria* (23.3%), *Fusobacteria* (16.6%) and *Bacteroidetes* (12.4%) [36]. In our dogs, the microbiota in all dogs was dominated by *Bacteroidetes,* with a percentage similar to what was described by Rostaher and coworkers [19], albeit with higher levels of *Fusobacteria* (mean 24.6%) and lower of *Firmicutes* (mean 17.9%) and *Proteobacteria* (mean 5.9%). Allaway and coworkers [37], after feeding a complex commercial diet, reported a majority of *Bacteroides* (55%), followed by 20% *Firmicutes*, 10% *Fusobacteria* and 10% *Proteobacteria*, more in line with our results.

Due to the fact that only pruritic animals were sampled, and no healthy controls, we are not able to say if the microbiota in this population was different to that of healthy dogs. Rostaher and coworkers [19] found no significant difference at the phylum level between atopic and normal dogs, while they reported the families of *Anaerovoracaceae*, *Ruminococcaceae* and *Peptostreptococcaceae* to be significantly decreased in atopic versus healthy dogs. In our samples, the percentages of *Anaerovoracaceae* and *Peptostreptococcaceae* were similar to what was reported by Rostaher and coworkers in atopic dogs [19], while *Ruminococcacee* levels were higher in all our allergic dogs. At the genus level, Rostaher found *Catenibacterium* to be increased in atopic dogs [19]. This genus was observed, albeit in small amounts, in all our study groups, with an important decrease in post-diet samples.

More recently, Sikko et al. [38] also compared the microbiota in healthy and atopic dogs, reporting an increase in *Escherichia-Shigella* in atopic animals, suggesting that this increase could be the consequence of antibiotic use in allergic animals. Indeed, it is not always easy to determine if microbiota alterations in allergic dogs are the cause of the allergic condition or the consequence of the many medications these dogs receive for their dermatitis. Interestingly, in our study, *Escherichia-Shigella* was particularly increased in CAD dogs only, with a decrease in post-diet samples, suggesting that a higher isolation of *Escherichia-Shigella* in CAD dogs could be related to the atopic disease.

### 4.2. Comparison between Groups in Pre-Diet Samples: Can We Associate a Single Pathological State (e.g., Adverse Food Reaction, Canine Atopic Dermatitis) with Specific Signatures in the Gut Microbiome?

To the authors’ knowledge, this is the first time that microbiota is analyzed in food allergic dogs and compared to that of atopic ones. Different to what was described by Rostaher et al. [19], who observed a significantly different bacterial alpha diversity between healthy and allergic dogs, no difference was observed in our study between groups. This could be due to the fact that all animals in our study were allergic, even if the Shannon diversity value reported by Rostaher and coworkers [19] in their allergic dogs (about five) seems to be lower than what was observed in ours (about seven). Direct comparisons cannot be made, as the calculation methods and software may have been different.

No significant difference (beta diversity) was observed between groups in pre-diet samples. In general, atopic dogs showed lower amounts of phylum *Firmicutes* and genus *Prevotella* and higher amounts of phylum *Bacteroidetes* and family *Bacteroides*. *Firmicutes* play a significant role in the relationship between gut bacteria and health. Many of the members of this phylum break down complex carbohydrates such as dietary fiber, which cannot be digested by the gut, and resistant starch, with production of short-chain fatty acids (SCFAs), including propionate, acetate and butyrate, which help prevent inflammation and improve colonocyte health [13]. Different gut microbiome structures in allergic dogs may reflect an altered functional potential, because members of the gut microbiota can influence skin conditions through their metabolic activity and immunological impact [15]. SCFAs derived from fiber through the gut can influence the prevalence of certain microbial groups, which subsequently affects cutaneous immune defense mechanisms. For example, some commensal gut microbes can control T cell differentiation, including T-regs, which contribute to the skin immune system. Disruption of gut integrity and an imbalance within microbial communities can have a significant impact on the overall skin homeostasis if resident T-regs become less abundant, so a decrease in fermenting *Firmicutes* could possibly predispose dogs to developing a cutaneous allergic disease [39].

In AFR dogs, the gut microbiota contains more *Prevotella* species. *Prevotella* bacteria are able to break down a variety of polysaccharides, have the capacity to synthesize propionate and, conversely, lead to lower levels of butyrate. High levels of *Prevotella* were detected in stool samples from patients with rheumatoid arthritis, IBD, obesity and diabetes [40]. *Prevotella copri* revealed pathobiontic properties such as releasing inflammatory mediators from immune and stromal cells and promoting inflammatory diseases.

In this study, we also sought to identify specific microbiota signatures in the various groups of dogs that could explain the different responses to the diet and thus the final diagnosis (AFR vs. CAD), allowing a prediction of the response to the diet based on baseline signatures and clinical characteristics [41]. Only pre-diet AFR dogs showed a microbiota deficient in genus *Paraacteroides* bacteria, with an increase after the diet. *Parabacteroides* consists of Gram-negative, obligate anaerobes. At present, the link between *Paraacteroides* deficiency and skin allergies has not yet been identified, although in a recent study, *P. distasonis* presence was found to be significantly decreased in psoriatic patients, compared to non-psoriatic patients [42]. More patients probably need to be analyzed to obtain statistically significant results in the determination of microbiota signatures in allergic dogs.

### 4.3. Response to the Diet and Impact on Metabolic Pathways: Is FUH Able to Improve Microbiota Alterations in Skin Allergic Dogs?

The carbohydrate source of FUH is rice starch (RS). RS, a dietary fiber, has been documented to offer numerous health advantages, notably in lowering the risk of chronic diseases like obesity and diabetes. These favorable impacts can be attributed to alterations in the gut bacteria population and the production of microbial-generated metabolites, such as short-chain fatty acids (SCFAs), particularly butyrate and acetate [43]. Colonocytes primarily utilize butyrate as their primary energy source, while acetate and propionate are transported to the liver through the portal vein. In the liver, propionate is metabolized for gluconeogenesis, while acetate serves as a substrate for cholesterol synthesis and lipogenesis. Additionally, acetate is taken up by muscle and adipose tissue. Our study revealed that a diet high in RS resulted in increased production of SCFA-producing bacteria, such as *Bacteroides*, in the luminal content. However, this effect was observed only in AFR and D dogs.

In the diet fed, Farmina Ultra Hypo, the protein source is hydrolyzed fish, composed of small peptides or single amino acids that decrease the probability of an immune response to protein dietary components. In spite of feeding an 8-week course of a hydrolyzed-protein-based diet exclusively, the bacterial diversity did not seem to be affected, as no significant variation in the Shannon index was observed. A similar result was obtained in other studies in which hydrolyzed protein/crystallized amino acid and starch diets were fed to healthy dogs for at least 4 weeks [26,31].

On the contrary, analysis of microbiota differences (beta diversity) between pre-and post-diet samples found significant differences for the AFR and CAD groups. In post-diet samples in all groups, there was a decrease in the phylum *Bacteroidetes*, and the reduction was driven by the group of *Bacteroides*. Bacteria in the phylum *Firmicutes* increased significantly only in CAD dogs, while *Proteobacteria*, family *Enterobacteriaceae* were reduced (*p*-value 0.22).

In a previous study in which dogs were fed a similar hydrolyzed proteins/starch diet to healthy dogs, changes obtained were partly similar to ours: a decrease in *Bacteroidetes* and an increase in *Fusobacteria* were reported, while *Firmicutes* were unchanged and *Proteobacteria* were decreased [31]. At the family level, the same study reported an increase in Bacteroidacee and a decrease in *Prevotellacee*. This pattern was also observed in our dogs, with the exception of CAD dogs, in which this pattern was reversed.

At the genus level, only AFR and D dogs showed a high *Prevotella*/*Bacteroides* ratio, which decreased substantially with the diet, with *Prevotella* decreasing and *Bacteroides* and *Fusobacteria* increasing in both groups. *Bacteroides* is known for its exceptional capacity to digest carbohydrates and its remarkable adaptability to swiftly changing environments. The rise in *Bacteroides* levels is primarily responsible for the reduction in species richness and diversity, as well as the enrichment of glycolytic metabolism that has been observed [44].

The abovementioned *Prevotella*/*Bacteroides* ratio was very different in the CAD group, where the ratio was already low at the beginning due to very high *Bacteroides* levels (double the other groups) and lower *Prevotella* (less than half of the other groups), and the ratio was only minimally increased with the diet.

The ratio of *Bacteroides* + *Prevotella*/*Fusobacteria*, which is considered relevant for a healthy canine gut [35], decreased importantly in all groups. However, CAD dogs seem to have a different microbiota pattern and different responses when compared to AFR and D dogs, because at the genus level, *Bacteroides* increase in AFR and D dogs, while they decrease in CAD dogs.

Interestingly, a completely different pattern was observed in the microbiota of dogs fed another hydrolyzed feather/corn starch diet (Royal Canin Anallergenic^®^) by Mori and coworkers [26], who observed very high amounts of *Firmicutes* (mean 71.7%), much higher than *Bacteroidetes* (23.9%), and extremely low amounts of *Fusobacteria* (0.4%). This difference could be driven by a different dietary composition, in particular by higher carbohydrates and fiber in the diet used in the abovementioned study.

Indeed, the main changes in the microbiota after a dietary trial depend on the amount of proteins, fats, carbohydrates and fibers present in the diet. As mentioned before, *Firmicutes* favor high fiber and carbohydrate content [27]; among them, there are *Lactobacilla* and other beneficial bacteria that produce butyrate, in particular *Faecalibacterium*, *Eubacterium*, *Roseburia* and *Anaerostipes*. Butyrate offers numerous health benefits due to its multiple functions: it serves as an energy source for the cells lining the gut, stimulates the production of glutathione (a natural antioxidant), regulates intestinal inflammation and promotes a resilient gut lining. Additionally, it plays a role in enhancing the memory potential of activated CD8+ T cells by influencing their cellular metabolism. Moreover, butyrate aids in the prevention of cancer by impeding the development of neoplastic cells and supports the production of hormones for a healthy metabolism [45]. *Firmicutes* were particularly enriched in the AFR group of dogs, among these Roseburia, known to produce butyrate that, together with the short-chain fatty acids of the fish oil added to FUH, has the potential to improve the defense barrier of the intestinal lining, including tight junctions.

*Fusobacteria* are known to be increased in dogs fed raw meat [27], which is low in carbohydrates and fiber. Interestingly, *Fusobacteria* levels were much higher in pre-diet AFR and D samples compared to CAD dogs. Due to an unknown previous dietary history, it is not known if one or more dogs in the former two groups were eating BARF diets and thus could have skewed the data. In any case, the higher amount of *Fusobacteria* remained unchanged during the study, after feeding the commercial diet containing both carbohydrates and fiber. *Fusobacterium* abundance is increased in dogs with access to the outdoors, and higher levels of *Fusobacterium* are also seen in other carnivore species [13].

### 4.4. CAD Dogs Are Different: Are Microbiota/Microbiome Alterations Significant in Dogs That Do Not Improve on the Diet?

We have already described in the paragraphs above how in many aspects CAD dogs show several differences to AFR and D dogs, and how D dogs seem to be more similar to AFR than CAD dogs. It may be that D dogs were not challenged for long enough or with the correct offending food to show a relapse and be categorized in the AFR group.

On the other hand, CAD dogs presented a different microbiome pattern before the diet and showed a different change dynamic and no enrichment after the diet, but on the contrary, useful *Firmicutes* like *Lactobacillus* and *Collinsella* decreased compared to the other groups.

Microbiota enrichment is key for improvement of atopic disease at the skin level, and it is possible that this is true for the gut microbiota too [46]. CAD dogs could thus benefit from probiotics to improve their cutaneous disease, as has already been suggested by early studies performed by Marsella et al. [24,25].

Atopic dermatitis (AD) in humans is a chronic inflammatory skin condition with a multifactorial pathogenesis. Inflammatory bowel disease (IBD) can be accompanied by skin lesions [47]. It has been suggested that skin conditions associated with IBD may stem from immune dysregulation, leading to a destructive process mediated by lymphocytes [48]. T cells present in the gut mucosa may migrate to the skin, become exposed to cutaneous antigens and contribute to skin damage [49]. Emerging evidence supports the existence of a gut–skin axis, which is influenced by neuroendocrine molecules produced by the gut microbiome [39]. These molecules have the potential to affect both skin barrier dysfunction and immune system dysregulation, which are key factors in the development of atopic dermatitis (AD).

In our study, we observed a deficiency of apelin in dogs with AD before the dietary intervention. Apelin acts as an endogenous ligand for the APJ receptor, a seven-transmembrane G-protein-coupled receptor. Apelin and APJ receptors are distributed in various tissues, including the heart, lungs, liver, kidney and gastrointestinal tract. Previous research has shown that apelin messenger RNA is widely expressed in gastrointestinal (GI) tissues, particularly in the stomach and small intestine, and is closely associated with GI function. Under normal conditions, the apelin/APJ system plays diverse biological roles, such as gastric acid secretion, appetite control, cell proliferation and apoptosis, CCK secretion, histamine release, pancreatic juice secretion, GI motility and gut–brain axis regulation. The apelin/APJ system also plays significant roles in pathological conditions, acting as a potential gastric injury protectant, a marker for gastric and colon cancer, a lipid regulator for nonalcoholic fatty liver disease (NAFLD) and a mediator of fibrosis. Recent animal studies have reported that an increased intake of fatty acids and eicosapentaenoic acid (EPA) leads to increased apelin expression and concentration [48].

In dogs with canine atopic dermatitis (CAD), we observed a deficiency in glycosphingolipid pathways before the dietary intervention, which significantly improved following the diet. Recent research has proposed that sphingolipids and the enzymes involved in their metabolism may play a role in the development of allergic diseases. Sphingolipids are crucial for cell growth, survival, inflammation and tissue remodeling. Biologically, sphingolipids are primarily found in the plasma membrane, intracellular organelles and lipoproteins, acting as a reservoir of bioactive metabolites involved in signaling, cell survival and growth, immune cell trafficking and vascular and mucosal integrity [50]. In areas affected by AD lesions, epidermal barrier dysfunction is evident, characterized by increased trans-epidermal water loss, elevated skin pH, altered surface microbiota colonization patterns and an affected ceramide profile. The compromised barrier function in AD leads to the continuous generation of cytokines, chemokines, proinflammatory cytokine cascades, and exposure to allergens and antigens, all of which contribute to the “atopic march.” The reduction or alteration of sphingolipid composition in the epidermis not only contributes to impaired skin barrier function but also promotes the development of inflammatory and allergic properties in individuals with AD [48].

While the majority of bacterial species lack the ability to produce sphingolipids, *Bacteroides*, a prominent commensal microbiome in the intestine, possesses the capability to produce and supply ceramides. These ceramides play a role in dampening inflammatory responses and contributing to the maintenance of intestinal immune system homeostasis. On the other hand, host-derived sphingolipids from the intestine aid in preserving *Bacteroides* species or regulating their abundance through the bactericidal activity of sphingosine. In contrast, the uncontrolled proliferation of pathogenic bacteria like *Pseudomonas*, *Staphylococcus* or *Mycobacterium* can disrupt the balance of the bacterial flora and hinder host sphingolipid metabolism [51]. In our study, post-dietary intervention, we observed an increase in *Bacteroides* species in AFR and D dogs, while CAD dogs showed a reduction in *Bacteroides* levels.

### 4.5. AFR Dogs and D Dogs: Are Microbiota/Microbiome Alterations Significant in Dogs That Do Improve on the Diet?

In AFR dogs, there is a post-diet increase in the glycolysis metabolic pathway from fructose and an enrichment of *Bacilli, Roseburia, Parabacteroides, Lachnospira, Erysipelatoclostridum, Clostridium* UCG 14 and bacteria of *Vampirivibrio* class.

Fructolysis, a process similar to glycolysis, utilizes many of the same enzymes and metabolic intermediates. However, fructose differs in that it enters glycolysis without the energy investment step, resulting in an additional ATP yield. Unlike glucose, which undergoes metabolism throughout the body, fructose is primarily metabolized in the liver. Its metabolism in the liver primarily contributes to replenishing liver glycogen and synthesizing triglycerides. Fructose facilitates the uptake and storage of glucose in the liver, accelerates the oxidation of carbohydrate stores after a meal, supplies the majority of energy required for spermatozoa mobility and potentially plays a crucial role in the maturation of preadipocytes, enabling them to store more fat. Furthermore, fructose may benefit individuals involved in intense physical activity by supporting hepatic gluconeogenesis and providing additional energy for skeletal muscle contraction in the form of lactate [52].

Our metagenomics study suggests that dogs in the AFR group have, after being fed the FUH diet, an increased ability to utilize sugars using an improved metabolic pathway and that the organic components of the diet provide a favorable substrate for the growth of butyrate- and acetate-producing bacteria. One possibility is that the metabolism of fructose may confer a greater advantage for *Bacilli* and intestinal butyrate–acetate producing bacteria. Indeed, microbes depend on their community to support all biological activities essential for their metabolism.

Also, in the group of doubtful dogs, even if it was much lower, an increase in species *Anaerofilus*, belonging to the *Lachnospiranacee* family, was observed, with genes related to acetogenesis, capable of reducing fructose to acetate.

### 4.6. Weaknesses of This Study

There are several weaknesses of this study. One is the low number of dogs for each group, although similar studies were performed with even lower numbers [19]. The dietary history of the dogs was not known or recorded, and this could have influenced the initial microbiota pattern and results. Even if being a confounding element, different dietary intakes reflect everyday field work, in which allergic animals may have eaten several different diets before being brought to the dermatologist. The duration of the diet, 8 weeks, may not be long enough to obtain a clear change in microbiota, even if changes were observed in just 4 weeks in several other studies [27].

## 5. Conclusions

These preliminary data suggest that there are pathological alterations in the microbiota and metabolome in allergic dogs and that FUH can correct these, and perhaps, that this may also be reflected in the improvement of dermatological symptoms. FUH containing rice starch modified the microbiota, favoring the growth of bacteria producing short-chain fatty acids, important for intestinal epithelial barrier integrity and colonocyte activity. The FUH diet also reduced the prevalence of *Bacteroidetes*, typical of dysbiotic dogs, and *Prevotella*, usually common in chronic enteric diseases.

## Figures and Tables

**Table 1:** Alpha diversity between groups before the diet assessed by Shannon’s diversity index (reflecting both richness and evenness). There is no significant diversity between any of the groups.

Pre-Diet Group Comparison		H	*p* Value	Q Value
AFR (*n* = 20)	CAD (*n* = 8)	0.093	0.760	0.828
AFR (*n* = 20)	D (*n* = 11)	0.334	0.563	0.768
CAD (*n* = 8)	D (*n* = 11)	0.334	0.563	0.768

AFR = adverse food reaction; CAD = canine atopic dermatitis; D = doubtful diagnosis.

**Table 2:** Alpha diversity between pre- and post-diet samples for each group assessed by Shannon’s diversity index (reflecting both richness and evenness). There is no significant diversity between any of the groups.

Pre- and Post-Diet Comparison	H	*p* Value	Q Value
AFR (*n* = 20)	1.686	0.194	0.495
CAD (*n* = 8)	2.676	0.102	0.495
D (*n* = 11)	0.243	0.622	0.778

AFR = adverse food reaction; CAD = canine atopic dermatitis; D = doubtful diagnosis.

**Table 3:** Alpha diversity between groups after the diet assessed by Shannon’s diversity index (reflecting both richness and evenness). There is no significant diversity between any of the groups.

Post-Diet Group Comparison	H	*p* Value	Q Value
CAD (*n* = 9)	2.136	0.144	0.495
D (*n* = 11)	1.434	0.231	0.495
D (*n* = 11)	3.188	0.074	0.495

AFR = adverse food reaction; CAD = canine atopic dermatitis; D = doubtful diagnosis.

**Table 4:** Evaluation of beta diversity: differences in the composition of the gut microbiota before and after the diet in the three groups of dogs verified by UniFrac significance ANOSIM. R values close to 1 indicate high separation between groups. R values close to 0 indicate similarity between groups.

		Weighted			Unweighted		
Group	Sample Size	*p*-Value	q-Value	R	*p*-Value	q-Value	R
AFR	40	0.01	0.08	0.09	0.01	0.04	0.10
CAD	17	0.04	0.16	0.10	0.03	0.11	0.18
D	22	0.94	0.94	−0.06	0.91	0.91	−0.06

AFR = adverse food reaction; CAD = canine atopic dermatitis; D = doubtful diagnosis.

**Table 5:** Evaluation of beta diversity: differences in the composition of the gut microbiota before the diet between the three groups of dogs verified by UniFrac significance ANOSIM. R values close to 1 indicate high separation between groups. R values close to 0 indicate similarity between groups.

		Weighted			Unweighted		
Group	Sample Size	*p*-Value	q-Value	R	*p*-Value	q-Value	R
Pre-AFR × Pre-CAD	28	0.26	0.64	0.05	0.07	0.21	0.18
Pre-AFR × Pre-D	31	0.62	0.77	−0.04	0.66	0.76	−0.04
Pre-CAD × Pre-D	19	0.76	0.81	−0.06	0.16	0.30	0.06

AFR = adverse food reaction; CAD = canine atopic dermatitis; D = doubtful diagnosis.

**Table 6:** Evaluation of beta diversity: differences in the composition of the gut microbiota after the diet between the three groups of dogs verified by UniFrac significance ANOSIM. R values close to 1 indicate high separation between groups. R values close to 0 indicate similarity between groups.

		Weighted			Unweighted		
Group	Sample Size	*p*-Value	q-Value	R	*p*-Value	q-Value	R
Post-AFR × Post-CAD	29	0.62	0.77	−0.04	0.30	0.37	0.04
Post-AFR × Post-D	31	0.17	0.50	0.07	0.08	0.21	0.11
Post-CAD × Post-D	20	0.59	0.77	−0.02	0.75	0.80	−0.04

AFR = adverse food reaction; CAD = canine atopic dermatitis; D = doubtful diagnosis.

**Table 7:** Main percentage modifications of bacterial groups at the phylum, family and genus level.

**Phylum**	**AFR Pre**	**AFR Post**	**D Pre**	**D Post**	**CAD Pre**	**CAD Post**
*Bacteroidota*	44.1	40.1	42.1	43.4	47.8	35.5
*Fusobacteriota*	30.2	31.5	18.2	23.1	21.0	24.3
*Firmicutes*	18.3	19.0	29.2	22.6	19.7	28.5
*Proteobacteria*	6.5	6.9	8.1	9.1	9.2	5.3
**Family**	**AFR pre**	**AFR post**	**D pre**	**D post**	**CAD pre**	**CAD post**
*Bacteroidaceae*	19.5	23.6	19.1	25.4	34.1	30.8
*Prevotellaceae*	24.7	14.9	22.7	17.4	11.4	13.1
*Fusobacteriaceae*	30.2	31.5	18.2	23.1	21	24.3
**Phylum**	**AFR pre**	**AFR post**	**D pre**	**D post**	**CAD pre**	**CAD post**
*Prevotella*	18.5	3.7	14.5	6.4	6.0	4.9
*Bacteroides*	19.5	23.6	19.1	34.1	34.2	20.7
*Fusobacteria*	30	31	18	23	21	24
*Prevotella*/*Bacteroides* ratio	0.95	0.16	0.76	0.19	0.18	0.24
*Prevotella + Bacteroides*/*Fusobacteria* ratio	1.2	0.7	1.8	1.3	1.9	1

AFR = adverse food reaction; CAD = canine atopic dermatitis; D = doubtful diagnosis. The bold is the legend of the X-axis.

**Table 8:** Major changes in bacterial amounts by species after the diet for the three groups of dogs.

Bacteria Species	AFR	D	CAD
*Phascolarbacterium*	↑	↑	↑
*Prevotella*	↓	↓	↓
*Succinivibrio*	↓	↓	↓
*Erysipelotrichia*	↑	↑	↑↑
*Faecalibacterium*	↓	↓	=
*Phascolarbacterium*	↑	=	↑
*E. coli*/*shigella*	↓	=	↓
*Lattobacillus*	=	↑	↓
*Sutterella*	=	↑	↑
*Collinsella*	=	=	↓
*Anaerobiumspirillum*	=	=	↓

AFR = adverse food reaction; CAD = canine atopic dermatitis; D = doubtful diagnosis. ↑: increased; ↓: decreased; =: unchanged. ↑↑: highly increased.

## Data Availability

The data presented in this study are available in this article and supplementary material.

## Supplementary Materials

The following supporting information can be downloaded at https://www.mdpi.com/article/10.3390/vetsci10070478/s1, Figure S1: Chao rarefaction graph of 79 fecal samples showed that the bacterial community was sufficiently analyzed, Table S1: pre-and post-diet clinical scores, age of onset of clinical signs and concurrent gastrointestinal signs in 40 dogs with allergic skin disease, Table S2: OTU absolute counts of bacterial species pre-and post-diet in the three groups, Table S3: OTU percent abundance of bacterial species in pre- and post-diet fecal samples, Table S4: evaluation of differences in bacterial composition by LefSe in the adverse food reaction (AFR) group, Table S5: evaluation of differences in bacterial composition by LefSe in the doubtful (D) group, Table S6: evaluation of differences in bacterial composition by LefSe in the canine atopic dermatitis (CAD) group, Table S7: analysis of fold change value of the pre-diet adverse food reaction (AFR) group versus the post-diet group, Table S8: analysis of fold change value of the pre-diet doubtful (D) group versus the post-diet group, Table S9: analysis of fold change value of the pre-diet canine atopic dermatitis (CAD) group versus the post-diet group.
