# Supplementary material for: Analysis of Intestinal Microbiota and Metabolic Pathways before and after a 2-Month-Long Hydrolyzed Fish and Rice Starch Hypoallergenic Diet Trial in Pruritic Dogs
Source: Vet Sci. 2023 Jul 21;10(7):478. doi: 10.3390/vetsci10070478 (PMC10384699; doi:10.3390/vetsci10070478)
Supplement: Supplementary file 1 [file vetsci-10-00478-s001.zip › Table S1.pdf]

Case n.	age onset pruritus in months	number of daily defecations	recurrent soft faeces	occasional to frequent vomit or regurgitation	intestinal noise (N), flatulence (F), burping (B)	otitis	anal pruritus
2	3 m	0	no	no	F	no	no
3	5 m	1	no	no	F	yes	no
4	24 m	0	no	yes	F	yes	no
8	7 m	0	no	yes	F	yes	yes
13	27 m	0	no	yes	N, F, B	yes	yes
15	6 m	1	yes	no	F,B	yes	no
16	12 m	0	yes	yes	F,B	no	no
17	72 m	0	no	no	no		no
18	4 m	0	yes	no	B	yes	no
19	not nota	0	yes	no	N, F	yes	no
20	24 m	0	yes	yes	no	no	no
22	24 m	0	no	yes	N, F	yes	yes
23	6 m	0	no	yes	N, F	yes	yes
25	30 m	0	no	no	no	no	yes
28	12 m	1	no	no	no	no	no
30	24 m	1	yes	no	N, F, B	yes	yes
34	36 m	0	yes	no	n.d.	no	no
36	11 m	0	no	no	N, F	yes	no
39	12 m	0	no	no	F	yes	no
46	3,5 m	0	no	no	no	yes	yes
5	6 m	0	yes	no	F, B	yes	no
21	72 m	1	no	no	F	no	no
27	6 m	0	yes	yes	B, F	yes	yes
29	12 m	0	no	no	no	yes	yes
32	4 m	n.d.	n.d.	yes	B	yes	no
35	3 m	0	no	no	F	yes	no
41	12 m	0	no	yes	F, B	no	no
45	4 m	0	yes	yes	F, B	yes	no
47	4 m	0	yes	no	no	no	no
1	30 m	0	yes	no	N	no	yes
9	4 m	0	yes	no	N, F	yes	yes
10	12 m	1	no	no	no	yes	yes
14	82 m	0	no	no	no	yes	yes
24	9 m	0	no	no	N, F,B	yes	yes
26	4 m	0	no	no	no	yes	no
33	48 m	0	no	no	B	yes	yes
38	11 m	0	no	no	no	yes	no
40	60 m	0	yes	no	F	yes	no
42	96 m	0	no	no	no	no	no
44	6 m	0	yes	no	F, B	yes	no

CADLI V1	CADLI V2	VAS V1	VAS V2	Results after the diet	Results of provocation	Diagnosis
14	2	7.5	1,5	improved	relapsed	AFR
5	3	6.5	3	improved	relapsed	AFR
1	0	3.5	0	improved	relapsed	AFR
6	2	7.5	1.5	improved	relapsed	AFR
11	10	4	2	improved	relapsed	AFR
3	0	6	2	improved	relapsed	AFR
0	0	5.5	1	improved	relapsed	AFR
7	2	4	3.5	improved	relapsed	AFR
5	1	4.5	1.5	improved	relapsed	AFR
8	2	5.5	0	improved	relapsed	AFR
2	1	3.5	2.5	improved	relapsed	AFR
3	1	7	1.5	improved	relapsed	AFR
5	1	5.5	1.5	improved	relapsed	AFR
0	0	5,5	2	improved	relapsed	AFR
5	0	7.5	1	improved	relapsed	AFR
3	0	2	0	improved	relapsed	AFR
9	8	3.5	1	improved	relapsed	AFR
7	0	2	1.5	improved	relapsed	AFR
15	6	8.5	5	improved	relapsed	AFR
10	3	5.5	3.5	improved	relapsed	AFR
9	8	5	5	not improve(	not relapsed	CAD
2	1	7.5	4	not improve(	not relapsed	CAD
10	7	7.5	7.5	not improve(	not relapsed	CAD
16	9	5.5	3	not improve(	not relapsed	CAD
12	12	8	8	not improve(	not relapsed	CAD
16	13	4.5	4	not improve(	not relapsed	CAD
0	0	5.5	5.5	not improve(	not relapsed	CAD
3	4	5.5	5.5	not improve(	not relapsed	CAD
5	3	6	8	not improve(	not relapsed	CAD
3	0	5	1	improved	not relapsed	doubtful
2	1	8	4,5	improved	not relapsed	doubtful
4	0	5,5	3.5	improved	not relapsed	doubtful
11	8	8	3	improved	not relapsed	doubtful
6	3	1,5	1.5	improved	not relapsed	doubtful
7	1	3.5	0	improved	not relapsed	doubtful
4	0	6	2,5	improved	not relapsed	doubtful
2	0	5	2	improved	not relapsed	doubtful
9	0	6	0	improved	not relapsed	doubtful
5	2	8	0	improved	not relapsed	doubtful
3	1	5	4.5	improved	not relapsed	doubtful
