# Supplementary material for: Analysis of Intestinal Microbiota and Metabolic Pathways before and after a 2-Month-Long Hydrolyzed Fish and Rice Starch Hypoallergenic Diet Trial in Pruritic Dogs
Source: Vet Sci. 2023 Jul 21;10(7):478. doi: 10.3390/vetsci10070478 (PMC10384699; doi:10.3390/vetsci10070478)
Supplement: Supplementary file 1 [file vetsci-10-00478-s001.zip › Table S2.pdf]

Table S2: OTU absolute counts of bacterial species pre-and post-diet in the three groups. ARF = adverse food reaction; CAD = canine atopic dermatitis; D = doubtful

OTU absolute counts	Pre-AFR	Post-AFR	Pre-D	Post-D	Pre-CAD	Post-CAD
Collinsella	4.365.000	4.500.000	3.618.182	4.963.636	3.437.500	92.933.333
Bacteroides	311.275.000	380.875.000	426.627.273	538.763.636	763.225.000	449.244.444
Muribaculaceae	490.000	1.605.000	1.272.727	2.963.636	6.787.500	2.566.667
Alloprevotella	79.960.000	156.660.000	170.272.727	209.381.818	83.287.500	169.122.222
Campylobacter	3.285.000	7.185.000	10.727.273	16.781.818	6.500.000	17.933.333
Helicobacter	7.700.000	22.840.000	38.345.455	7.645.455	16.700.000	11.166.667
Erysipelatoclostridium	2.950.000	3.460.000	8.327.273	3.872.727	2.650.000	19.955.556
Faecalitalea	5.555.000	3.315.000	21.236.364	6.309.091	4.237.500	8.755.556
uncultured	18.540.000	27.670.000	82.772.727	41.800.000	18.012.500	38.388.889
Clostridia_UCG-014	1.255.000	3.150.000	18.236.364	3.409.091	6.650.000	16.866.667
Clostridium_sensu_stricto_1	5.685.000	2.315.000	18.554.545	49.600.000	12.412.500	17.044.444
Blautia	25.885.000	20.245.000	47.618.182	30.627.273	20.125.000	60.144.444
Lachnoclostridium	1.840.000	2.240.000	6.727.273	3.400.000	6.275.000	9.633.333
Lachnospiraceae_NK4A136_group	5.850.000	6.750.000	14.363.636	18.709.091	10.012.500	5.766.667
Sellimonas	285.000	450.000	163.636	354.545	600.000	311.111
Tyzzerella	840.000	1.790.000	1.700.000	781.818	200.000	2.300.000
[Ruminococcus]_gnavus_group	6.115.000	3.365.000	12.536.364	3.781.818	12.262.500	4.622.222
[Ruminococcus]_torques_group	1.075.000	2.015.000	881.818	3.036.364	362.500	4.333.333
Butyricicoccus	995.000	945.000	3.845.455	2.527.273	1.087.500	1.577.778
Intestinimonas	345.000	390.000	663.636	254.545	575.000	511.111
Fournierella	510.000	425.000	0.81818	627.273	225.000	700.000
Peptoclostridium	8.225.000	22.780.000	17.027.273	22.736.364	15.350.000	28.722.222
Megamonas	45.565.000	58.765.000	97.272.727	87.863.636	165.000.000	68.177.778
Fusobacterium	482.140.000	507.370.000	404.018.182	489.718.182	469.200.000	525.244.444
Parasutterella	17.100.000	11.925.000	16.000.000	14.036.364	6.575.000	10.877.778
Sutterella	59.570.000	60.185.000	106.045.455	137.972.727	81.400.000	82.066.667
Actinomyces	0.00000	0.00000	0.00000	0.00000	175.000	0.00000
Trueperella	0.00000	0.00000	0.00000	0.00000	200.000	0.00000
Corynebacterium	0.00000	0.00000	0.00000	0.00000	3.325.000	144.444
Lawsonella	0.00000	0.00000	0.00000	0.00000	137.500	0.00000
Odoribacter	0.00000	225.000	0.00000	0.00000	437.500	0.00000
Porphyromonas	0.00000	145.000	0.18182	0.00000	8.587.500	111.111
Prevotella	295.130.000	58.975.000	321.781.818	135.500.000	134.775.000	106.044.444
Alistipes	0.00000	0.85000	0.00000	2.490.909	100.000	0.00000
Rikenellaceae_RC9_gut_group	450.000	11.820.000	163.636	0.45455	225.000	611.111
Parabacteroides	2.155.000	11.190.000	2.081.818	6.027.273	32.787.500	33.111.111
Tannerella	0.00000	0.00000	0.00000	0.00000	162.500	0.00000
Desulfovibrio	0.00000	825.000	118.182	1.927.273	450.000	0.00000
Allobaculum	3.970.000	17.355.000	15.372.727	15.463.636	8.112.500	44.777.778
Holdemanella	1.790.000	1.890.000	3.427.273	4.081.818	6.287.500	39.077.778
Turicibacter	3.070.000	2.305.000	2.127.273	981.818	937.500	18.811.111
Enterococcus	0.20000	0.00000	100.000	0.00000	375.000	0.22222
Lactobacillus	0.00000	0.00000	181.818	200.000	3.512.500	122.222
Streptococcus	4.835.000	860.000	1.472.727	500.000	1.562.500	344.444
Johnsonella	0.00000	0.00000	0.00000	0.00000	212.500	0.00000
Lachnospiraceae_UCG-009	200.000	130.000	281.818	663.636	325.000	844.444
[Ruminococcus]_gauvreauii_group	605.000	505.000	2.700.000	1.072.727	337.500	855.556

Flavonifractor	2.745.000	3.850.000	936.364	1.609.091	762.500	1.700.000
Faecalibacterium	77.610.000	43.110.000	80.500.000	38.936.364	41.700.000	39.611.111
Negativibacillus	1.425.000	4.720.000	5.281.818	6.854.545	15.400.000	14.622.222
Family_XIII_AD3011_group	0.00000	0.00000	0.00000	0.00000	1.437.500	0.00000
Peptostreptococcus	0.00000	0.00000	0.00000	0.00000	512.500	0.00000
Romboutsia	6.120.000	1.110.000	5.818.182	4.700.000	3.800.000	38.400.000
Terrisporobacter	1.545.000	0.35000	427.273	154.545	400.000	0.00000
Finegoldia	0.00000	0.00000	0.00000	0.00000	662.500	0.00000
Acidaminococcus	0.00000	0.00000	0.00000	0.00000	500.000	0.00000
Phascolarctobacterium	35.620.000	42.215.000	54.772.727	45.054.545	37.062.500	49.155.556
Succinivibrio	2.570.000	5.040.000	3.409.091	1.072.727	2.700.000	1.333.333
Ralstonia	0.00000	0.00000	0.00000	0.00000	150.000	0.00000
Comamonas	0.10000	0.25000	0.18182	0.18182	812.500	0.88889
Escherichia-Shigella	9.825.000	5.640.000	32.981.818	29.927.273	77.300.000	3.266.667
Proteus	455.000	0.00000	0.00000	0.45455	3.250.000	0.44444
Marinomonas	0.00000	0.00000	0.00000	0.00000	137.500	0.00000
Pseudomonas	0.30000	335.000	0.00000	0.18182	1.375.000	177.778
Fretibacterium	0.00000	0.00000	0.00000	0.00000	137.500	0.00000
Bifidobacterium	0.00000	0.00000	0.00000	872.727	0.00000	122.222
Bilophila	0.40000	0.00000	0.00000	1.300.000	17.800.000	11.355.556
Candidatus_Stoquefichus	420.000	315.000	563.636	681.818	1.125.000	855.556
Catenibacterium	1.925.000	480.000	7.181.818	1.763.636	3.212.500	1.155.556
Cellulosilyticum	0.55000	0.00000	0.18182	0.00000	0.00000	0.55556
Oscillibacter	195.000	620.000	600.000	1.745.455	2.687.500	2.100.000
UCG-005	2.895.000	9.075.000	3.963.636	14.109.091	11.837.500	4.922.222
Peptococcus	800.000	980.000	490.909	1.300.000	112.500	1.211.111
[Eubacterium]_brachy_group	0.70000	0.60000	145.455	509.091	0.00000	0.55556
Candidatus_Arthromitus	170.000	0.50000	0.00000	0.90909	0.37500	1.088.889
Lachnospiraceae	0.00000	0.75000	0.00000	0.18182	0.00000	0.77778
Roseburia	1.445.000	1.875.000	5.218.182	4.190.909	1.737.500	7.233.333
Tuzzerella	530.000	825.000	16.509.091	436.364	0.25000	1.133.333
Colidextribacter	275.000	625.000	945.455	2.354.545	2.775.000	1.888.889
Prevotellaceae_Ga6A1_group	14.495.000	24.270.000	14.845.455	21.409.091	19.937.500	4.166.667
Mucispirillum	0.75000	0.35000	418.182	681.818	0.00000	344.444
Anaeroplasma	1.115.000	6.485.000	1.772.727	10.472.727	100.000	15.844.444
Erysipelotrichaceae_UCG-003	285.000	425.000	881.818	936.364	250.000	23.900.000
Fusicatenibacter	0.70000	0.00000	290.909	0.45455	0.00000	0.00000
Paeniclostridium	180.000	0.25000	0.00000	1.618.182	225.000	0.00000
Anaerobiospirillum	13.500.000	27.415.000	21.654.545	9.636.364	14.712.500	17.500.000
Lachnospira	2.135.000	0.60000	3.018.182	618.182	2.212.500	677.778
Allisonella	0.30000	0.00000	0.90909	0.00000	400.000	144.444
Megasphaera	1.655.000	0.00000	0.00000	0.00000	0.00000	0.00000
Phocea	0.70000	0.15000	0.00000	0.63636	400.000	211.111
Clostridioides	0.00000	0.90000	0.00000	0.00000	337.500	1.788.889
Sarcina	0.00000	0.00000	9.790.909	1.481.818	0.25000	0.00000
Gastranaerophilales	0.00000	3.680.000	0.00000	2.736.364	0.00000	1.755.556
Slackia	0.45000	0.85000	0.63636	163.636	0.75000	577.778
Parvibacter	0.00000	0.00000	0.27273	0.36364	0.00000	2.511.111
Paraprevotella	0.00000	480.000	0.00000	1.627.273	17.400.000	3.244.444
Mailhella	0.00000	0.00000	0.00000	0.54545	0.00000	0.55556
Harryflintia	0.00000	0.00000	0.00000	0.00000	0.00000	455.556

Incertae_Sedis	195.000	0.25000	0.72727	0.00000	0.50000	0.00000
[Eubacterium]_nodatum_group	0.00000	0.55000	200.000	163.636	0.00000	0.00000
GCA-900066575	0.00000	0.10000	0.45455	645.455	0.62500	0.44444
Anaerostignum	0.55000	0.50000	1.100.000	972.727	0.00000	211.111
Anaerofilum	225.000	0.45000	15.345.455	0.00000	0.00000	766.667
Holdemania	0.00000	0.15000	0.00000	0.00000	137.500	0.33333
[Eubacterium]_coprostanoligenes_group	0.00000	0.00000	100.000	0.00000	250.000	722.222
Vagococcus	0.00000	0.00000	0.00000	0.00000	162.500	0.00000
Epulopiscium	720.000	0.00000	22.927.273	6.645.455	0.00000	0.00000
Barnesiella	0.00000	180.000	0.00000	2.309.091	0.00000	0.00000
Leucobacter	0.45000	0.00000	0.00000	0.00000	0.00000	0.22222
Catenisphaera	0.00000	0.00000	0.00000	0.00000	0.00000	1.733.333
UBA1819	0.00000	0.00000	0.00000	0.27273	0.00000	322.222
Prevotellaceae_UCG-001	0.00000	0.00000	0.00000	190.909	0.00000	0.00000
Subdoligranulum	0.00000	0.00000	0.00000	281.818	0.00000	0.00000
Victivallis	0.00000	0.00000	0.00000	100.000	0.00000	0.00000
Akkermansia	0.00000	0.15000	0.00000	272.727	0.00000	0.00000
Dialister	0.00000	1.140.000	0.00000	0.00000	0.00000	0.00000
Christensenellaceae_R-7_group	0.00000	0.00000	245.455	0.00000	0.00000	0.00000
