# Supplementary material for: Analysis of Intestinal Microbiota and Metabolic Pathways before and after a 2-Month-Long Hydrolyzed Fish and Rice Starch Hypoallergenic Diet Trial in Pruritic Dogs
Source: Vet Sci. 2023 Jul 21;10(7):478. doi: 10.3390/vetsci10070478 (PMC10384699; doi:10.3390/vetsci10070478)
Supplement: Supplementary file 1 [file vetsci-10-00478-s001.zip › Table S3.pdf]

Table S3: OTU percent abundance of bacterial species in pre- and post-diet fecal samples. AFR = adverse food reaction; D = doubtful diagnosis; CAD = canine atopic dermatitis.

**Percentage mean bacterial species**	**Pre-AFR**	**Post-AFR**	**Pre-D**	**Post-D**	**Pre-CAD**	**Post-CAD**
Fusobacterium	30,3	31,6	18,4	23,4	21,3	24,4
Bacteroides	19,6	23,7	19,5	25,8	34,7	20,9
Prevotella	18,5	3,7	14,7	6,5	6,1	4,9
Alloprevotella	5,0	9,8	7,8	10,0	3,8	7,9
Faecalibacterium	4,9	2,7	3,7	1,9	1,9	1,8
Sutterella	3,7	3,7	4,8	6,6	3,7	3,8
Megamonas	2,9	3,7	4,4	4,2	7,5	3,2
Phascolarctobacterium	2,2	2,6	2,5	2,2	1,7	2,3
Blautia	1,6	1,3	2,2	1,5	0,9	2,8
uncultured	1,2	1,7	3,8	2,0	0,8	1,8
Parasutterella	1,1	0,7	0,7	0,7	0,3	0,5
Prevotellaceae_Ga6A1_group	0,9	1,5	0,7	1,0	0,9	0,2
Anaerobiospirillum	0,8	1,7	1,0	0,5	0,7	0,8
Escherichia-Shigella	0,6	0,4	1,5	1,4	3,5	0,2
Peptoclostridium	0,5	1,4	0,8	1,1	0,7	1,3
Helicobacter	0,5	1,4	1,8	0,4	0,8	0,5
Romboutsia	0,4	0,1	0,3	0,2	0,2	1,8
[Ruminococcus]_gnavus_group	0,4	0,2	0,6	0,2	0,6	0,2
Lachnospiraceae_NK4A136_group	0,4	0,4	0,7	0,9	0,5	0,3
Clostridium_sensu_stricto_1	0,4	0,1	0,8	2,4	0,6	0,8
Faecalitalea	0,3	0,2	1,0	0,3	0,2	0,4
Streptococcus	0,3	0,1	0,1	0,0	0,1	0,0
Collinsella	0,3	0,3	0,2	0,2	0,2	4,3
Allobaculum	0,2	1,1	0,7	0,7	0,4	2,1
Campylobacter	0,2	0,4	0,5	0,8	0,3	0,8
Turicibacter	0,2	0,1	0,1	0,0	0,0	0,9
Erysipelatoclostridium	0,2	0,2	0,4	0,2	0,1	0,9
UCG-005	0,2	0,6	0,2	0,7	0,5	0,2
Flavonifractor	0,2	0,2	0,0	0,1	0,0	0,1
Succinivibrio	0,2	0,3	0,2	0,1	0,1	0,1
Parabacteroides	0,1	0,7	0,1	0,3	1,5	1,5
Lachnospira	0,1	0,0	0,1	0,0	0,1	0,0
Catenibacterium	0,1	0,0	0,3	0,1	0,1	0,1
Lachnoclostridium	0,1	0,1	0,3	0,2	0,3	0,4
Holdemanella	0,1	0,1	0,2	0,2	0,3	1,8
Megasphaera	0,1	0,0	0,0	0,0	0,0	0,0
Terrisporobacter	0,1	0,0	0,0	0,0	0,0	0,0
Roseburia	0,1	0,1	0,2	0,2	0,1	0,3
Negativibacillus	0,1	0,3	0,2	0,3	0,7	0,7
Clostridia_UCG-014	0,1	0,2	0,8	0,2	0,3	0,8
Anaeroplasma	0,1	0,4	0,1	0,5	0,0	0,7
[Ruminococcus]_torques_group	0,1	0,1	0,0	0,1	0,0	0,2
Butyricicoccus	0,1	0,1	0,2	0,1	0,0	0,1
Tyzzerella	0,1	0,1	0,1	0,0	0,0	0,1
Peptococcus	0,1	0,1	0,0	0,1	0,0	0,1
Epulopiscium	0,0	0,0	1,0	0,3	0,0	0,0
[Ruminococcus]_gauvreauii_group	0,0	0,0	0,1	0,1	0,0	0,0

Tuzzerella	0,0	0,1	0,8	0,0	0,0	0,1
Fournierella	0,0	0,0	0,0	0,0	0,0	0,0
Muribaculaceae	0,0	0,1	0,1	0,1	0,3	0,1
Proteus	0,0	0,0	0,0	0,0	0,1	0,0
Rikenellaceae_RC9_gut_group	0,0	0,7	0,0	0,0	0,0	0,0
Candidatus_Stoquefichus	0,0	0,0	0,0	0,0	0,1	0,0
Intestinimonas	0,0	0,0	0,0	0,0	0,0	0,0
Sellimonas	0,0	0,0	0,0	0,0	0,0	0,0
Erysipelotrichaceae_UCG-003	0,0	0,0	0,0	0,0	0,0	1,1
Colidextribacter	0,0	0,0	0,0	0,1	0,1	0,1
Anaerofilum	0,0	0,0	0,7	0,0	0,0	0,0
Lachnospiraceae_UCG-009	0,0	0,0	0,0	0,0	0,0	0,0
Oscillibacter	0,0	0,0	0,0	0,1	0,1	0,1
Incertae_Sedis	0,0	0,0	0,0	0,0	0,0	0,0
Candidatus_Arthromitus	0,0	0,0	0,0	0,0	0,0	0,1
Paeniclostridium	0,0	0,0	0,0	0,1	0,0	0,0
Mucispirillum	0,0	0,0	0,0	0,0	0,0	0,0
[Eubacterium]_brachy_group	0,0	0,0	0,0	0,0	0,0	0,0
Fusicatenibacter	0,0	0,0	0,0	0,0	0,0	0,0
Phocea	0,0	0,0	0,0	0,0	0,0	0,0
Bilophila	0,0	0,0	0,0	0,1	0,8	0,5
Cellulosilyticum	0,0	0,0	0,0	0,0	0,0	0,0
Slackia	0,0	0,0	0,0	0,0	0,0	0,0
Anaerostignum	0,0	0,0	0,1	0,0	0,0	0,0
Leucobacter	0,0	0,0	0,0	0,0	0,0	0,0
Pseudomonas	0,0	0,0	0,0	0,0	0,1	0,0
Allisonella	0,0	0,0	0,0	0,0	0,0	0,0
Enterococcus	0,0	0,0	0,0	0,0	0,0	0,0
Comamonas	0,0	0,0	0,0	0,0	0,0	0,0
Actinomyces	0,0	0,0	0,0	0,0	0,0	0,0
Trueperella	0,0	0,0	0,0	0,0	0,0	0,0
Corynebacterium	0,0	0,0	0,0	0,0	0,2	0,0
Lawsonella	0,0	0,0	0,0	0,0	0,0	0,0
Odoribacter	0,0	0,0	0,0	0,0	0,0	0,0
Porphyromonas	0,0	0,0	0,0	0,0	0,4	0,0
Alistipes	0,0	0,0	0,0	0,1	0,0	0,0
Tannerella	0,0	0,0	0,0	0,0	0,0	0,0
Desulfovibrio	0,0	0,1	0,0	0,1	0,0	0,0
Lactobacillus	0,0	0,0	0,0	0,0	0,2	0,0
Johnsonella	0,0	0,0	0,0	0,0	0,0	0,0
Family_XIII_AD3011_group	0,0	0,0	0,0	0,0	0,1	0,0
Peptostreptococcus	0,0	0,0	0,0	0,0	0,0	0,0
Finegoldia	0,0	0,0	0,0	0,0	0,0	0,0
Acidaminococcus	0,0	0,0	0,0	0,0	0,0	0,0
Ralstonia	0,0	0,0	0,0	0,0	0,0	0,0
Marinomonas	0,0	0,0	0,0	0,0	0,0	0,0
Fretibacterium	0,0	0,0	0,0	0,0	0,0	0,0
Bifidobacterium	0,0	0,0	0,0	0,0	0,0	0,0
Lachnospiraceae	0,0	0,0	0,0	0,0	0,0	0,0
Clostridioides	0,0	0,0	0,0	0,0	0,0	0,1
Sarcina	0,0	0,0	0,4	0,1	0,0	0,0

Gastranaerophilales	0,0	0,2	0,0	0,1	0,0	0,1
Parvibacter	0,0	0,0	0,0	0,0	0,0	0,1
Paraprevotella	0,0	0,0	0,0	0,1	0,8	0,2
Mailhella	0,0	0,0	0,0	0,0	0,0	0,0
Harryflintia	0,0	0,0	0,0	0,0	0,0	0,0
[Eubacterium]_nodatum_group	0,0	0,0	0,0	0,0	0,0	0,0
GCA-900066575	0,0	0,0	0,0	0,0	0,0	0,0
Holdemania	0,0	0,0	0,0	0,0	0,0	0,0
Eubacterium]_coprostanoligenes_group	0,0	0,0	0,0	0,0	0,0	0,0
Vagococcus	0,0	0,0	0,0	0,0	0,0	0,0
Barnesiella	0,0	0,0	0,0	0,1	0,0	0,0
Catenisphaera	0,0	0,0	0,0	0,0	0,0	0,1
UBA1819	0,0	0,0	0,0	0,0	0,0	0,0
Prevotellaceae_UCG-001	0,0	0,0	0,0	0,0	0,0	0,0
Subdoligranulum	0,0	0,0	0,0	0,0	0,0	0,0
Victivallis	0,0	0,0	0,0	0,0	0,0	0,0
Akkermansia	0,0	0,0	0,0	0,0	0,0	0,0
Dialister	0,0	0,1	0,0	0,0	0,0	0,0
Christensenellaceae_R-7_group	0,0	0,0	0,0	0,0	0,0	0,0
