# Supplementary material for: Analysis of Intestinal Microbiota and Metabolic Pathways before and after a 2-Month-Long Hydrolyzed Fish and Rice Starch Hypoallergenic Diet Trial in Pruritic Dogs
Source: Vet Sci. 2023 Jul 21;10(7):478. doi: 10.3390/vetsci10070478 (PMC10384699; doi:10.3390/vetsci10070478)
Supplement: Supplementary file 1 [file vetsci-10-00478-s001.zip › Table S4.pdf]

Bacteria
d_Bacteria.p_Firmicutes.c_Bacilli.o_Erysipelotrichales.f_Erysipelatoclostridiaceae.g_Erysipelatoclostridium
d_Bacteria.p_Firmicutes.c_Clostridia.o_Clostridia_UCG_014.f_Clostridia_UCG_014.g_Clostridia_UCG_014
d_Bacteria.p_Bacteroidota.c_Bacteroidia.o_Bacteroidales.f_Marinifilaceae.g_Odoribacter
d_Bacteria.p_Bacteroidota.c_Bacteroidia.o_Bacteroidales.f_Tannerellaceae.g_Parabacteroides
d_Bacteria.p_Firmicutes.c_Clostridia.o_Oscillospirales.f_Ruminococcaceae.g_Negativibacillus
d_Bacteria.p_Firmicutes.c_Clostridia.o_Lachnospirales.f_Lachnospiraceae.g_Roseburia
d_Bacteria.p_Firmicutes.c_Clostridia.o_Oscillospirales.f_Oscillospiraceae.g_Colidextribacter
d_Bacteria.p_Cyanobacteria.c_Vampirivibrionia.o_Gastranaerophilales.f_Gastranaerophilales.g_Gastranaerophilale
d_Bacteria.p_Bacteroidota.c_Bacteroidia.o_Bacteroidales.f_Prevotellaceae.g_Paraprevotella
d_Bacteria.p_Firmicutes.c_Bacilli
d_Bacteria.p_Firmicutes.c_Bacilli.o_Erysipelotrichales
d_Bacteria.p_Firmicutes.c_Bacilli.o_Erysipelotrichales.f_Erysipelotrichaceae
d_Bacteria.p_Firmicutes.c_Clostridia.o_Clostridia_UCG_014
d_Bacteria.p_Firmicutes.c_Clostridia.o_Clostridia_UCG_014.f_Clostridia_UCG_014
d_Bacteria.p_Firmicutes.c_Clostridia.o_Oscillospirales.f_Oscillospiraceae
d_Bacteria.p_Bacteroidota.c_Bacteroidia.o_Bacteroidales.f_Marinifilaceae
d_Bacteria.p_Bacteroidota.c_Bacteroidia.o_Bacteroidales.f_Tannerellaceae
d_Bacteria.p_Cyanobacteria
d_Bacteria.p_Cyanobacteria.c_Vampirivibrionia
d_Bacteria.p_Cyanobacteria.c_Vampirivibrionia.o_Gastranaerophilales
d_Bacteria.p_Cyanobacteria.c_Vampirivibrionia.o_Gastranaerophilales.f_Gastranaerophilales
d_Bacteria.p_Bacteroidota.c_Bacteroidia.o_Bacteroidales.f_Prevotellaceae.g_Prevotella
d_Bacteria.p_Firmicutes.c_Clostridia.o_Peptostreptococcales_Tissierellales.f_Peptostreptococcaceae.g_Terrisporob
d_Bacteria.p_Firmicutes.c_Clostridia.o_Lachnospirales.f_Lachnospiraceae.g_Lachnospira
d_Bacteria.p_Firmicutes.c_Clostridia.o_Lachnospirales.f_Lachnospiraceae.g_Epulopiscium
d_Bacteria.p_Actinobacteriota.c_Coriobacteriia.o_Coriobacteriales.f_Coriobacteriaceae.g_Collinsella
d_Bacteria.p_Bacteroidota.c_Bacteroidia.o_Bacteroidales.f_Bacteroidaceae.g_Bacteroides
d_Bacteria.p_Bacteroidota.c_Bacteroidia.o_Bacteroidales.f_Muribaculaceae.g_Muribaculaceae
d_Bacteria.p_Bacteroidota.c_Bacteroidia.o_Bacteroidales.f_Prevotellaceae.g_Alloprevotella
d_Bacteria.p_Campilobacterota.c_Campylobacteria.o_Campylobacterales.f_Campylobacteraceae.g_Campylobacter
d_Bacteria.p_Campilobacterota.c_Campylobacteria.o_Campylobacterales.f_Helicobacteraceae.g_Helicobacter
d_Bacteria.p_Firmicutes.c_Bacilli.o_Erysipelotrichales.f_Erysipelotrichaceae.g_Faecalitalea
d_Bacteria.p_Firmicutes.c_Bacilli.o_Erysipelotrichales.f_Erysipelotrichaceae.g_uncultured
d_Bacteria.p_Firmicutes.c_Clostridia.o_Clostridiales.f_Clostridiaceae.g_Clostridium_sensu_stricto_1
d_Bacteria.p_Firmicutes.c_Clostridia.o_Lachnospirales.f_Lachnospiraceae._
d_Bacteria.p_Firmicutes.c_Clostridia.o_Lachnospirales.f_Lachnospiraceae.g_Blautia
d_Bacteria.p_Firmicutes.c_Clostridia.o_Lachnospirales.f_Lachnospiraceae.g_Lachnoclostridium
d_Bacteria.p_Firmicutes.c_Clostridia.o_Lachnospirales.f_Lachnospiraceae.g_Lachnospiraceae_NK4A136_group
d_Bacteria.p_Firmicutes.c_Clostridia.o_Lachnospirales.f_Lachnospiraceae.g_Sellimonas
d_Bacteria.p_Firmicutes.c_Clostridia.o_Lachnospirales.f_Lachnospiraceae.g_Tyzzerella
d_Bacteria.p_Firmicutes.c_Clostridia.o_Lachnospirales.f_Lachnospiraceae.g__Ruminococcus__gnavus_group
d_Bacteria.p_Firmicutes.c_Clostridia.o_Lachnospirales.f_Lachnospiraceae.g__Ruminococcus__torques_group
d_Bacteria.p_Firmicutes.c_Clostridia.o_Oscillospirales.f_Butyricicoccaceae.g_Butyricicoccus
d_Bacteria.p_Firmicutes.c_Clostridia.o_Oscillospirales.f_Oscillospiraceae.g_Intestinimonas
d_Bacteria.p_Firmicutes.c_Clostridia.o_Oscillospirales.f_Ruminococcaceae._
d_Bacteria.p_Firmicutes.c_Clostridia.o_Oscillospirales.f_Ruminococcaceae.g_Fournierella

d_Bacteria.p_Firmicutes.c_Clostridia.o_Peptostreptococcales_Tissierellales.f_Peptostreptococcaceae.g_Peptoclostr
d_Bacteria.p_Firmicutes.c_Negativicutes.o_Veillonellales_Selenomonadales.f_Selenomonadaceae.g_Megamonas
d_Bacteria.p_Fusobacteriota.c_Fusobacteriia.o_Fusobacteriales.f_Fusobacteriaceae.g_Fusobacterium
d_Bacteria.p_Proteobacteria.c_Gammaproteobacteria.o_Burkholderiales.f_Sutterellaceae.g_Parasutterella
d_Bacteria.p_Proteobacteria.c_Gammaproteobacteria.o_Burkholderiales.f_Sutterellaceae.g_Sutterella
d_Bacteria.p_Actinobacteriota.c_Actinobacteria.o_Actinomycetales.f_Actinomycetaceae.g_Actinomyces
d_Bacteria.p_Actinobacteriota.c_Actinobacteria.o_Actinomycetales.f_Actinomycetaceae.g_Trueperella
d_Bacteria.p_Actinobacteriota.c_Actinobacteria.o_Corynebacteriales.f_Corynebacteriaceae.g_Corynebacterium
d_Bacteria.p_Actinobacteriota.c_Actinobacteria.o_Corynebacteriales.f_Corynebacteriaceae.g_Lawsonella
d_Bacteria.p_Bacteroidota.c_Bacteroidia.o_Bacteroidales.f_Porphyromonadaceae.g_Porphyromonas
d_Bacteria.p_Bacteroidota.c_Bacteroidia.o_Bacteroidales.f_Rikenellaceae.g_Alistipes
d_Bacteria.p_Bacteroidota.c_Bacteroidia.o_Bacteroidales.f_Rikenellaceae.g_Rikenellaceae_RC9_gut_group
d_Bacteria.p_Bacteroidota.c_Bacteroidia.o_Bacteroidales.f_Tannerellaceae.g_Tannerella
d_Bacteria.p_Desulfobacterota.c_Desulfovibrionia.o_Desulfovibrionales.f_Desulfovibrionaceae.g_Desulfovibrio
d_Bacteria.p_Firmicutes.c_Bacilli.o_Erysipelotrichales.f_Erysipelotrichaceae.g_Allobaculum
d_Bacteria.p_Firmicutes.c_Bacilli.o_Erysipelotrichales.f_Erysipelotrichaceae.g_Holdemanella
d_Bacteria.p_Firmicutes.c_Bacilli.o_Erysipelotrichales.f_Erysipelotrichaceae.g_Turicibacter
d_Bacteria.p_Firmicutes.c_Bacilli.o_Lactobacillales.f_Enterococcaceae.g_Enterococcus
d_Bacteria.p_Firmicutes.c_Bacilli.o_Lactobacillales.f_Lactobacillaceae.g_Lactobacillus
d_Bacteria.p_Firmicutes.c_Bacilli.o_Lactobacillales.f_Streptococcaceae.g_Streptococcus
d_Bacteria.p_Firmicutes.c_Clostridia.o_Lachnospirales.f_Lachnospiraceae.g_Johnsonella
d_Bacteria.p_Firmicutes.c_Clostridia.o_Lachnospirales.f_Lachnospiraceae.g_Lachnospiraceae_UCG_009
d_Bacteria.p_Firmicutes.c_Clostridia.o_Lachnospirales.f_Lachnospiraceae.g__Ruminococcus__gauvreauii_group
d_Bacteria.p_Firmicutes.c_Clostridia.o_Oscillospirales.f_Butyricicoccaceae._
d_Bacteria.p_Firmicutes.c_Clostridia.o_Oscillospirales.f_Oscillospiraceae.g_Flavonifractor
d_Bacteria.p_Firmicutes.c_Clostridia.o_Oscillospirales.f_Ruminococcaceae.g_Faecalibacterium
d_Bacteria.p_Firmicutes.c_Clostridia.o_Peptostreptococcales_Tissierellales.f_Anaerovoracaceae.g_Family_XIII_AD3
d_Bacteria.p_Firmicutes.c_Clostridia.o_Peptostreptococcales_Tissierellales.f_Peptostreptococcaceae.g_Peptostrep
d_Bacteria.p_Firmicutes.c_Clostridia.o_Peptostreptococcales_Tissierellales.f_Peptostreptococcaceae.g_Romboutsia
d_Bacteria.p_Firmicutes.c_Clostridia.o_Peptostreptococcales_Tissierellales.f_Peptostreptococcales_Tissierellales.g_
d_Bacteria.p_Firmicutes.c_Negativicutes.o_Acidaminococcales.f_Acidaminococcaceae.g_Acidaminococcus
d_Bacteria.p_Firmicutes.c_Negativicutes.o_Acidaminococcales.f_Acidaminococcaceae.g_Phascolarctobacterium
d_Bacteria.p_Proteobacteria.c_Gammaproteobacteria.o_Aeromonadales.f_Succinivibrionaceae.g_Succinivibrio
d_Bacteria.p_Proteobacteria.c_Gammaproteobacteria.o_Burkholderiales.f_Burkholderiaceae.g_Ralstonia
d_Bacteria.p_Proteobacteria.c_Gammaproteobacteria.o_Burkholderiales.f_Comamonadaceae._
d_Bacteria.p_Proteobacteria.c_Gammaproteobacteria.o_Burkholderiales.f_Comamonadaceae.g_Comamonas
d_Bacteria.p_Proteobacteria.c_Gammaproteobacteria.o_Enterobacterales.f_Enterobacteriaceae.g_Escherichia_Shi
d_Bacteria.p_Proteobacteria.c_Gammaproteobacteria.o_Enterobacterales.f_Morganellaceae.g_Proteus
d_Bacteria.p_Proteobacteria.c_Gammaproteobacteria.o_Oceanospirillales.f_Marinomonadaceae.g_Marinomonas
d_Bacteria.p_Proteobacteria.c_Gammaproteobacteria.o_Pseudomonadales.f_Pseudomonadaceae.g_Pseudomonas
d_Bacteria.p_Synergistota.c_Synergistia.o_Synergistales.f_Synergistaceae.g_Fretibacterium
d_Bacteria.p_Actinobacteriota.c_Actinobacteria.o_Bifidobacteriales.f_Bifidobacteriaceae.g_Bifidobacterium
d_Bacteria.p_Desulfobacterota.c_Desulfovibrionia.o_Desulfovibrionales.f_Desulfovibrionaceae.g_Bilophila
d_Bacteria.p_Firmicutes.c_Bacilli.o_Erysipelotrichales.f_Erysipelatoclostridiaceae.g_Candidatus_Stoquefichus
d_Bacteria.p_Firmicutes.c_Bacilli.o_Erysipelotrichales.f_Erysipelatoclostridiaceae.g_Catenibacterium
d_Bacteria.p_Firmicutes.c_Clostridia.o_Lachnospirales.f_Lachnospiraceae.g_Cellulosilyticum
d_Bacteria.p_Firmicutes.c_Clostridia.o_Oscillospirales.f_Oscillospiraceae.g_Oscillibacter

d_Bacteria.p_Firmicutes.c_Clostridia.o_Oscillospirales.f_Oscillospiraceae.g_UCG_005
d_Bacteria.p_Firmicutes.c_Clostridia.o_Peptococcales.f_Peptococcaceae.g_Peptococcus
d_Bacteria.p_Firmicutes.c_Clostridia.o_Peptostreptococcales_Tissierellales.f_Anaerovoracaceae.g__Eubacterium__
d_Bacteria.p_Firmicutes.c_Clostridia.o_Clostridiales.f_Clostridiaceae.g_Candidatus_Arthromitus
d_Bacteria.p_Firmicutes.c_Clostridia.o_Lachnospirales.f_Lachnospiraceae.g_Lachnospiraceae
d_Bacteria.p_Firmicutes.c_Clostridia.o_Lachnospirales.f_Lachnospiraceae.g_Tuzzerella
d_Bacteria.p_Firmicutes.c_Clostridia.o_Oscillospirales.f_Oscillospiraceae._
d_Bacteria.p_Bacteroidota.c_Bacteroidia.o_Bacteroidales.f_Prevotellaceae.g_Prevotellaceae_Ga6A1_group
d_Bacteria.p_Deferribacterota.c_Deferribacteres.o_Deferribacterales.f_Deferribacteraceae.g_Mucispirillum
d_Bacteria.p_Firmicutes.c_Bacilli.o_Acholeplasmatales.f_Acholeplasmataceae.g_Anaeroplasma
d_Bacteria.p_Firmicutes.c_Bacilli.o_Erysipelotrichales.f_Erysipelatoclostridiaceae.g_Erysipelotrichaceae_UCG_003
d_Bacteria.p_Firmicutes.c_Clostridia.o_Lachnospirales.f_Lachnospiraceae.g_Fusicatenibacter
d_Bacteria.p_Firmicutes.c_Clostridia.o_Peptostreptococcales_Tissierellales.f_Peptostreptococcaceae.g_Paeniclostr
d_Bacteria.p_Proteobacteria.c_Gammaproteobacteria.o_Aeromonadales.f_Succinivibrionaceae.g_Anaerobiospirillu
d_Bacteria.p_Firmicutes.c_Negativicutes.o_Veillonellales_Selenomonadales.f_Veillonellaceae.g_Allisonella
d_Bacteria.p_Firmicutes.c_Negativicutes.o_Veillonellales_Selenomonadales.f_Veillonellaceae.g_Megasphaera
d_Bacteria.p_Firmicutes.c_Clostridia.o_Oscillospirales.f_Ruminococcaceae.g_Phocea
d_Bacteria.p_Firmicutes.c_Clostridia.o_Peptostreptococcales_Tissierellales.f_Peptostreptococcaceae.g_Clostridioid
d_Bacteria.p_Firmicutes.c_Clostridia.o_Clostridiales.f_Clostridiaceae.g_Sarcina
d_Bacteria.p_Actinobacteriota.c_Coriobacteriia.o_Coriobacteriales.f_Eggerthellaceae.g_Slackia
d_Bacteria.p_Actinobacteriota.c_Coriobacteriia.o_Coriobacteriales.f_Eggerthellaceae.g_Parvibacter
d_Bacteria.p_Desulfobacterota.c_Desulfovibrionia.o_Desulfovibrionales.f_Desulfovibrionaceae.g_Mailhella
d_Bacteria.p_Firmicutes.c_Clostridia.o_Oscillospirales.f_Ruminococcaceae.g_Harryflintia
d_Bacteria.p_Firmicutes.c_Clostridia.o_Oscillospirales.f_Ruminococcaceae.g_Incertae_Sedis
d_Bacteria.p_Proteobacteria.c_Gammaproteobacteria.o_Enterobacterales.f_Enterobacteriaceae._
d_Bacteria.p_Firmicutes.c_Clostridia.o_Peptostreptococcales_Tissierellales.f_Anaerovoracaceae.g__Eubacterium__
d_Bacteria.p_Firmicutes.c_Clostridia.o_Lachnospirales.f_Lachnospiraceae.g_GCA_900066575
d_Bacteria.p_Firmicutes.c_Clostridia.o_Lachnospirales.f_Lachnospiraceae.g_Anaerostignum
d_Bacteria.p_Firmicutes.c_Clostridia.o_Oscillospirales.f_Ruminococcaceae.g_Anaerofilum
d_Bacteria.p_Firmicutes.c_Bacilli.o_Erysipelotrichales.f_Erysipelotrichaceae.g_Holdemania
d_Bacteria.p_Firmicutes.c_Clostridia.o_Oscillospirales.f__Eubacterium__coprostanoligenes_group.g__Eubacterium_
d_Bacteria.p_Firmicutes.c_Bacilli.o_Lactobacillales.f_Vagococcaceae.g_Vagococcus
d_Bacteria.p_Bacteroidota.c_Bacteroidia.o_Bacteroidales.f_Barnesiellaceae.g_Barnesiella
d_Bacteria.p_Actinobacteriota.c_Actinobacteria.o_Micrococcales.f_Microbacteriaceae.g_Leucobacter
d_Bacteria.p_Firmicutes.c_Bacilli.o_Erysipelotrichales.f_Erysipelotrichaceae.g_Catenisphaera
d_Bacteria.p_Firmicutes.c_Clostridia.o_Oscillospirales.f_Ruminococcaceae.g_UBA1819
d_Bacteria.p_Bacteroidota.c_Bacteroidia.o_Bacteroidales.f_Prevotellaceae.g_Prevotellaceae_UCG_001
d_Bacteria.p_Firmicutes.c_Clostridia.o_Oscillospirales.f_Ruminococcaceae.g_Subdoligranulum
d_Bacteria.p_Verrucomicrobiota.c_Lentisphaeria.o_Victivallales.f_Victivallaceae.g_Victivallis
d_Bacteria.p_Verrucomicrobiota.c_Verrucomicrobiae.o_Verrucomicrobiales.f_Akkermansiaceae.g_Akkermansia
d_Bacteria.p_Firmicutes.c_Negativicutes.o_Veillonellales_Selenomonadales.f_Veillonellaceae.g_Dialister
d_Bacteria.p_Firmicutes.c_Clostridia.o_Christensenellales.f_Christensenellaceae.g_Christensenellaceae_R_7_group
d_Bacteria
d_Bacteria.p_Actinobacteriota
d_Bacteria.p_Actinobacteriota.c_Coriobacteriia
d_Bacteria.p_Actinobacteriota.c_Coriobacteriia.o_Coriobacteriales
d_Bacteria.p_Actinobacteriota.c_Coriobacteriia.o_Coriobacteriales.f_Coriobacteriaceae

d_Bacteria.p_Bacteroidota
d_Bacteria.p_Bacteroidota.c_Bacteroidia
d_Bacteria.p_Bacteroidota.c_Bacteroidia.o_Bacteroidales
d_Bacteria.p_Bacteroidota.c_Bacteroidia.o_Bacteroidales.f_Bacteroidaceae
d_Bacteria.p_Bacteroidota.c_Bacteroidia.o_Bacteroidales.f_Muribaculaceae
d_Bacteria.p_Bacteroidota.c_Bacteroidia.o_Bacteroidales.f_Prevotellaceae
d_Bacteria.p_Campilobacterota
d_Bacteria.p_Campilobacterota.c_Campylobacteria
d_Bacteria.p_Campilobacterota.c_Campylobacteria.o_Campylobacterales
d_Bacteria.p_Campilobacterota.c_Campylobacteria.o_Campylobacterales.f_Campylobacteraceae
d_Bacteria.p_Campilobacterota.c_Campylobacteria.o_Campylobacterales.f_Helicobacteraceae
d_Bacteria.p_Firmicutes
d_Bacteria.p_Firmicutes.c_Bacilli.o_Erysipelotrichales.f_Erysipelatoclostridiaceae
d_Bacteria.p_Firmicutes.c_Clostridia
d_Bacteria.p_Firmicutes.c_Clostridia.o_Clostridiales
d_Bacteria.p_Firmicutes.c_Clostridia.o_Clostridiales.f_Clostridiaceae
d_Bacteria.p_Firmicutes.c_Clostridia.o_Lachnospirales
d_Bacteria.p_Firmicutes.c_Clostridia.o_Lachnospirales.f_Lachnospiraceae
d_Bacteria.p_Firmicutes.c_Clostridia.o_Oscillospirales
d_Bacteria.p_Firmicutes.c_Clostridia.o_Oscillospirales.f_Butyricicoccaceae
d_Bacteria.p_Firmicutes.c_Clostridia.o_Oscillospirales.f_Ruminococcaceae
d_Bacteria.p_Firmicutes.c_Clostridia.o_Peptostreptococcales_Tissierellales
d_Bacteria.p_Firmicutes.c_Clostridia.o_Peptostreptococcales_Tissierellales.f_Peptostreptococcaceae
d_Bacteria.p_Firmicutes.c_Negativicutes
d_Bacteria.p_Firmicutes.c_Negativicutes.o_Veillonellales_Selenomonadales
d_Bacteria.p_Firmicutes.c_Negativicutes.o_Veillonellales_Selenomonadales.f_Selenomonadaceae
d_Bacteria.p_Fusobacteriota
d_Bacteria.p_Fusobacteriota.c_Fusobacteriia
d_Bacteria.p_Fusobacteriota.c_Fusobacteriia.o_Fusobacteriales
d_Bacteria.p_Fusobacteriota.c_Fusobacteriia.o_Fusobacteriales.f_Fusobacteriaceae
d_Bacteria.p_Proteobacteria
d_Bacteria.p_Proteobacteria.c_Gammaproteobacteria
d_Bacteria.p_Proteobacteria.c_Gammaproteobacteria.o_Burkholderiales
d_Bacteria.p_Proteobacteria.c_Gammaproteobacteria.o_Burkholderiales.f_Sutterellaceae
d_Bacteria.p_Actinobacteriota.c_Actinobacteria
d_Bacteria.p_Actinobacteriota.c_Actinobacteria.o_Actinomycetales
d_Bacteria.p_Actinobacteriota.c_Actinobacteria.o_Actinomycetales.f_Actinomycetaceae
d_Bacteria.p_Actinobacteriota.c_Actinobacteria.o_Corynebacteriales
d_Bacteria.p_Actinobacteriota.c_Actinobacteria.o_Corynebacteriales.f_Corynebacteriaceae
d_Bacteria.p_Bacteroidota.c_Bacteroidia.o_Bacteroidales.f_Porphyromonadaceae
d_Bacteria.p_Bacteroidota.c_Bacteroidia.o_Bacteroidales.f_Rikenellaceae
d_Bacteria.p_Desulfobacterota
d_Bacteria.p_Desulfobacterota.c_Desulfovibrionia
d_Bacteria.p_Desulfobacterota.c_Desulfovibrionia.o_Desulfovibrionales
d_Bacteria.p_Desulfobacterota.c_Desulfovibrionia.o_Desulfovibrionales.f_Desulfovibrionaceae
d_Bacteria.p_Firmicutes.c_Bacilli.o_Lactobacillales
d_Bacteria.p_Firmicutes.c_Bacilli.o_Lactobacillales.f_Enterococcaceae

d_Bacteria.p_Firmicutes.c_Bacilli.o_Lactobacillales.f_Lactobacillaceae
d_Bacteria.p_Firmicutes.c_Bacilli.o_Lactobacillales.f_Streptococcaceae
d_Bacteria.p_Firmicutes.c_Clostridia.o_Peptostreptococcales_Tissierellales.f_Anaerovoracaceae
d_Bacteria.p_Firmicutes.c_Clostridia.o_Peptostreptococcales_Tissierellales.f_Peptostreptococcales_Tissierellales
d_Bacteria.p_Firmicutes.c_Negativicutes.o_Acidaminococcales
d_Bacteria.p_Firmicutes.c_Negativicutes.o_Acidaminococcales.f_Acidaminococcaceae
d_Bacteria.p_Proteobacteria.c_Gammaproteobacteria.o_Aeromonadales
d_Bacteria.p_Proteobacteria.c_Gammaproteobacteria.o_Aeromonadales.f_Succinivibrionaceae
d_Bacteria.p_Proteobacteria.c_Gammaproteobacteria.o_Burkholderiales.f_Burkholderiaceae
d_Bacteria.p_Proteobacteria.c_Gammaproteobacteria.o_Burkholderiales.f_Comamonadaceae
d_Bacteria.p_Proteobacteria.c_Gammaproteobacteria.o_Enterobacterales
d_Bacteria.p_Proteobacteria.c_Gammaproteobacteria.o_Enterobacterales.f_Enterobacteriaceae
d_Bacteria.p_Proteobacteria.c_Gammaproteobacteria.o_Enterobacterales.f_Morganellaceae
d_Bacteria.p_Proteobacteria.c_Gammaproteobacteria.o_Oceanospirillales
d_Bacteria.p_Proteobacteria.c_Gammaproteobacteria.o_Oceanospirillales.f_Marinomonadaceae
d_Bacteria.p_Proteobacteria.c_Gammaproteobacteria.o_Pseudomonadales
d_Bacteria.p_Proteobacteria.c_Gammaproteobacteria.o_Pseudomonadales.f_Pseudomonadaceae
d_Bacteria.p_Synergistota
d_Bacteria.p_Synergistota.c_Synergistia
d_Bacteria.p_Synergistota.c_Synergistia.o_Synergistales
d_Bacteria.p_Synergistota.c_Synergistia.o_Synergistales.f_Synergistaceae
d_Bacteria.p_Actinobacteriota.c_Actinobacteria.o_Bifidobacteriales
d_Bacteria.p_Actinobacteriota.c_Actinobacteria.o_Bifidobacteriales.f_Bifidobacteriaceae
d_Bacteria.p_Firmicutes.c_Clostridia.o_Peptococcales
d_Bacteria.p_Firmicutes.c_Clostridia.o_Peptococcales.f_Peptococcaceae
d_Bacteria.p_Deferribacterota
d_Bacteria.p_Deferribacterota.c_Deferribacteres
d_Bacteria.p_Deferribacterota.c_Deferribacteres.o_Deferribacterales
d_Bacteria.p_Deferribacterota.c_Deferribacteres.o_Deferribacterales.f_Deferribacteraceae
d_Bacteria.p_Firmicutes.c_Bacilli.o_Acholeplasmatales
d_Bacteria.p_Firmicutes.c_Bacilli.o_Acholeplasmatales.f_Acholeplasmataceae
d_Bacteria.p_Firmicutes.c_Negativicutes.o_Veillonellales_Selenomonadales.f_Veillonellaceae
d_Bacteria.p_Actinobacteriota.c_Coriobacteriia.o_Coriobacteriales.f_Eggerthellaceae
d_Bacteria.p_Firmicutes.c_Clostridia.o_Oscillospirales.f__Eubacterium__coprostanoligenes_group
d_Bacteria.p_Firmicutes.c_Bacilli.o_Lactobacillales.f_Vagococcaceae
d_Bacteria.p_Bacteroidota.c_Bacteroidia.o_Bacteroidales.f_Barnesiellaceae
d_Bacteria.p_Actinobacteriota.c_Actinobacteria.o_Micrococcales
d_Bacteria.p_Actinobacteriota.c_Actinobacteria.o_Micrococcales.f_Microbacteriaceae
d_Bacteria.p_Verrucomicrobiota
d_Bacteria.p_Verrucomicrobiota.c_Lentisphaeria
d_Bacteria.p_Verrucomicrobiota.c_Lentisphaeria.o_Victivallales
d_Bacteria.p_Verrucomicrobiota.c_Lentisphaeria.o_Victivallales.f_Victivallaceae
d_Bacteria.p_Verrucomicrobiota.c_Verrucomicrobiae
d_Bacteria.p_Verrucomicrobiota.c_Verrucomicrobiae.o_Verrucomicrobiales
d_Bacteria.p_Verrucomicrobiota.c_Verrucomicrobiae.o_Verrucomicrobiales.f_Akkermansiaceae
d_Bacteria.p_Firmicutes.c_Clostridia.o_Christensenellales
d_Bacteria.p_Firmicutes.c_Clostridia.o_Christensenellales.f_Christensenellaceae

Log of the highest class average	group	LDA (log10)
3.37	post-AFR	2.78
3.32	post-AFR	2.83
2.23	post-AFR	2.01
3.89	post-AFR	3.47
3.52	post-AFR	3.13
3.08	post-AFR	2.67
2.65	post-AFR	2.22
3.37	post-AFR	2.99
2.51	post-AFR	2.33
4.62	post-AFR	3.88
4.57	post-AFR	3.81
4.54	post-AFR	3.83
3.32	post-AFR	2.83
3.32	post-AFR	2.83
4.05	post-AFR	3.65
2.23	post-AFR	2.03
3.89	post-AFR	3.47
3.37	post-AFR	2.97
3.37	post-AFR	3.03
3.37	post-AFR	3.01
3.37	post-AFR	2.99
5.27	pre-AFR	4.90
3.02	pre-AFR	2.67
2.96	pre-AFR	2.67
2.77	pre-AFR	2.38
3.54		
5.39		
2.91		
4.94		
3.73		
4.15		
3.66		
4.26		
3.58		
3.46		
4.23		
3.18		
3.68		
2.41		
3.27		
3.62		
3.18		
2.84		
2.37		
3.00		
2.56		

4.13
4.47
5.49
3.90
4.55
0.00
0.00
0.00
0.00
2.08
1.76
3.90
0.00
2.74
4.05
3.09
3.27
1.20
0.00
3.23
0.00
2.17
2.67
1.39
3.59
4.68
0.00
0.00
3.57
0.00
0.00
4.41
3.48
0.00
0.95
1.04
3.89
2.57
0.00
2.44
0.00
0.00
1.53
2.36
3.12
1.51
2.64

3.78
2.78
1.61
2.22
1.56
2.89
2.05
4.35
1.76
3.62
2.42
1.85
2.13
4.28
1.34
3.09
1.77
1.94
0.00
1.74
0.00
0.00
0.00
2.13
2.87
1.59
0.85
1.64
2.25
1.00
0.00
0.00
2.08
1.58
0.00
0.00
0.00
0.00
0.00
1.00
2.88
0.00
6.00
3.54
3.54
3.54
3.54

5.65
5.65
5.65
5.39
2.91
5.39
4.29
4.29
4.29
3.73
4.15
5.28
3.57
5.02
3.60
3.60
4.54
4.54
4.74
2.85
4.70
4.16
4.16
4.75
4.49
4.47
5.49
5.49
5.49
5.49
4.84
4.84
4.64
4.64
1.58
0.00
0.00
0.00
0.00
2.08
3.90
2.74
2.74
2.74
2.74
3.23
1.20

0.00
3.23
1.87
0.00
4.41
4.41
4.34
4.34
0.00
1.30
3.95
3.93
2.57
0.00
0.00
2.44
2.44
0.00
0.00
0.00
0.00
0.00
0.00
2.78
2.78
1.76
1.76
1.76
1.76
3.62
3.62
3.09
1.74
0.00
0.00
2.08
1.58
1.58
1.00
0.00
0.00
0.00
1.00
1.00
1.00
0.00
0.00

p-value
0.02
0.03
0.04
0.00
0.00
0.03
0.03
0.01
0.04
0.04
0.04
0.03
0.03
0.03
0.03
0.04
0.00
0.01
0.01
0.01
0.01
0.04
0.02
0.01
0.04
-
-
-
-
-
-
-
-
-
-
-
-
-
-
-
-
-
-
-
-
-

-
-
-
-
-
-
-
-
-
-
-
-
-
-
-
-
-
-
-
-
-
-
-
-
-
-
-
-
-
-
-
-
-
-
-
-
-
-
-
-
-
-
-
-
-
-
-

-
-
-
-
0.02
-
-
-
-
-
-
-
-
-
-
-
-
-
-
-
-
-
-
-
-
-
-
-
-
-
-
-
-
-
-
-
-
-
-
-
-
-
-
-
-
-
-

-
-
-
-
-
-
-
-
-
-
-
-
-
-
-
-
-
-
-
-
-
-
-
-
-
-
-
-
-
-
-
-
-
-
-
-
-
-
-
-
-
-
-
-
-
-
-

-
-
-
-
-
-
-
-
-
-
-
-
-
-
-
-
-
-
-
-
-
-
-
-
-
-
-
-
-
-
-
-
-
-
-
-
-
-
-
-
-
-
-
-
-
-
-
