# Supplementary material for: Analysis of Intestinal Microbiota and Metabolic Pathways before and after a 2-Month-Long Hydrolyzed Fish and Rice Starch Hypoallergenic Diet Trial in Pruritic Dogs
Source: Vet Sci. 2023 Jul 21;10(7):478. doi: 10.3390/vetsci10070478 (PMC10384699; doi:10.3390/vetsci10070478)
Supplement: Supplementary file 1 [file vetsci-10-00478-s001.zip › Table S5.pdf]

feature
d_Bacteria,p_Bacteroidota,c_Bacteroidia,o_Bacteroidales,f_Prevotellaceae,g_Paraprevotella
d_Bacteria,p_Firmicutes,c_Clostridia,o_Oscillospirales,f_Ruminococcaceae,g_Anaerofilum
d_Bacteria,p_Actinobacteriota,c_Coriobacteriia,o_Coriobacteriales,f_Coriobacteriaceae,g_Collinsella
d_Bacteria,p_Bacteroidota,c_Bacteroidia,o_Bacteroidales,f_Bacteroidaceae,g_Bacteroides
d_Bacteria,p_Bacteroidota,c_Bacteroidia,o_Bacteroidales,f_Muribaculaceae,g_Muribaculaceae
d_Bacteria,p_Bacteroidota,c_Bacteroidia,o_Bacteroidales,f_Prevotellaceae,g_Alloprevotella
d_Bacteria,p_Campilobacterota,c_Campylobacteria,o_Campylobacterales,f_Campylobacteraceae,g_Campylob
d_Bacteria,p_Campilobacterota,c_Campylobacteria,o_Campylobacterales,f_Helicobacteraceae,g_Helicobacte
d_Bacteria,p_Firmicutes,c_Bacilli,o_Erysipelotrichales,f_Erysipelatoclostridiaceae,g_Erysipelatoclostridium
d_Bacteria,p_Firmicutes,c_Bacilli,o_Erysipelotrichales,f_Erysipelotrichaceae,g_Faecalitalea
d_Bacteria,p_Firmicutes,c_Bacilli,o_Erysipelotrichales,f_Erysipelotrichaceae,g_uncultured
d_Bacteria,p_Firmicutes,c_Clostridia,o_Clostridia_UCG_014,f_Clostridia_UCG_014,g_Clostridia_UCG_014
d_Bacteria,p_Firmicutes,c_Clostridia,o_Clostridiales,f_Clostridiaceae,g_Clostridium_sensu_stricto_1
d_Bacteria,p_Firmicutes,c_Clostridia,o_Lachnospirales,f_Lachnospiraceae,_
d_Bacteria,p_Firmicutes,c_Clostridia,o_Lachnospirales,f_Lachnospiraceae,g_Blautia
d_Bacteria,p_Firmicutes,c_Clostridia,o_Lachnospirales,f_Lachnospiraceae,g_Lachnoclostridium
d_Bacteria,p_Firmicutes,c_Clostridia,o_Lachnospirales,f_Lachnospiraceae,g_Lachnospiraceae_NK4A136_grou
d_Bacteria,p_Firmicutes,c_Clostridia,o_Lachnospirales,f_Lachnospiraceae,g_Sellimonas
d_Bacteria,p_Firmicutes,c_Clostridia,o_Lachnospirales,f_Lachnospiraceae,g_Tyzzerella
d_Bacteria,p_Firmicutes,c_Clostridia,o_Lachnospirales,f_Lachnospiraceae,g__Ruminococcus__gnavus_group
d_Bacteria,p_Firmicutes,c_Clostridia,o_Lachnospirales,f_Lachnospiraceae,g__Ruminococcus__torques_group
d_Bacteria,p_Firmicutes,c_Clostridia,o_Oscillospirales,f_Butyricicoccaceae,g_Butyricicoccus
d_Bacteria,p_Firmicutes,c_Clostridia,o_Oscillospirales,f_Oscillospiraceae,g_Intestinimonas
d_Bacteria,p_Firmicutes,c_Clostridia,o_Oscillospirales,f_Ruminococcaceae,_
d_Bacteria,p_Firmicutes,c_Clostridia,o_Oscillospirales,f_Ruminococcaceae,g_Fournierella
d_Bacteria,p_Firmicutes,c_Clostridia,o_Peptostreptococcales_Tissierellales,f_Peptostreptococcaceae,g_Pepto
d_Bacteria,p_Firmicutes,c_Negativicutes,o_Veillonellales_Selenomonadales,f_Selenomonadaceae,g_Megamo
d_Bacteria,p_Fusobacteriota,c_Fusobacteriia,o_Fusobacteriales,f_Fusobacteriaceae,g_Fusobacterium
d_Bacteria,p_Proteobacteria,c_Gammaproteobacteria,o_Burkholderiales,f_Sutterellaceae,g_Parasutterella
d_Bacteria,p_Proteobacteria,c_Gammaproteobacteria,o_Burkholderiales,f_Sutterellaceae,g_Sutterella
d_Bacteria,p_Actinobacteriota,c_Actinobacteria,o_Actinomycetales,f_Actinomycetaceae,g_Actinomyces
d_Bacteria,p_Actinobacteriota,c_Actinobacteria,o_Actinomycetales,f_Actinomycetaceae,g_Trueperella
d_Bacteria,p_Actinobacteriota,c_Actinobacteria,o_Corynebacteriales,f_Corynebacteriaceae,g_Corynebacteriu
d_Bacteria,p_Actinobacteriota,c_Actinobacteria,o_Corynebacteriales,f_Corynebacteriaceae,g_Lawsonella
d_Bacteria,p_Bacteroidota,c_Bacteroidia,o_Bacteroidales,f_Marinifilaceae,g_Odoribacter
d_Bacteria,p_Bacteroidota,c_Bacteroidia,o_Bacteroidales,f_Porphyromonadaceae,g_Porphyromonas
d_Bacteria,p_Bacteroidota,c_Bacteroidia,o_Bacteroidales,f_Prevotellaceae,g_Prevotella
d_Bacteria,p_Bacteroidota,c_Bacteroidia,o_Bacteroidales,f_Rikenellaceae,g_Alistipes
d_Bacteria,p_Bacteroidota,c_Bacteroidia,o_Bacteroidales,f_Rikenellaceae,g_Rikenellaceae_RC9_gut_group
d_Bacteria,p_Bacteroidota,c_Bacteroidia,o_Bacteroidales,f_Tannerellaceae,g_Parabacteroides
d_Bacteria,p_Bacteroidota,c_Bacteroidia,o_Bacteroidales,f_Tannerellaceae,g_Tannerella
d_Bacteria,p_Desulfobacterota,c_Desulfovibrionia,o_Desulfovibrionales,f_Desulfovibrionaceae,g_Desulfovibr
d_Bacteria,p_Firmicutes,c_Bacilli,o_Erysipelotrichales,f_Erysipelotrichaceae,g_Allobaculum
d_Bacteria,p_Firmicutes,c_Bacilli,o_Erysipelotrichales,f_Erysipelotrichaceae,g_Holdemanella
d_Bacteria,p_Firmicutes,c_Bacilli,o_Erysipelotrichales,f_Erysipelotrichaceae,g_Turicibacter
d_Bacteria,p_Firmicutes,c_Bacilli,o_Lactobacillales,f_Enterococcaceae,g_Enterococcus
d_Bacteria,p_Firmicutes,c_Bacilli,o_Lactobacillales,f_Lactobacillaceae,g_Lactobacillus
d_Bacteria,p_Firmicutes,c_Bacilli,o_Lactobacillales,f_Streptococcaceae,g_Streptococcus
d_Bacteria,p_Firmicutes,c_Clostridia,o_Lachnospirales,f_Lachnospiraceae,g_Johnsonella

d_Bacteria,p_Firmicutes,c_Clostridia,o_Lachnospirales,f_Lachnospiraceae,g_Lachnospiraceae_UCG_009
d_Bacteria,p_Firmicutes,c_Clostridia,o_Lachnospirales,f_Lachnospiraceae,g__Ruminococcus__gauvreauii_gro
d_Bacteria,p_Firmicutes,c_Clostridia,o_Oscillospirales,f_Butyricicoccaceae,_
d_Bacteria,p_Firmicutes,c_Clostridia,o_Oscillospirales,f_Oscillospiraceae,g_Flavonifractor
d_Bacteria,p_Firmicutes,c_Clostridia,o_Oscillospirales,f_Ruminococcaceae,g_Faecalibacterium
d_Bacteria,p_Firmicutes,c_Clostridia,o_Oscillospirales,f_Ruminococcaceae,g_Negativibacillus
d_Bacteria,p_Firmicutes,c_Clostridia,o_Peptostreptococcales_Tissierellales,f_Anaerovoracaceae,g_Family_XII
d_Bacteria,p_Firmicutes,c_Clostridia,o_Peptostreptococcales_Tissierellales,f_Peptostreptococcaceae,g_Pepto
d_Bacteria,p_Firmicutes,c_Clostridia,o_Peptostreptococcales_Tissierellales,f_Peptostreptococcaceae,g_Romb
d_Bacteria,p_Firmicutes,c_Clostridia,o_Peptostreptococcales_Tissierellales,f_Peptostreptococcaceae,g_Terris
d_Bacteria,p_Firmicutes,c_Clostridia,o_Peptostreptococcales_Tissierellales,f_Peptostreptococcales_Tissierella
d_Bacteria,p_Firmicutes,c_Negativicutes,o_Acidaminococcales,f_Acidaminococcaceae,g_Acidaminococcus
d_Bacteria,p_Firmicutes,c_Negativicutes,o_Acidaminococcales,f_Acidaminococcaceae,g_Phascolarctobacteriu
d_Bacteria,p_Proteobacteria,c_Gammaproteobacteria,o_Aeromonadales,f_Succinivibrionaceae,g_Succinivibr
d_Bacteria,p_Proteobacteria,c_Gammaproteobacteria,o_Burkholderiales,f_Burkholderiaceae,g_Ralstonia
d_Bacteria,p_Proteobacteria,c_Gammaproteobacteria,o_Burkholderiales,f_Comamonadaceae,_
d_Bacteria,p_Proteobacteria,c_Gammaproteobacteria,o_Burkholderiales,f_Comamonadaceae,g_Comamonas
d_Bacteria,p_Proteobacteria,c_Gammaproteobacteria,o_Enterobacterales,f_Enterobacteriaceae,g_Escherichi
d_Bacteria,p_Proteobacteria,c_Gammaproteobacteria,o_Enterobacterales,f_Morganellaceae,g_Proteus
d_Bacteria,p_Proteobacteria,c_Gammaproteobacteria,o_Oceanospirillales,f_Marinomonadaceae,g_Marinom
d_Bacteria,p_Proteobacteria,c_Gammaproteobacteria,o_Pseudomonadales,f_Pseudomonadaceae,g_Pseudor
d_Bacteria,p_Synergistota,c_Synergistia,o_Synergistales,f_Synergistaceae,g_Fretibacterium
d_Bacteria,p_Actinobacteriota,c_Actinobacteria,o_Bifidobacteriales,f_Bifidobacteriaceae,g_Bifidobacterium
d_Bacteria,p_Desulfobacterota,c_Desulfovibrionia,o_Desulfovibrionales,f_Desulfovibrionaceae,g_Bilophila
d_Bacteria,p_Firmicutes,c_Bacilli,o_Erysipelotrichales,f_Erysipelatoclostridiaceae,g_Candidatus_Stoquefichus
d_Bacteria,p_Firmicutes,c_Bacilli,o_Erysipelotrichales,f_Erysipelatoclostridiaceae,g_Catenibacterium
d_Bacteria,p_Firmicutes,c_Clostridia,o_Lachnospirales,f_Lachnospiraceae,g_Cellulosilyticum
d_Bacteria,p_Firmicutes,c_Clostridia,o_Oscillospirales,f_Oscillospiraceae,g_Oscillibacter
d_Bacteria,p_Firmicutes,c_Clostridia,o_Oscillospirales,f_Oscillospiraceae,g_UCG_005
d_Bacteria,p_Firmicutes,c_Clostridia,o_Peptococcales,f_Peptococcaceae,g_Peptococcus
d_Bacteria,p_Firmicutes,c_Clostridia,o_Peptostreptococcales_Tissierellales,f_Anaerovoracaceae,g__Eubacter
d_Bacteria,p_Firmicutes,c_Clostridia,o_Clostridiales,f_Clostridiaceae,g_Candidatus_Arthromitus
d_Bacteria,p_Firmicutes,c_Clostridia,o_Lachnospirales,f_Lachnospiraceae,g_Lachnospiraceae
d_Bacteria,p_Firmicutes,c_Clostridia,o_Lachnospirales,f_Lachnospiraceae,g_Roseburia
d_Bacteria,p_Firmicutes,c_Clostridia,o_Lachnospirales,f_Lachnospiraceae,g_Tuzzerella
d_Bacteria,p_Firmicutes,c_Clostridia,o_Oscillospirales,f_Oscillospiraceae,_
d_Bacteria,p_Firmicutes,c_Clostridia,o_Oscillospirales,f_Oscillospiraceae,g_Colidextribacter
d_Bacteria,p_Bacteroidota,c_Bacteroidia,o_Bacteroidales,f_Prevotellaceae,g_Prevotellaceae_Ga6A1_group
d_Bacteria,p_Deferribacterota,c_Deferribacteres,o_Deferribacterales,f_Deferribacteraceae,g_Mucispirillum
d_Bacteria,p_Firmicutes,c_Bacilli,o_Acholeplasmatales,f_Acholeplasmataceae,g_Anaeroplasma
d_Bacteria,p_Firmicutes,c_Bacilli,o_Erysipelotrichales,f_Erysipelatoclostridiaceae,g_Erysipelotrichaceae_UCG_
d_Bacteria,p_Firmicutes,c_Clostridia,o_Lachnospirales,f_Lachnospiraceae,g_Fusicatenibacter
d_Bacteria,p_Firmicutes,c_Clostridia,o_Peptostreptococcales_Tissierellales,f_Peptostreptococcaceae,g_Paeni
d_Bacteria,p_Proteobacteria,c_Gammaproteobacteria,o_Aeromonadales,f_Succinivibrionaceae,g_Anaerobios
d_Bacteria,p_Firmicutes,c_Clostridia,o_Lachnospirales,f_Lachnospiraceae,g_Lachnospira
d_Bacteria,p_Firmicutes,c_Negativicutes,o_Veillonellales_Selenomonadales,f_Veillonellaceae,g_Allisonella
d_Bacteria,p_Firmicutes,c_Negativicutes,o_Veillonellales_Selenomonadales,f_Veillonellaceae,g_Megasphaera
d_Bacteria,p_Firmicutes,c_Clostridia,o_Oscillospirales,f_Ruminococcaceae,g_Phocea
d_Bacteria,p_Firmicutes,c_Clostridia,o_Peptostreptococcales_Tissierellales,f_Peptostreptococcaceae,g_Clostr
d_Bacteria,p_Firmicutes,c_Clostridia,o_Clostridiales,f_Clostridiaceae,g_Sarcina

d_Bacteria,p_Cyanobacteria,c_Vampirivibrionia,o_Gastranaerophilales,f_Gastranaerophilales,g_Gastranaerop
d_Bacteria,p_Actinobacteriota,c_Coriobacteriia,o_Coriobacteriales,f_Eggerthellaceae,g_Slackia
d_Bacteria,p_Actinobacteriota,c_Coriobacteriia,o_Coriobacteriales,f_Eggerthellaceae,g_Parvibacter
d_Bacteria,p_Desulfobacterota,c_Desulfovibrionia,o_Desulfovibrionales,f_Desulfovibrionaceae,g_Mailhella
d_Bacteria,p_Firmicutes,c_Clostridia,o_Oscillospirales,f_Ruminococcaceae,g_Harryflintia
d_Bacteria,p_Firmicutes,c_Clostridia,o_Oscillospirales,f_Ruminococcaceae,g_Incertae_Sedis
d_Bacteria,p_Proteobacteria,c_Gammaproteobacteria,o_Enterobacterales,f_Enterobacteriaceae,_
d_Bacteria,p_Firmicutes,c_Clostridia,o_Peptostreptococcales_Tissierellales,f_Anaerovoracaceae,g__Eubacter
d_Bacteria,p_Firmicutes,c_Clostridia,o_Lachnospirales,f_Lachnospiraceae,g_GCA_900066575
d_Bacteria,p_Firmicutes,c_Clostridia,o_Lachnospirales,f_Lachnospiraceae,g_Anaerostignum
d_Bacteria,p_Firmicutes,c_Bacilli,o_Erysipelotrichales,f_Erysipelotrichaceae,g_Holdemania
d_Bacteria,p_Firmicutes,c_Clostridia,o_Oscillospirales,f__Eubacterium__coprostanoligenes_group,g__Eubact
d_Bacteria,p_Firmicutes,c_Bacilli,o_Lactobacillales,f_Vagococcaceae,g_Vagococcus
d_Bacteria,p_Firmicutes,c_Clostridia,o_Lachnospirales,f_Lachnospiraceae,g_Epulopiscium
d_Bacteria,p_Bacteroidota,c_Bacteroidia,o_Bacteroidales,f_Barnesiellaceae,g_Barnesiella
d_Bacteria,p_Actinobacteriota,c_Actinobacteria,o_Micrococcales,f_Microbacteriaceae,g_Leucobacter
d_Bacteria,p_Firmicutes,c_Bacilli,o_Erysipelotrichales,f_Erysipelotrichaceae,g_Catenisphaera
d_Bacteria,p_Firmicutes,c_Clostridia,o_Oscillospirales,f_Ruminococcaceae,g_UBA1819
d_Bacteria,p_Bacteroidota,c_Bacteroidia,o_Bacteroidales,f_Prevotellaceae,g_Prevotellaceae_UCG_001
d_Bacteria,p_Firmicutes,c_Clostridia,o_Oscillospirales,f_Ruminococcaceae,g_Subdoligranulum
d_Bacteria,p_Verrucomicrobiota,c_Lentisphaeria,o_Victivallales,f_Victivallaceae,g_Victivallis
d_Bacteria,p_Verrucomicrobiota,c_Verrucomicrobiae,o_Verrucomicrobiales,f_Akkermansiaceae,g_Akkerman
d_Bacteria,p_Firmicutes,c_Negativicutes,o_Veillonellales_Selenomonadales,f_Veillonellaceae,g_Dialister
d_Bacteria,p_Firmicutes,c_Clostridia,o_Christensenellales,f_Christensenellaceae,g_Christensenellaceae_R_7_
d_Bacteria
d_Bacteria,p_Actinobacteriota
d_Bacteria,p_Actinobacteriota,c_Coriobacteriia
d_Bacteria,p_Actinobacteriota,c_Coriobacteriia,o_Coriobacteriales
d_Bacteria,p_Actinobacteriota,c_Coriobacteriia,o_Coriobacteriales,f_Coriobacteriaceae
d_Bacteria,p_Bacteroidota
d_Bacteria,p_Bacteroidota,c_Bacteroidia
d_Bacteria,p_Bacteroidota,c_Bacteroidia,o_Bacteroidales
d_Bacteria,p_Bacteroidota,c_Bacteroidia,o_Bacteroidales,f_Bacteroidaceae
d_Bacteria,p_Bacteroidota,c_Bacteroidia,o_Bacteroidales,f_Muribaculaceae
d_Bacteria,p_Bacteroidota,c_Bacteroidia,o_Bacteroidales,f_Prevotellaceae
d_Bacteria,p_Campilobacterota
d_Bacteria,p_Campilobacterota,c_Campylobacteria
d_Bacteria,p_Campilobacterota,c_Campylobacteria,o_Campylobacterales
d_Bacteria,p_Campilobacterota,c_Campylobacteria,o_Campylobacterales,f_Campylobacteraceae
d_Bacteria,p_Campilobacterota,c_Campylobacteria,o_Campylobacterales,f_Helicobacteraceae
d_Bacteria,p_Firmicutes
d_Bacteria,p_Firmicutes,c_Bacilli
d_Bacteria,p_Firmicutes,c_Bacilli,o_Erysipelotrichales
d_Bacteria,p_Firmicutes,c_Bacilli,o_Erysipelotrichales,f_Erysipelatoclostridiaceae
d_Bacteria,p_Firmicutes,c_Bacilli,o_Erysipelotrichales,f_Erysipelotrichaceae
d_Bacteria,p_Firmicutes,c_Clostridia
d_Bacteria,p_Firmicutes,c_Clostridia,o_Clostridia_UCG_014
d_Bacteria,p_Firmicutes,c_Clostridia,o_Clostridia_UCG_014,f_Clostridia_UCG_014
d_Bacteria,p_Firmicutes,c_Clostridia,o_Clostridiales
d_Bacteria,p_Firmicutes,c_Clostridia,o_Clostridiales,f_Clostridiaceae

d_Bacteria,p_Firmicutes,c_Clostridia,o_Lachnospirales
d_Bacteria,p_Firmicutes,c_Clostridia,o_Lachnospirales,f_Lachnospiraceae
d_Bacteria,p_Firmicutes,c_Clostridia,o_Oscillospirales
d_Bacteria,p_Firmicutes,c_Clostridia,o_Oscillospirales,f_Butyricicoccaceae
d_Bacteria,p_Firmicutes,c_Clostridia,o_Oscillospirales,f_Oscillospiraceae
d_Bacteria,p_Firmicutes,c_Clostridia,o_Oscillospirales,f_Ruminococcaceae
d_Bacteria,p_Firmicutes,c_Clostridia,o_Peptostreptococcales_Tissierellales
d_Bacteria,p_Firmicutes,c_Clostridia,o_Peptostreptococcales_Tissierellales,f_Peptostreptococcaceae
d_Bacteria,p_Firmicutes,c_Negativicutes
d_Bacteria,p_Firmicutes,c_Negativicutes,o_Veillonellales_Selenomonadales
d_Bacteria,p_Firmicutes,c_Negativicutes,o_Veillonellales_Selenomonadales,f_Selenomonadaceae
d_Bacteria,p_Fusobacteriota
d_Bacteria,p_Fusobacteriota,c_Fusobacteriia
d_Bacteria,p_Fusobacteriota,c_Fusobacteriia,o_Fusobacteriales
d_Bacteria,p_Fusobacteriota,c_Fusobacteriia,o_Fusobacteriales,f_Fusobacteriaceae
d_Bacteria,p_Proteobacteria
d_Bacteria,p_Proteobacteria,c_Gammaproteobacteria
d_Bacteria,p_Proteobacteria,c_Gammaproteobacteria,o_Burkholderiales
d_Bacteria,p_Proteobacteria,c_Gammaproteobacteria,o_Burkholderiales,f_Sutterellaceae
d_Bacteria,p_Actinobacteriota,c_Actinobacteria
d_Bacteria,p_Actinobacteriota,c_Actinobacteria,o_Actinomycetales
d_Bacteria,p_Actinobacteriota,c_Actinobacteria,o_Actinomycetales,f_Actinomycetaceae
d_Bacteria,p_Actinobacteriota,c_Actinobacteria,o_Corynebacteriales
d_Bacteria,p_Actinobacteriota,c_Actinobacteria,o_Corynebacteriales,f_Corynebacteriaceae
d_Bacteria,p_Bacteroidota,c_Bacteroidia,o_Bacteroidales,f_Marinifilaceae
d_Bacteria,p_Bacteroidota,c_Bacteroidia,o_Bacteroidales,f_Porphyromonadaceae
d_Bacteria,p_Bacteroidota,c_Bacteroidia,o_Bacteroidales,f_Rikenellaceae
d_Bacteria,p_Bacteroidota,c_Bacteroidia,o_Bacteroidales,f_Tannerellaceae
d_Bacteria,p_Desulfobacterota
d_Bacteria,p_Desulfobacterota,c_Desulfovibrionia
d_Bacteria,p_Desulfobacterota,c_Desulfovibrionia,o_Desulfovibrionales
d_Bacteria,p_Desulfobacterota,c_Desulfovibrionia,o_Desulfovibrionales,f_Desulfovibrionaceae
d_Bacteria,p_Firmicutes,c_Bacilli,o_Lactobacillales
d_Bacteria,p_Firmicutes,c_Bacilli,o_Lactobacillales,f_Enterococcaceae
d_Bacteria,p_Firmicutes,c_Bacilli,o_Lactobacillales,f_Lactobacillaceae
d_Bacteria,p_Firmicutes,c_Bacilli,o_Lactobacillales,f_Streptococcaceae
d_Bacteria,p_Firmicutes,c_Clostridia,o_Peptostreptococcales_Tissierellales,f_Anaerovoracaceae
d_Bacteria,p_Firmicutes,c_Clostridia,o_Peptostreptococcales_Tissierellales,f_Peptostreptococcales_Tissierell[illegible]
d_Bacteria,p_Firmicutes,c_Negativicutes,o_Acidaminococcales
d_Bacteria,p_Firmicutes,c_Negativicutes,o_Acidaminococcales,f_Acidaminococcaceae
d_Bacteria,p_Proteobacteria,c_Gammaproteobacteria,o_Aeromonadales
d_Bacteria,p_Proteobacteria,c_Gammaproteobacteria,o_Aeromonadales,f_Succinivibrionaceae
d_Bacteria,p_Proteobacteria,c_Gammaproteobacteria,o_Burkholderiales,f_Burkholderiaceae
d_Bacteria,p_Proteobacteria,c_Gammaproteobacteria,o_Burkholderiales,f_Comamonadaceae
d_Bacteria,p_Proteobacteria,c_Gammaproteobacteria,o_Enterobacterales
d_Bacteria,p_Proteobacteria,c_Gammaproteobacteria,o_Enterobacterales,f_Enterobacteriaceae
d_Bacteria,p_Proteobacteria,c_Gammaproteobacteria,o_Enterobacterales,f_Morganellaceae
d_Bacteria,p_Proteobacteria,c_Gammaproteobacteria,o_Oceanospirillales
d_Bacteria,p_Proteobacteria,c_Gammaproteobacteria,o_Oceanospirillales,f_Marinomonadaceae
d_Bacteria,p_Proteobacteria,c_Gammaproteobacteria,o_Pseudomonadales

d_Bacteria,p_Proteobacteria,c_Gammaproteobacteria,o_Pseudomonadales,f_Pseudomonadaceae
d_Bacteria,p_Synergistota
d_Bacteria,p_Synergistota,c_Synergistia
d_Bacteria,p_Synergistota,c_Synergistia,o_Synergistales
d_Bacteria,p_Synergistota,c_Synergistia,o_Synergistales,f_Synergistaceae
d_Bacteria,p_Actinobacteriota,c_Actinobacteria,o_Bifidobacteriales
d_Bacteria,p_Actinobacteriota,c_Actinobacteria,o_Bifidobacteriales,f_Bifidobacteriaceae
d_Bacteria,p_Firmicutes,c_Clostridia,o_Peptococcales
d_Bacteria,p_Firmicutes,c_Clostridia,o_Peptococcales,f_Peptococcaceae
d_Bacteria,p_Deferribacterota
d_Bacteria,p_Deferribacterota,c_Deferribacteres
d_Bacteria,p_Deferribacterota,c_Deferribacteres,o_Deferribacterales
d_Bacteria,p_Deferribacterota,c_Deferribacteres,o_Deferribacterales,f_Deferribacteraceae
d_Bacteria,p_Firmicutes,c_Bacilli,o_Acholeplasmatales
d_Bacteria,p_Firmicutes,c_Bacilli,o_Acholeplasmatales,f_Acholeplasmataceae
d_Bacteria,p_Firmicutes,c_Negativicutes,o_Veillonellales_Selenomonadales,f_Veillonellaceae
d_Bacteria,p_Cyanobacteria
d_Bacteria,p_Cyanobacteria,c_Vampirivibrionia
d_Bacteria,p_Cyanobacteria,c_Vampirivibrionia,o_Gastranaerophilales
d_Bacteria,p_Cyanobacteria,c_Vampirivibrionia,o_Gastranaerophilales,f_Gastranaerophilales
d_Bacteria,p_Actinobacteriota,c_Coriobacteriia,o_Coriobacteriales,f_Eggerthellaceae
d_Bacteria,p_Firmicutes,c_Clostridia,o_Oscillospirales,f__Eubacterium__coprostanoligenes_group
d_Bacteria,p_Firmicutes,c_Bacilli,o_Lactobacillales,f_Vagococcaceae
d_Bacteria,p_Bacteroidota,c_Bacteroidia,o_Bacteroidales,f_Barnesiellaceae
d_Bacteria,p_Actinobacteriota,c_Actinobacteria,o_Micrococcales
d_Bacteria,p_Actinobacteriota,c_Actinobacteria,o_Micrococcales,f_Microbacteriaceae
d_Bacteria,p_Verrucomicrobiota
d_Bacteria,p_Verrucomicrobiota,c_Lentisphaeria
d_Bacteria,p_Verrucomicrobiota,c_Lentisphaeria,o_Victivallales
d_Bacteria,p_Verrucomicrobiota,c_Lentisphaeria,o_Victivallales,f_Victivallaceae
d_Bacteria,p_Verrucomicrobiota,c_Verrucomicrobiae
d_Bacteria,p_Verrucomicrobiota,c_Verrucomicrobiae,o_Verrucomicrobiales
d_Bacteria,p_Verrucomicrobiota,c_Verrucomicrobiae,o_Verrucomicrobiales,f_Akkermansiaceae
d_Bacteria,p_Firmicutes,c_Clostridia,o_Christensenellales
d_Bacteria,p_Firmicutes,c_Clostridia,o_Christensenellales,f_Christensenellaceae

Log of the highest class average	group	LDA (log10)	p-value
2.85	post-D	2.86	0.03
3.62	pre-D	3.32	0.03
3.36			-
5.42			-
3.05			-
5.03			-
3.93			-
4.09			-
3.43			-
3.76			-
4.42			-
3.75			-
4.20			-
3.92			-
4.28			-
3.36			-
3.90			-
2.30			-
2.76			-
3.74			-
3.09			-
3.11			-
2.31			-
3.21			-
2.37			-
4.00			-
4.69			-
5.43			-
3.82			-
4.72			-
0.00			-
0.00			-
0.00			-
0.00			-
0.00			-
0.74			-
5.05			-
3.26			-
1.58			-
3.49			-
0.00			-
2.90			-
3.93			-
3.19			-
2.87			-
1.58			-
1.85			-
2.86			-
0.00			-

	2.46	-
	2.91	-
	0.91	-
	2.70	-
	4.59	-
	3.48	-
	0.00	-
	0.00	-
	3.28	-
	2.16	-
	0.00	-
	0.00	-
	4.31	-
	3.15	-
	0.00	-
	1.54	-
	0.74	-
	4.00	-
	1.32	-
	0.00	-
	0.86	-
	0.00	-
	2.81	-
	2.98	-
	2.48	-
	3.50	-
	0.66	-
	2.84	-
	3.75	-
	2.76	-
	2.32	-
	1.45	-
	0.86	-
	3.26	-
	3.64	-
	2.55	-
	2.93	-
	4.09	-
	2.39	-
	3.62	-
	2.79	-
	2.26	-
	2.67	-
	3.81	-
	2.99	-
	1.54	-
	0.00	-
	1.53	-
	0.00	-
	3.76	-

3.05	-
1.79	-
1.13	-
1.30	-
0.00	-
1.24	-
2.36	-
1.82	-
2.33	-
2.51	-
0.00	-
1.49	-
0.00	-
3.78	-
3.15	-
0.00	-
0.00	-
1.30	-
2.15	-
2.32	-
1.87	-
2.30	-
0.00	-
1.88	-
6.00	-
3.48	-
3.37	-
3.37	-
3.36	-
5.64	-
5.64	-
5.64	-
5.42	-
3.05	-
5.31	-
4.22	-
4.22	-
4.22	-
3.93	-
4.09	-
5.41	-
4.71	-
4.69	-
3.82	-
4.63	-
5.13	-
3.75	-
3.75	-
4.21	-
4.21	-

4.76	-
4.76	-
4.70	-
3.11	-
3.92	-
4.66	-
4.10	-
4.09	-
4.84	-
4.69	-
4.69	-
5.43	-
5.43	-
5.43	-
5.43	-
4.88	-
4.88	-
4.76	-
4.76	-
2.81	-
0.00	-
0.00	-
0.00	-
0.00	-
0.00	-
0.74	-
3.26	-
3.49	-
3.25	-
3.25	-
3.25	-
3.25	-
2.92	-
1.58	-
1.85	-
2.86	-
2.44	-
0.00	-
4.31	-
4.31	-
3.90	-
3.90	-
0.00	-
1.60	-
4.01	-
4.01	-
1.32	-
0.00	-
0.00	-
0.86	-

	0.86	-
	0.00	-
	0.00	-
	0.00	-
	0.00	-
	2.81	-
	2.81	-
	2.76	-
	2.76	-
	2.39	-
	2.39	-
	2.39	-
	2.39	-
	3.62	-
	3.62	-
	1.54	-
	3.05	-
	3.05	-
	3.05	-
	3.05	-
	1.88	-
	1.49	-
	0.00	-
	3.15	-
	0.00	-
	0.00	-
	2.44	-
	1.87	-
	1.87	-
	1.87	-
	2.30	-
	2.30	-
	2.30	-
	1.88	-
	1.88	-
