# Supplementary material for: Analysis of Intestinal Microbiota and Metabolic Pathways before and after a 2-Month-Long Hydrolyzed Fish and Rice Starch Hypoallergenic Diet Trial in Pruritic Dogs
Source: Vet Sci. 2023 Jul 21;10(7):478. doi: 10.3390/vetsci10070478 (PMC10384699; doi:10.3390/vetsci10070478)
Supplement: Supplementary file 1 [file vetsci-10-00478-s001.zip › table S6.pdf]

feature
d_Bacteria.p_Firmicutes.c_Clostridia.o_Lachnospirales.f_Lachnospiraceae.g__Ruminococcus__gauvreauii_gro
d_Bacteria.p_Firmicutes.c_Clostridia.o_Lachnospirales.f_Lachnospiraceae.g_Tuzzerella
d_Bacteria.p_Firmicutes.c_Bacilli.o_Erysipelotrichales.f_Erysipelotrichaceae.g_uncultured
d_Bacteria.p_Firmicutes.c_Negativicutes.o_Acidaminococcales
d_Bacteria.p_Firmicutes.c_Negativicutes.o_Acidaminococcales.f_Acidaminococcaceae
d_Bacteria.p_Firmicutes.c_Negativicutes.o_Acidaminococcales.f_Acidaminococcaceae.g_Phascolarctobacteri
d_Bacteria.p_Actinobacteriota.c_Coriobacteriia.o_Coriobacteriales.f_Coriobacteriaceae
d_Bacteria.p_Actinobacteriota.c_Coriobacteriia.o_Coriobacteriales.f_Coriobacteriaceae.g_Collinsella
d_Bacteria.p_Actinobacteriota.c_Coriobacteriia
d_Bacteria.p_Actinobacteriota.c_Coriobacteriia.o_Coriobacteriales
d_Bacteria.p_Firmicutes.c_Bacilli.o_Erysipelotrichales.f_Erysipelotrichaceae
d_Bacteria.p_Firmicutes.c_Bacilli.o_Erysipelotrichales
d_Bacteria.p_Bacteroidota.c_Bacteroidia.o_Bacteroidales.f_Bacteroidaceae.g_Bacteroides
d_Bacteria.p_Bacteroidota.c_Bacteroidia.o_Bacteroidales.f_Muribaculaceae.g_Muribaculaceae
d_Bacteria.p_Bacteroidota.c_Bacteroidia.o_Bacteroidales.f_Prevotellaceae.g_Alloprevotella
d_Bacteria.p_Campilobacterota.c_Campylobacteria.o_Campylobacterales.f_Campylobacteraceae.g_Campylob
d_Bacteria.p_Campilobacterota.c_Campylobacteria.o_Campylobacterales.f_Helicobacteraceae.g_Helicobacte
d_Bacteria.p_Firmicutes.c_Bacilli.o_Erysipelotrichales.f_Erysipelatoclostridiaceae.g_Erysipelatoclostridium
d_Bacteria.p_Firmicutes.c_Bacilli.o_Erysipelotrichales.f_Erysipelotrichaceae.g_Faecalitalea
d_Bacteria.p_Firmicutes.c_Clostridia.o_Clostridia_UCG_014.f_Clostridia_UCG_014.g_Clostridia_UCG_014
d_Bacteria.p_Firmicutes.c_Clostridia.o_Clostridiales.f_Clostridiaceae.g_Clostridium_sensu_stricto_1
d_Bacteria.p_Firmicutes.c_Clostridia.o_Lachnospirales.f_Lachnospiraceae._
d_Bacteria.p_Firmicutes.c_Clostridia.o_Lachnospirales.f_Lachnospiraceae.g_Blautia
d_Bacteria.p_Firmicutes.c_Clostridia.o_Lachnospirales.f_Lachnospiraceae.g_Lachnoclostridium
d_Bacteria.p_Firmicutes.c_Clostridia.o_Lachnospirales.f_Lachnospiraceae.g_Lachnospiraceae_NK4A136_grou
d_Bacteria.p_Firmicutes.c_Clostridia.o_Lachnospirales.f_Lachnospiraceae.g_Sellimonas
d_Bacteria.p_Firmicutes.c_Clostridia.o_Lachnospirales.f_Lachnospiraceae.g_Tyzzerella
d_Bacteria.p_Firmicutes.c_Clostridia.o_Lachnospirales.f_Lachnospiraceae.g__Ruminococcus__gnavus_group
d_Bacteria.p_Firmicutes.c_Clostridia.o_Lachnospirales.f_Lachnospiraceae.g__Ruminococcus__torques_group
d_Bacteria.p_Firmicutes.c_Clostridia.o_Oscillospirales.f_Butyricicoccaceae.g_Butyricicoccus
d_Bacteria.p_Firmicutes.c_Clostridia.o_Oscillospirales.f_Oscillospiraceae.g_Intestinimonas
d_Bacteria.p_Firmicutes.c_Clostridia.o_Oscillospirales.f_Ruminococcaceae._
d_Bacteria.p_Firmicutes.c_Clostridia.o_Oscillospirales.f_Ruminococcaceae.g_Fournierella
d_Bacteria.p_Firmicutes.c_Clostridia.o_Peptostreptococcales_Tissierellales.f_Peptostreptococcaceae.g_Peptc
d_Bacteria.p_Firmicutes.c_Negativicutes.o_Veillonellales_Selenomonadales.f_Selenomonadaceae.g_Megamc
d_Bacteria.p_Fusobacteriota.c_Fusobacteriia.o_Fusobacteriales.f_Fusobacteriaceae.g_Fusobacterium
d_Bacteria.p_Proteobacteria.c_Gammaproteobacteria.o_Burkholderiales.f_Sutterellaceae.g_Parasutterella
d_Bacteria.p_Proteobacteria.c_Gammaproteobacteria.o_Burkholderiales.f_Sutterellaceae.g_Sutterella
d_Bacteria.p_Actinobacteriota.c_Actinobacteria.o_Actinomycetales.f_Actinomycetaceae.g_Actinomyces
d_Bacteria.p_Actinobacteriota.c_Actinobacteria.o_Actinomycetales.f_Actinomycetaceae.g_Trueperella
d_Bacteria.p_Actinobacteriota.c_Actinobacteria.o_Corynebacteriales.f_Corynebacteriaceae.g_Corynebacteri
d_Bacteria.p_Actinobacteriota.c_Actinobacteria.o_Corynebacteriales.f_Corynebacteriaceae.g_Lawsonella
d_Bacteria.p_Bacteroidota.c_Bacteroidia.o_Bacteroidales.f_Marinifilaceae.g_Odoribacter
d_Bacteria.p_Bacteroidota.c_Bacteroidia.o_Bacteroidales.f_Porphyromonadaceae.g_Porphyromonas
d_Bacteria.p_Bacteroidota.c_Bacteroidia.o_Bacteroidales.f_Prevotellaceae.g_Prevotella
d_Bacteria.p_Bacteroidota.c_Bacteroidia.o_Bacteroidales.f_Rikenellaceae.g_Alistipes
d_Bacteria.p_Bacteroidota.c_Bacteroidia.o_Bacteroidales.f_Rikenellaceae.g_Rikenellaceae_RC9_gut_group
d_Bacteria.p_Bacteroidota.c_Bacteroidia.o_Bacteroidales.f_Tannerellaceae.g_Parabacteroides
d_Bacteria.p_Bacteroidota.c_Bacteroidia.o_Bacteroidales.f_Tannerellaceae.g_Tannerella

d_Bacteria.p_Desulfobacterota.c_Desulfovibrionia.o_Desulfovibrionales.f_Desulfovibrionaceae.g_Desulfovibr
d_Bacteria.p_Firmicutes.c_Bacilli.o_Erysipelotrichales.f_Erysipelotrichaceae.g_Allobaculum
d_Bacteria.p_Firmicutes.c_Bacilli.o_Erysipelotrichales.f_Erysipelotrichaceae.g_Holdemanella
d_Bacteria.p_Firmicutes.c_Bacilli.o_Erysipelotrichales.f_Erysipelotrichaceae.g_Turicibacter
d_Bacteria.p_Firmicutes.c_Bacilli.o_Lactobacillales.f_Enterococcaceae.g_Enterococcus
d_Bacteria.p_Firmicutes.c_Bacilli.o_Lactobacillales.f_Lactobacillaceae.g_Lactobacillus
d_Bacteria.p_Firmicutes.c_Bacilli.o_Lactobacillales.f_Streptococcaceae.g_Streptococcus
d_Bacteria.p_Firmicutes.c_Clostridia.o_Lachnospirales.f_Lachnospiraceae.g_Johnsonella
d_Bacteria.p_Firmicutes.c_Clostridia.o_Lachnospirales.f_Lachnospiraceae.g_Lachnospiraceae_UCG_009
d_Bacteria.p_Firmicutes.c_Clostridia.o_Oscillospirales.f_Butyricicoccaceae._
d_Bacteria.p_Firmicutes.c_Clostridia.o_Oscillospirales.f_Oscillospiraceae.g_Flavonifractor
d_Bacteria.p_Firmicutes.c_Clostridia.o_Oscillospirales.f_Ruminococcaceae.g_Faecalibacterium
d_Bacteria.p_Firmicutes.c_Clostridia.o_Oscillospirales.f_Ruminococcaceae.g_Negativibacillus
d_Bacteria.p_Firmicutes.c_Clostridia.o_Peptostreptococcales_Tissierellales.f_Anaerovoracaceae.g_Family_XII
d_Bacteria.p_Firmicutes.c_Clostridia.o_Peptostreptococcales_Tissierellales.f_Peptostreptococcaceae.g_Peptc
d_Bacteria.p_Firmicutes.c_Clostridia.o_Peptostreptococcales_Tissierellales.f_Peptostreptococcaceae.g_Romb
d_Bacteria.p_Firmicutes.c_Clostridia.o_Peptostreptococcales_Tissierellales.f_Peptostreptococcaceae.g_Terris
d_Bacteria.p_Firmicutes.c_Clostridia.o_Peptostreptococcales_Tissierellales.f_Peptostreptococcales_Tissierella
d_Bacteria.p_Firmicutes.c_Negativicutes.o_Acidaminococcales.f_Acidaminococcaceae.g_Acidaminococcus
d_Bacteria.p_Proteobacteria.c_Gammaproteobacteria.o_Aeromonadales.f_Succinivibrionaceae.g_Succinivibr
d_Bacteria.p_Proteobacteria.c_Gammaproteobacteria.o_Burkholderiales.f_Burkholderiaceae.g_Ralstonia
d_Bacteria.p_Proteobacteria.c_Gammaproteobacteria.o_Burkholderiales.f_Comamonadaceae._
d_Bacteria.p_Proteobacteria.c_Gammaproteobacteria.o_Burkholderiales.f_Comamonadaceae.g_Comamonas
d_Bacteria.p_Proteobacteria.c_Gammaproteobacteria.o_Enterobacterales.f_Enterobacteriaceae.g_Escherichi
d_Bacteria.p_Proteobacteria.c_Gammaproteobacteria.o_Enterobacterales.f_Morganellaceae.g_Proteus
d_Bacteria.p_Proteobacteria.c_Gammaproteobacteria.o_Oceanospirillales.f_Marinomonadaceae.g_Marinom
d_Bacteria.p_Proteobacteria.c_Gammaproteobacteria.o_Pseudomonadales.f_Pseudomonadaceae.g_Pseudor
d_Bacteria.p_Synergistota.c_Synergistia.o_Synergistales.f_Synergistaceae.g_Fretibacterium
d_Bacteria.p_Actinobacteriota.c_Actinobacteria.o_Bifidobacteriales.f_Bifidobacteriaceae.g_Bifidobacterium
d_Bacteria.p_Desulfobacterota.c_Desulfovibrionia.o_Desulfovibrionales.f_Desulfovibrionaceae.g_Bilophila
d_Bacteria.p_Firmicutes.c_Bacilli.o_Erysipelotrichales.f_Erysipelatoclostridiaceae.g_Candidatus_Stoquefichus
d_Bacteria.p_Firmicutes.c_Bacilli.o_Erysipelotrichales.f_Erysipelatoclostridiaceae.g_Catenibacterium
d_Bacteria.p_Firmicutes.c_Clostridia.o_Lachnospirales.f_Lachnospiraceae.g_Cellulosilyticum
d_Bacteria.p_Firmicutes.c_Clostridia.o_Oscillospirales.f_Oscillospiraceae.g_Oscillibacter
d_Bacteria.p_Firmicutes.c_Clostridia.o_Oscillospirales.f_Oscillospiraceae.g_UCG_005
d_Bacteria.p_Firmicutes.c_Clostridia.o_Peptococcales.f_Peptococcaceae.g_Peptococcus
d_Bacteria.p_Firmicutes.c_Clostridia.o_Peptostreptococcales_Tissierellales.f_Anaerovoracaceae.g__Eubacter
d_Bacteria.p_Firmicutes.c_Clostridia.o_Clostridiales.f_Clostridiaceae.g_Candidatus_Arthromitus
d_Bacteria.p_Firmicutes.c_Clostridia.o_Lachnospirales.f_Lachnospiraceae.g_Lachnospiraceae
d_Bacteria.p_Firmicutes.c_Clostridia.o_Lachnospirales.f_Lachnospiraceae.g_Roseburia
d_Bacteria.p_Firmicutes.c_Clostridia.o_Oscillospirales.f_Oscillospiraceae._
d_Bacteria.p_Firmicutes.c_Clostridia.o_Oscillospirales.f_Oscillospiraceae.g_Colidextribacter
d_Bacteria.p_Bacteroidota.c_Bacteroidia.o_Bacteroidales.f_Prevotellaceae.g_Prevotellaceae_Ga6A1_group
d_Bacteria.p_Deferribacterota.c_Deferribacteres.o_Deferribacterales.f_Deferribacteraceae.g_Mucispirillum
d_Bacteria.p_Firmicutes.c_Bacilli.o_Acholeplasmatales.f_Acholeplasmataceae.g_Anaeroplasma
d_Bacteria.p_Firmicutes.c_Bacilli.o_Erysipelotrichales.f_Erysipelatoclostridiaceae.g_Erysipelotrichaceae_UCG_
d_Bacteria.p_Firmicutes.c_Clostridia.o_Lachnospirales.f_Lachnospiraceae.g_Fusicatenibacter
d_Bacteria.p_Firmicutes.c_Clostridia.o_Peptostreptococcales_Tissierellales.f_Peptostreptococcaceae.g_Paeni
d_Bacteria.p_Proteobacteria.c_Gammaproteobacteria.o_Aeromonadales.f_Succinivibrionaceae.g_Anaerobios
d_Bacteria.p_Firmicutes.c_Clostridia.o_Lachnospirales.f_Lachnospiraceae.g_Lachnospira

d_Bacteria.p_Firmicutes.c_Negativicutes.o_Veillonellales_Selenomonadales.f_Veillonellaceae.g_Allisonella
d_Bacteria.p_Firmicutes.c_Negativicutes.o_Veillonellales_Selenomonadales.f_Veillonellaceae.g_Megasphaera
d_Bacteria.p_Firmicutes.c_Clostridia.o_Oscillospirales.f_Ruminococcaceae.g_Phocea
d_Bacteria.p_Firmicutes.c_Clostridia.o_Peptostreptococcales_Tissierellales.f_Peptostreptococcaceae.g_Clostr
d_Bacteria.p_Firmicutes.c_Clostridia.o_Clostridiales.f_Clostridiaceae.g_Sarcina
d_Bacteria.p_Cyanobacteria.c_Vampirivibrionia.o_Gastranaerophilales.f_Gastranaerophilales.g_Gastranaerop
d_Bacteria.p_Actinobacteriota.c_Coriobacteriia.o_Coriobacteriales.f_Eggerthellaceae.g_Slackia
d_Bacteria.p_Actinobacteriota.c_Coriobacteriia.o_Coriobacteriales.f_Eggerthellaceae.g_Parvibacter
d_Bacteria.p_Bacteroidota.c_Bacteroidia.o_Bacteroidales.f_Prevotellaceae.g_Paraprevotella
d_Bacteria.p_Desulfobacterota.c_Desulfovibrionia.o_Desulfovibrionales.f_Desulfovibrionaceae.g_Mailhella
d_Bacteria.p_Firmicutes.c_Clostridia.o_Oscillospirales.f_Ruminococcaceae.g_Harryflintia
d_Bacteria.p_Firmicutes.c_Clostridia.o_Oscillospirales.f_Ruminococcaceae.g_Incertae_Sedis
d_Bacteria.p_Proteobacteria.c_Gammaproteobacteria.o_Enterobacterales.f_Enterobacteriaceae._
d_Bacteria.p_Firmicutes.c_Clostridia.o_Peptostreptococcales_Tissierellales.f_Anaerovoracaceae.g__Eubacter
d_Bacteria.p_Firmicutes.c_Clostridia.o_Lachnospirales.f_Lachnospiraceae.g_GCA_900066575
d_Bacteria.p_Firmicutes.c_Clostridia.o_Lachnospirales.f_Lachnospiraceae.g_Anaerostignum
d_Bacteria.p_Firmicutes.c_Clostridia.o_Oscillospirales.f_Ruminococcaceae.g_Anaerofilum
d_Bacteria.p_Firmicutes.c_Bacilli.o_Erysipelotrichales.f_Erysipelotrichaceae.g_Holdemania
d_Bacteria.p_Firmicutes.c_Clostridia.o_Oscillospirales.f__Eubacterium__coprostanoligenes_group.g__Eubact
d_Bacteria.p_Firmicutes.c_Bacilli.o_Lactobacillales.f_Vagococcaceae.g_Vagococcus
d_Bacteria.p_Firmicutes.c_Clostridia.o_Lachnospirales.f_Lachnospiraceae.g_Epulopiscium
d_Bacteria.p_Bacteroidota.c_Bacteroidia.o_Bacteroidales.f_Barnesiellaceae.g_Barnesiella
d_Bacteria.p_Actinobacteriota.c_Actinobacteria.o_Micrococcales.f_Microbacteriaceae.g_Leucobacter
d_Bacteria.p_Firmicutes.c_Bacilli.o_Erysipelotrichales.f_Erysipelotrichaceae.g_Catenisphaera
d_Bacteria.p_Firmicutes.c_Clostridia.o_Oscillospirales.f_Ruminococcaceae.g_UBA1819
d_Bacteria.p_Bacteroidota.c_Bacteroidia.o_Bacteroidales.f_Prevotellaceae.g_Prevotellaceae_UCG_001
d_Bacteria.p_Firmicutes.c_Clostridia.o_Oscillospirales.f_Ruminococcaceae.g_Subdoligranulum
d_Bacteria.p_Verrucomicrobiota.c_Lentisphaeria.o_Victivallales.f_Victivallaceae.g_Victivallis
d_Bacteria.p_Verrucomicrobiota.c_Verrucomicrobiae.o_Verrucomicrobiales.f_Akkermansiaceae.g_Akkerman
d_Bacteria.p_Firmicutes.c_Negativicutes.o_Veillonellales_Selenomonadales.f_Veillonellaceae.g_Dialister
d_Bacteria.p_Firmicutes.c_Clostridia.o_Christensenellales.f_Christensenellaceae.g_Christensenellaceae_R_7_
d_Bacteria
d_Bacteria.p_Actinobacteriota
d_Bacteria.p_Bacteroidota
d_Bacteria.p_Bacteroidota.c_Bacteroidia
d_Bacteria.p_Bacteroidota.c_Bacteroidia.o_Bacteroidales
d_Bacteria.p_Bacteroidota.c_Bacteroidia.o_Bacteroidales.f_Bacteroidaceae
d_Bacteria.p_Bacteroidota.c_Bacteroidia.o_Bacteroidales.f_Muribaculaceae
d_Bacteria.p_Bacteroidota.c_Bacteroidia.o_Bacteroidales.f_Prevotellaceae
d_Bacteria.p_Campilobacterota
d_Bacteria.p_Campilobacterota.c_Campylobacteria
d_Bacteria.p_Campilobacterota.c_Campylobacteria.o_Campylobacterales
d_Bacteria.p_Campilobacterota.c_Campylobacteria.o_Campylobacterales.f_Campylobacteraceae
d_Bacteria.p_Campilobacterota.c_Campylobacteria.o_Campylobacterales.f_Helicobacteraceae
d_Bacteria.p_Firmicutes
d_Bacteria.p_Firmicutes.c_Bacilli
d_Bacteria.p_Firmicutes.c_Bacilli.o_Erysipelotrichales.f_Erysipelatoclostridiaceae
d_Bacteria.p_Firmicutes.c_Clostridia
d_Bacteria.p_Firmicutes.c_Clostridia.o_Clostridia_UCG_014
d_Bacteria.p_Firmicutes.c_Clostridia.o_Clostridia_UCG_014.f_Clostridia_UCG_014

d_Bacteria.p_Firmicutes.c_Clostridia.o_Clostridiales
d_Bacteria.p_Firmicutes.c_Clostridia.o_Clostridiales.f_Clostridiaceae
d_Bacteria.p_Firmicutes.c_Clostridia.o_Lachnospirales
d_Bacteria.p_Firmicutes.c_Clostridia.o_Lachnospirales.f_Lachnospiraceae
d_Bacteria.p_Firmicutes.c_Clostridia.o_Oscillospirales
d_Bacteria.p_Firmicutes.c_Clostridia.o_Oscillospirales.f_Butyricicoccaceae
d_Bacteria.p_Firmicutes.c_Clostridia.o_Oscillospirales.f_Oscillospiraceae
d_Bacteria.p_Firmicutes.c_Clostridia.o_Oscillospirales.f_Ruminococcaceae
d_Bacteria.p_Firmicutes.c_Clostridia.o_Peptostreptococcales_Tissierellales
d_Bacteria.p_Firmicutes.c_Clostridia.o_Peptostreptococcales_Tissierellales.f_Peptostreptococcaceae
d_Bacteria.p_Firmicutes.c_Negativicutes
d_Bacteria.p_Firmicutes.c_Negativicutes.o_Veillonellales_Selenomonadales
d_Bacteria.p_Firmicutes.c_Negativicutes.o_Veillonellales_Selenomonadales.f_Selenomonadaceae
d_Bacteria.p_Fusobacteriota
d_Bacteria.p_Fusobacteriota.c_Fusobacteriia
d_Bacteria.p_Fusobacteriota.c_Fusobacteriia.o_Fusobacteriales
d_Bacteria.p_Fusobacteriota.c_Fusobacteriia.o_Fusobacteriales.f_Fusobacteriaceae
d_Bacteria.p_Proteobacteria
d_Bacteria.p_Proteobacteria.c_Gammaproteobacteria
d_Bacteria.p_Proteobacteria.c_Gammaproteobacteria.o_Burkholderiales
d_Bacteria.p_Proteobacteria.c_Gammaproteobacteria.o_Burkholderiales.f_Sutterellaceae
d_Bacteria.p_Actinobacteriota.c_Actinobacteria
d_Bacteria.p_Actinobacteriota.c_Actinobacteria.o_Actinomycetales
d_Bacteria.p_Actinobacteriota.c_Actinobacteria.o_Actinomycetales.f_Actinomycetaceae
d_Bacteria.p_Actinobacteriota.c_Actinobacteria.o_Corynebacteriales
d_Bacteria.p_Actinobacteriota.c_Actinobacteria.o_Corynebacteriales.f_Corynebacteriaceae
d_Bacteria.p_Bacteroidota.c_Bacteroidia.o_Bacteroidales.f_Marinifilaceae
d_Bacteria.p_Bacteroidota.c_Bacteroidia.o_Bacteroidales.f_Porphyromonadaceae
d_Bacteria.p_Bacteroidota.c_Bacteroidia.o_Bacteroidales.f_Rikenellaceae
d_Bacteria.p_Bacteroidota.c_Bacteroidia.o_Bacteroidales.f_Tannerellaceae
d_Bacteria.p_Desulfobacterota
d_Bacteria.p_Desulfobacterota.c_Desulfovibrionia
d_Bacteria.p_Desulfobacterota.c_Desulfovibrionia.o_Desulfovibrionales
d_Bacteria.p_Desulfobacterota.c_Desulfovibrionia.o_Desulfovibrionales.f_Desulfovibrionaceae
d_Bacteria.p_Firmicutes.c_Bacilli.o_Lactobacillales
d_Bacteria.p_Firmicutes.c_Bacilli.o_Lactobacillales.f_Enterococcaceae
d_Bacteria.p_Firmicutes.c_Bacilli.o_Lactobacillales.f_Lactobacillaceae
d_Bacteria.p_Firmicutes.c_Bacilli.o_Lactobacillales.f_Streptococcaceae
d_Bacteria.p_Firmicutes.c_Clostridia.o_Peptostreptococcales_Tissierellales.f_Anaerovoracaceae
d_Bacteria.p_Firmicutes.c_Clostridia.o_Peptostreptococcales_Tissierellales.f_Peptostreptococcales_Tissierell[illegible]
d_Bacteria.p_Proteobacteria.c_Gammaproteobacteria.o_Aeromonadales
d_Bacteria.p_Proteobacteria.c_Gammaproteobacteria.o_Aeromonadales.f_Succinivibrionaceae
d_Bacteria.p_Proteobacteria.c_Gammaproteobacteria.o_Burkholderiales.f_Burkholderiaceae
d_Bacteria.p_Proteobacteria.c_Gammaproteobacteria.o_Burkholderiales.f_Comamonadaceae
d_Bacteria.p_Proteobacteria.c_Gammaproteobacteria.o_Enterobacterales
d_Bacteria.p_Proteobacteria.c_Gammaproteobacteria.o_Enterobacterales.f_Enterobacteriaceae
d_Bacteria.p_Proteobacteria.c_Gammaproteobacteria.o_Enterobacterales.f_Morganellaceae
d_Bacteria.p_Proteobacteria.c_Gammaproteobacteria.o_Oceanospirillales
d_Bacteria.p_Proteobacteria.c_Gammaproteobacteria.o_Oceanospirillales.f_Marinomonadaceae
d_Bacteria.p_Proteobacteria.c_Gammaproteobacteria.o_Pseudomonadales

d_Bacteria.p_Proteobacteria.c_Gammaproteobacteria.o_Pseudomonadales.f_Pseudomonadaceae
d_Bacteria.p_Synergistota
d_Bacteria.p_Synergistota.c_Synergistia
d_Bacteria.p_Synergistota.c_Synergistia.o_Synergistales
d_Bacteria.p_Synergistota.c_Synergistia.o_Synergistales.f_Synergistaceae
d_Bacteria.p_Actinobacteriota.c_Actinobacteria.o_Bifidobacteriales
d_Bacteria.p_Actinobacteriota.c_Actinobacteria.o_Bifidobacteriales.f_Bifidobacteriaceae
d_Bacteria.p_Firmicutes.c_Clostridia.o_Peptococcales
d_Bacteria.p_Firmicutes.c_Clostridia.o_Peptococcales.f_Peptococcaceae
d_Bacteria.p_Deferribacterota
d_Bacteria.p_Deferribacterota.c_Deferribacteres
d_Bacteria.p_Deferribacterota.c_Deferribacteres.o_Deferribacterales
d_Bacteria.p_Deferribacterota.c_Deferribacteres.o_Deferribacterales.f_Deferribacteraceae
d_Bacteria.p_Firmicutes.c_Bacilli.o_Acholeplasmatales
d_Bacteria.p_Firmicutes.c_Bacilli.o_Acholeplasmatales.f_Acholeplasmataceae
d_Bacteria.p_Firmicutes.c_Negativicutes.o_Veillonellales_Selenomonadales.f_Veillonellaceae
d_Bacteria.p_Cyanobacteria
d_Bacteria.p_Cyanobacteria.c_Vampirivibrionia
d_Bacteria.p_Cyanobacteria.c_Vampirivibrionia.o_Gastranaerophilales
d_Bacteria.p_Cyanobacteria.c_Vampirivibrionia.o_Gastranaerophilales.f_Gastranaerophilales
d_Bacteria.p_Actinobacteriota.c_Coriobacteriia.o_Coriobacteriales.f_Eggerthellaceae
d_Bacteria.p_Firmicutes.c_Clostridia.o_Oscillospirales.f__Eubacterium__coprostanoligenes_group
d_Bacteria.p_Firmicutes.c_Bacilli.o_Lactobacillales.f_Vagococcaceae
d_Bacteria.p_Bacteroidota.c_Bacteroidia.o_Bacteroidales.f_Barnesiellaceae
d_Bacteria.p_Actinobacteriota.c_Actinobacteria.o_Micrococcales
d_Bacteria.p_Actinobacteriota.c_Actinobacteria.o_Micrococcales.f_Microbacteriaceae
d_Bacteria.p_Verrucomicrobiota
d_Bacteria.p_Verrucomicrobiota.c_Lentisphaeria
d_Bacteria.p_Verrucomicrobiota.c_Lentisphaeria.o_Victivallales
d_Bacteria.p_Verrucomicrobiota.c_Lentisphaeria.o_Victivallales.f_Victivallaceae
d_Bacteria.p_Verrucomicrobiota.c_Verrucomicrobiae
d_Bacteria.p_Verrucomicrobiota.c_Verrucomicrobiae.o_Verrucomicrobiales
d_Bacteria.p_Verrucomicrobiota.c_Verrucomicrobiae.o_Verrucomicrobiales.f_Akkermansiaceae
d_Bacteria.p_Firmicutes.c_Clostridia.o_Christensenellales
d_Bacteria.p_Firmicutes.c_Clostridia.o_Christensenellales.f_Christensenellaceae

Log of the highest class average	group	LDA (log10)	p-value
2.63	pre-CAD	2.54	0.03
2.74	pre-CAD	2.63	0.02
4.25	pre-CAD	3.70	0.03
4.43	pre-CAD	3.86	0.04
4.43	pre-CAD	3.86	0.04
4.43	pre-CAD	3.87	0.04
4.45	pre-CAD	4.34	0.01
4.45	pre-CAD	4.34	0.01
4.47	pre-CAD	4.36	0.01
4.47	pre-CAD	4.36	0.01
4.74	pre-CAD	4.45	0.02
4.84	pre-CAD	4.57	0.03
5.52			-
3.54			-
4.84			-
3.84			-
3.85			-
3.82			-
3.51			-
3.77			-
3.84			-
3.54			-
4.37			-
3.64			-
3.48			-
2.55			-
2.99			-
3.74			-
3.29			-
2.85			-
2.38			-
3.20			-
2.60			-
4.10			-
4.85			-
5.41			-
3.68			-
4.61			-
1.93			-
1.99			-
3.21			-
1.83			-
2.33			-
3.62			-
4.88			-
1.69			-
2.63			-
4.18			-
1.90			-

2.34	-
4.18	-
4.07	-
3.81	-
2.26	-
3.23	-
2.92	-
2.02	-
2.44	-
1.85	-
2.92	-
4.39	-
3.80	-
2.85	-
2.40	-
4.08	-
2.27	-
2.51	-
2.39	-
3.03	-
1.87	-
2.77	-
2.60	-
4.52	-
3.20	-
1.83	-
2.83	-
1.83	-
1.93	-
3.83	-
2.64	-
3.22	-
1.59	-
3.02	-
3.59	-
2.65	-
1.59	-
2.74	-
1.71	-
3.43	-
2.45	-
2.92	-
3.80	-
2.00	-
3.68	-
3.85	-
0.00	-
2.07	-
3.88	-
2.75	-

	2.34	-
	0.00	-
	2.19	-
	2.86	-
	1.14	-
	3.12	-
	2.29	-
	2.87	-
	3.64	-
	1.61	-
	2.28	-
	1.42	-
	3.78	-
	0.00	-
	1.21	-
	1.93	-
	2.39	-
	1.54	-
	2.47	-
	1.77	-
	0.00	-
	0.00	-
	0.82	-
	2.71	-
	2.11	-
	0.00	-
	0.00	-
	0.00	-
	0.00	-
	0.00	-
	0.00	-
	6.00	-
	4.47	-
	5.68	-
	5.68	-
	5.68	-
	5.52	-
	3.54	-
	5.16	-
	4.10	-
	4.10	-
	4.10	-
	3.84	-
	3.85	-
	5.41	-
	4.87	-
	4.16	-
	5.07	-
	3.77	-
	3.77	-

	3.88	-
	3.88	-
	4.64	-
	4.64	-
	4.57	-
	2.85	-
	3.81	-
	4.48	-
	4.40	-
	4.40	-
	4.92	-
	4.85	-
	4.85	-
	5.41	-
	5.41	-
	5.41	-
	5.41	-
	4.94	-
	4.94	-
	4.66	-
	4.66	-
	3.27	-
	2.26	-
	2.26	-
	3.23	-
	3.23	-
	2.33	-
	3.62	-
	2.63	-
	4.18	-
	3.85	-
	3.85	-
	3.85	-
	3.85	-
	3.44	-
	2.26	-
	3.23	-
	2.92	-
	2.85	-
	2.51	-
	3.93	-
	3.93	-
	1.87	-
	2.99	-
	4.61	-
	4.59	-
	3.20	-
	1.83	-
	1.83	-
	2.83	-

	2.83	-
	1.83	-
	1.83	-
	1.83	-
	1.83	-
	1.93	-
	1.93	-
	2.65	-
	2.65	-
	2.00	-
	2.00	-
	2.00	-
	2.00	-
	3.68	-
	3.68	-
	2.34	-
	3.12	-
	3.12	-
	3.12	-
	3.12	-
	2.97	-
	2.47	-
	1.77	-
	0.00	-
	0.82	-
	0.82	-
	0.00	-
	0.00	-
	0.00	-
	0.00	-
	0.00	-
	0.00	-
	0.00	-
	0.00	-
	0.00	-
