# Supplementary material for: Analysis of Intestinal Microbiota and Metabolic Pathways before and after a 2-Month-Long Hydrolyzed Fish and Rice Starch Hypoallergenic Diet Trial in Pruritic Dogs
Source: Vet Sci. 2023 Jul 21;10(7):478. doi: 10.3390/vetsci10070478 (PMC10384699; doi:10.3390/vetsci10070478)
Supplement: Supplementary file 1 [file vetsci-10-00478-s001.zip › Table S7.pdf]

phylum

	taxon	lfc_(Intercept)	lfc_pre-diet-Al	se_(Intercept)	se_pre-diet-Al	W_(Intercept)
1	Campilobacte	0.3903412	-0.77388873	0.51807644	0.73267073	0.75344325
2	Proteobacteri	0.30422178	-0.60164988	0.35729968	0.50529806	0.85144709
3	Firmicutes	-0.03311255	0.07301877	0.18862584	0.26675722	-0.17554622
4	Fusobacteriot	0.09614859	-0.18550352	0.23973125	0.33903119	0.40106825
5	Actinobacteric	0.24739363	-0.4879936	0.41033756	0.58030494	0.60290272
6	Bacteroidota	0.15289515	-0.29899664	0.3568714	0.50469238	0.42843207

phylum

W_pre-diet-AI	p_(Intercept)	p_pre-diet-AF	q_(Intercept)	q_pre-diet-AF	diff_(Intercept	diff_pre-diet-A
-1.05625719	0.4511836	0.29085073	1	1	FALSE	FALSE
-1.19068315	0.39452104	0.23377799	1	1	FALSE	FALSE
0.27372746	0.86065046	0.78429408	1	1	FALSE	FALSE
-0.54715768	0.68836988	0.58427041	1	1	FALSE	FALSE
-0.84092614	0.5465734	0.40038931	1	1	FALSE	FALSE
-0.59243343	0.66833658	0.55356038	1	1	FALSE	FALSE

class

	taxon	lfc_(Intercept)	lfc_pre-diet-Al	se_(Intercept)	se_pre-diet-Al	W_(Intercept)
1	Campylobacte	0.3903412	-0.77388873	0.52675088	0.74493823	0.74103569
2	Gammaprote	0.30387293	-0.6009522	0.36976846	0.52293157	0.82179246
3	Clostridia	-0.0676714	0.14213646	0.2329713	0.32947118	-0.29047096
4	Fusobacteriia	0.09614859	-0.18550352	0.25794255	0.36478585	0.37275197
5	Coriobacteriia	0.24739363	-0.4879936	0.42123651	0.59571839	0.58730339
6	Negativicutes	0.29970395	-0.59261424	0.42320361	0.59850029	0.7081791
7	Bacilli	0.23129563	-0.45579759	0.30858605	0.43640657	0.74953367
8	Vampirivibrior	0.51844798	-1.03010228	0.35117482	0.4966362	1.47632445
9	Bacteroidia	0.15289515	-0.29899664	0.36935144	0.52234181	0.41395575

class

W_pre-diet-Al	p_(Intercept)	p_pre-diet-AF	q_(Intercept)	q_pre-diet-AF	diff_(Intercept	diff_pre-diet-A
-1.03886295	0.4586718	0.29886848	1	1	FALSE	FALSE
-1.14919854	0.41119502	0.25047412	1	1	FALSE	FALSE
0.43140789	0.77145596	0.66617182	1	1	FALSE	FALSE
-0.50852717	0.70933306	0.61108369	1	1	FALSE	FALSE
-0.81916826	0.55699996	0.41269042	1	1	FALSE	FALSE
-0.99016533	0.47883404	0.32209331	1	1	FALSE	FALSE
-1.04443338	0.45353562	0.29628492	1	1	FALSE	FALSE
-2.07415869	0.13985681	0.03806458	1	0.34258118	FALSE	FALSE
-0.57241567	0.67890651	0.5670404	1	1	FALSE	FALSE

class

ıFR

order

	taxon	lfc_(Intercept)	lfc_pre-diet-Al	se_(Intercept)	se_pre-diet-Al	W_(Intercept)
1	Campylobacte	0.3903412	-0.77388873	0.53352405	0.75451694	0.73162813
2	Burkholderiale	0.31763113	-0.62846858	0.50370318	0.71234387	0.63059186
3	Aeromonadale	0.02491626	-0.04303884	0.65958612	0.93279563	0.03777559
4	Enterobactera	-0.00946161	0.02571688	0.54531787	0.77119593	-0.01735063
5	Peptostreptoc	-0.26807432	0.5429423	0.38901478	0.55014997	-0.68911089
6	Clostridiales	-0.21579128	0.43837623	0.43382355	0.61351915	-0.49741717
7	Peptococcales	0.21042911	-0.41406455	0.37351175	0.52822538	0.56338016
8	Lachnospirale	0.00203367	0.00272633	0.26828645	0.37941433	0.00758021
9	Oscillospirales	0.0084631	-0.01013253	0.42360049	0.59906156	0.01997896
10	Clostridia_UC	0.54923132	-1.09166898	0.40559668	0.57360032	1.3541317
11	Fusobacteriale	0.09614859	-0.18550352	0.2715065	0.38396818	0.35412998
12	Coriobacterial	0.24739363	-0.4879936	0.42967618	0.60765389	0.57576762
13	Acidaminococ	0.38385889	-0.76092412	0.69240512	0.97920871	0.55438483
14	Veillonellales-	0.08813669	-0.16947971	0.54219623	0.76678126	0.16255496
15	Erysipelotricha	0.19990978	-0.39302589	0.30263507	0.42799063	0.66056382
16	Lactobacillales	-0.17746296	0.36171958	0.36518625	0.51645135	-0.48595192
17	Acholeplasma	0.2820294	-0.55726514	0.4805731	0.67963299	0.58686057
18	Gastranaerop	0.51844798	-1.03010228	0.361255	0.51089173	1.43513023
19	Bacteroidales	0.15289515	-0.29899664	0.37894842	0.535914	0.4034722

order

W_pre-diet-Al	p_(Intercept)	p_pre-diet-AF	q_(Intercept)	q_pre-diet-AF	diff_(Intercept	diff_pre-diet-A
-1.02567442	0.46439558	0.30504507	1	1	FALSE	FALSE
-0.8822545	0.52830742	0.3776392	1	1	FALSE	FALSE
-0.04613963	0.96986661	0.96319896	1	1	FALSE	FALSE
0.03334675	0.9861569	0.97339807	1	1	FALSE	FALSE
0.98689872	0.49075349	0.32369229	1	1	FALSE	FALSE
0.71452738	0.6188949	0.47490113	1	1	FALSE	FALSE
-0.78387856	0.57317604	0.43311137	1	1	FALSE	FALSE
0.00718563	0.99395192	0.99426674	1	1	FALSE	FALSE
-0.01691401	0.98406015	0.98650522	1	1	FALSE	FALSE
-1.90318752	0.17569436	0.05701608	1	1	FALSE	FALSE
-0.48312211	0.72324147	0.62900904	1	1	FALSE	FALSE
-0.80307821	0.56477226	0.42192953	1	1	FALSE	FALSE
-0.77708063	0.57931551	0.4371112	1	1	FALSE	FALSE
-0.22102745	0.87086885	0.82507106	1	1	FALSE	FALSE
-0.91830491	0.50889208	0.35845926	1	1	FALSE	FALSE
0.70039431	0.62700126	0.48368109	1	1	FALSE	FALSE
-0.8199501	0.55729735	0.41224456	1	1	FALSE	FALSE
-2.01628296	0.15124999	0.0437704	1	0.83163752	FALSE	FALSE
-0.55791907	0.68660088	0.57689965	1	1	FALSE	FALSE

order

.FR

family

	taxon	lfc_(Intercept)	lfc_pre-diet-AFR	se_(Intercept)	se_pre-diet-Al
1	Campylobacteraceae	0.10444474	-0.202095815	0.51396322	0.72685376
2	Helicobacteraceae	0.51024781	-1.01370196	0.52748457	0.74597583
3	Sutterellaceae	0.31763113	-0.628468583	0.47658339	0.6739907
4	Succinivibrionaceae	0.02491626	-0.043038843	0.63911554	0.90384587
5	Enterobacteriaceae	-0.00765385	0.02210137	0.51950451	0.73469033
6	Peptostreptococcaceae	-0.26995039	0.546694454	0.35347886	0.4998946
7	Anaerovoracaceae	-0.02533397	0.057461602	0.13445597	0.19014946
8	Clostridiaceae	-0.21579128	0.43837623	0.40201705	0.56853796
9	Peptococcaceae	0.21042911	-0.414064549	0.33604443	0.4752386
10	Lachnospiraceae	0.00203367	0.002726332	0.21305505	0.30130534
11	Oscillospiraceae	0.69911829	-1.391442907	0.44668527	0.63170837
12	Butyricicoccaceae	0.37905993	-0.751326201	0.31706199	0.44839336
13	Ruminococcaceae	-0.15275911	0.312311883	0.51636561	0.73025125
14	Clostridia_UCG-014	0.54923132	-1.091668976	0.37138053	0.52521139
15	Fusobacteriaceae	0.09614859	-0.185503518	0.21709587	0.30701993
16	Eggerthellaceae	0.01388143	-0.020969194	0.12607742	0.17830039
17	Coriobacteriaceae	0.25105819	-0.49532272	0.39639658	0.56058942
18	Acidaminococcaceae	0.38385889	-0.760924119	0.67293364	0.95167188
19	Selenomonadaceae	0.01339296	-0.019992244	0.52360283	0.74048623
20	Erysipelatoclostridiacea	0.33247502	-0.65815638	0.34064967	0.48175138
21	Erysipelotrichaceae	0.25022231	-0.493650947	0.32717841	0.46270015
22	Streptococcaceae	-0.17746296	0.361719585	0.32676572	0.46211651
23	Acholeplasmataceae	0.2820294	-0.557265137	0.45206785	0.63932048
24	uncultured_1	0.1710397	-0.335285733	0.26279894	0.37165382
25	Gastranaerophilales	0.51844798	-1.030102284	0.32236627	0.45589476
26	Tannerellaceae	1.2207507	-2.434707735	0.45792084	0.64759786
27	Rikenellaceae	0.4239089	-0.841024133	0.42079823	0.59509856
28	Marinifilaceae	0.14300108	-0.279208493	0.15054854	0.21290779
29	Muribaculaceae	0.21151574	-0.416237815	0.33672434	0.47620013
30	Bacteroidaceae	0.30481044	-0.602827212	0.33715391	0.47680763
31	Prevotellaceae	0.25505969	-0.503325711	0.70646494	0.9990923

family

W_(Intercept)	W_pre-diet-Al	p_(Intercept)	p_pre-diet-AF	q_(Intercept)	q_pre-diet-AF	diff_(Intercept
0.20321443	-0.27804192	0.83896744	0.78098018	1	1	FALSE
0.96732273	-1.35889383	0.33338273	0.17418023	1	1	FALSE
0.66647544	-0.93245884	0.50510726	0.35109945	1	1	FALSE
0.03898553	-0.04761746	0.96890193	0.96202112	1	1	FALSE
-0.01473298	0.03008257	0.98824521	0.9760012	1	1	FALSE
-0.763696	1.09361944	0.44504842	0.27412192	1	1	FALSE
-0.1884183	0.30219177	0.85054875	0.76250588	1	1	FALSE
-0.53677147	0.77105885	0.59142548	0.44067205	1	1	FALSE
0.6261943	-0.87127719	0.53118751	0.38360282	1	1	FALSE
0.00954527	0.0090484	0.99238409	0.99278052	1	1	FALSE
1.565125	-2.20266657	0.1175536	0.02761826	1	0.8009295	FALSE
1.19553889	-1.67559618	0.23187655	0.09381732	1	1	FALSE
-0.29583517	0.4276773	0.76735596	0.66888608	1	1	FALSE
1.47889098	-2.07853257	0.13916946	0.03766033	1	1	FALSE
0.44288541	-0.60420677	0.65784862	0.54570618	1	1	FALSE
0.11010244	-0.11760599	0.91232813	0.90637986	1	1	FALSE
0.63335106	-0.88357486	0.52650442	0.37692575	1	1	FALSE
0.57042607	-0.79956562	0.56838875	0.42396251	1	1	FALSE
0.02557846	-0.02699881	0.97959356	0.97846069	1	1	FALSE
0.97600278	-1.36617435	0.32906309	0.17188425	1	1	FALSE
0.76478857	-1.0668917	0.44439746	0.28602074	1	1	FALSE
-0.54308928	0.78274543	0.58706833	0.43377662	1	1	FALSE
0.62386521	-0.87165226	0.53271611	0.38339811	1	1	FALSE
0.65083864	-0.90214526	0.51515066	0.36697971	1	1	FALSE
1.6082575	-2.25951773	0.10777879	0.0238512	1	0.71553595	FALSE
2.66585533	-3.75959819	0.00767927	0.00017019	0.23805747	0.00527578	FALSE
1.00739232	-1.41325184	0.31374628	0.15758169	1	1	FALSE
0.94986691	-1.3114057	0.34217988	0.18972073	1	1	FALSE
0.62815697	-0.87408169	0.52990111	0.38207377	1	1	FALSE
0.90406912	-1.26429858	0.36595875	0.20612288	1	1	FALSE
0.36103658	-0.50378299	0.7180721	0.61441388	1	1	FALSE

diff_pre-diet-AFR
FALSE
FALSE
FALSE
FALSE
FALSE
FALSE
FALSE
FALSE
FALSE
FALSE
FALSE
FALSE
FALSE
FALSE
FALSE
FALSE
FALSE
FALSE
FALSE
FALSE
FALSE
FALSE
FALSE
FALSE
FALSE
TRUE
FALSE
FALSE
FALSE
FALSE
FALSE

genus

	taxon	lfc_(Intercept)	lfc_pre-diet-Al	se_(Intercept)	se_pre-diet-Al
1	Genus:Campylobacter	0.10444474	-0.20209582	0.52139901	0.73736955
2	Genus:Helicobacter	0.51024781	-1.01370196	0.53473237	0.75622577
3	Genus:Sutterella	0.27110968	-0.5354257	0.47436303	0.67085064
4	Genus:Parasutterella	0.48767761	-0.96856156	0.55650535	0.78701742
5	Genus:Anaerobiospirillum	0.32414711	-0.64150055	0.65906976	0.93206539
6	Genus:Succinivibrio	-0.10892216	0.22463798	0.43666003	0.61753054
7	Genus:Escherichia-Shigella	-0.00756585	0.02192537	0.52473787	0.74209141
8	Genus:Romboutsia	-0.44977169	0.90633706	0.41989479	0.59382091
9	Genus:[Eubacterium]_brachy	-0.02533397	0.0574616	0.16055261	0.22705568
10	Genus:Peptoclostridium	0.20031237	-0.39383107	0.44133643	0.62414397
11	Genus:Terrisporobacter	-0.39567274	0.79813914	0.29189497	0.41280182
12	Genus:Candidatus_Arthromit	-0.09850245	0.20379857	0.19084804	0.26989989
13	Genus:Clostridium_sensu_st	-0.16321468	0.33322304	0.41652887	0.58906077
14	Genus:Peptococcus	0.21042911	-0.41406455	0.34731051	0.49117124
15	Genus:Epulopiscium	-0.22914612	0.46508591	0.22422492	0.31710192
16	Family:Lachnospiraceae	0.33880811	-0.67082255	0.36108731	0.51065456
17	Genus:Tuzzerella	0.22742451	-0.44805535	0.30020898	0.42455961
18	Genus:Tyzzerella	-0.03344468	0.07368303	0.32789236	0.46370983
19	Family:Oscillospiraceae	0.06759229	-0.12839091	0.1923433	0.27201451
20	Genus:Colidextribacter	0.33896942	-0.67114517	0.27305994	0.38616507
21	Genus:Flavonifractor	0.26873564	-0.53067761	0.40571447	0.5737669
22	Genus:Intestinimonas	0.07028306	-0.13377245	0.25950986	0.36700236
23	Genus:Butyricicoccus	0.04744723	-0.08810078	0.30551134	0.43205827
24	Genus:uncultured_1	0.47781671	-0.94883975	0.43636842	0.61711813
25	Genus:Incertae_Sedis	-0.13225062	0.27129491	0.18770751	0.26545851
26	Genus:uncultured_2	0.4034586	-0.80012353	0.30875036	0.43663895
27	Genus:Negativibacillus	0.92732487	-1.84785607	0.40082343	0.56684993
28	Family:Ruminococcaceae	-0.21479702	0.43638771	0.37010644	0.52340955
29	Genus:Fournierella	0.04846767	-0.09014166	0.29405015	0.41584971
30	Genus:Faecalibacterium	-0.34204108	0.69087583	0.66685311	0.94307271
31	Genus:Oscillibacter	0.21027351	-0.41375335	0.24894392	0.35205987
32	Genus:UCG-005	0.45152297	-0.89625227	0.52213759	0.73841406
33	Genus:Clostridia_UCG-014	0.54923132	-1.09166898	0.38160481	0.5396707
34	Genus:Lachnoclostridium	0.23033436	-0.45387506	0.32666612	0.46197566
35	Genus:uncultured_3	0.21419057	-0.42158748	0.31035155	0.43890338
36	Genus:Lachnospiraceae_NK4	0.02532471	-0.04385575	0.40405823	0.57142463
37	Genus:Lachnospiraceae_UC	0.00732232	-0.00785098	0.19511386	0.27593266
38	Genus:[Ruminococcus]_torqu	0.2003659	-0.39393814	0.34666265	0.49025502
39	Genus:Sellimonas	0.1066189	-0.20644413	0.23690611	0.33503583
40	Genus:Blautia	-0.14433515	0.29546397	0.24923643	0.35247354
41	Genus:[Ruminococcus]_gnav	-0.26512273	0.53703914	0.38527346	0.54485895
42	Genus:Roseburia	0.44207938	-0.8773651	0.35747241	0.50554233
43	Genus:[Ruminococcus]_gauv	0.06598454	-0.12517542	0.27266304	0.38560376
44	Genus:Lachnospira	-0.54868192	1.10415751	0.31436691	0.44458195
45	Genus:Fusobacterium	0.09614859	-0.18550352	0.23415668	0.33114755
46	Genus:Slackia	0.01388143	-0.02096919	0.15360419	0.21722913
47	Genus:Collinsella	0.25105819	-0.49532272	0.40599136	0.57415848
48	Genus:Phascolarctobacteriur	0.38385889	-0.76092412	0.67862981	0.95972748
49	Genus:Megamonas	0.01339296	-0.01999224	0.53090363	0.75081111
50	Genus:Candidatus_Stoquefic	0.03231762	-0.05784157	0.24602268	0.34792861
51	Genus:Catenibacterium	-0.45344455	0.91368276	0.34339536	0.48563438
52	Genus:Erysipelatoclostridium	0.60692826	-1.20706285	0.36825096	0.52078551
53	Genus:Erysipelotrichaceae_U	-0.01347252	0.0337387	0.24386106	0.34487161
54	Genus:Turicibacter	-0.33970817	0.68621	0.40618249	0.57442878
55	Genus:Streptococcus	-0.17746296	0.36171958	0.3383409	0.47848629

56	Genus:uncultured_4	0.22795887	-0.44912408	0.30804101	0.43563578
57	Genus:Anaeroplasma	0.2820294	-0.55726514	0.46050415	0.65125121
58	Genus:uncultured_5	0.1710397	-0.33528573	0.27705958	0.39182142
59	Genus:Faecalitalea	-0.06058223	0.12795813	0.42883109	0.60645874
60	Genus:Holdemanella	-0.12671568	0.26022504	0.41161982	0.58211834
61	Genus:Allobaculum	0.55000057	-1.09320747	0.53558084	0.75742568
62	Genus:Gastranaerophilales	0.51844798	-1.03010228	0.33409392	0.47248015
63	Genus:Parabacteroides	1.2207507	-2.43470774	0.46625125	0.65937884
64	Genus:Rikenellaceae_RC9_g	0.4239089	-0.84102413	0.42984867	0.60789782
65	Genus:Odoribacter	0.14300108	-0.27920849	0.17425153	0.24642888
66	Genus:Muribaculaceae	0.21151574	-0.41623781	0.34796841	0.49210164
67	Genus:Bacteroides	0.30481044	-0.60282721	0.34838411	0.49268954
68	Genus:Prevotellaceae_Ga6A	-0.1858007	0.37839506	0.63921071	0.90398045
69	Genus:Prevotella	-0.75498125	1.51675618	0.78657528	1.11238543
70	Genus:Alloprevotella	0.5612114	-1.11562914	0.75760151	1.07141032

genus

W_(Intercept)	W_pre-diet-Al	p_(Intercept)	p_pre-diet-AF	q_(Intercept)	q_pre-diet-AF	diff_(Intercept
0.20031634	-0.2740767	0.84123318	0.78402568	1	1	FALSE
0.95421158	-1.3404753	0.33997656	0.18009087	1	1	FALSE
0.57152363	-0.79812952	0.56764475	0.42479533	1	1	FALSE
0.87632151	-1.2306736	0.38085527	0.21844497	1	1	FALSE
0.49182519	-0.68825702	0.62284293	0.49129094	1	1	FALSE
-0.24944385	0.36376822	0.80301747	0.7160311	1	1	FALSE
-0.01441835	0.02954538	0.98849622	0.97642962	1	1	FALSE
-1.0711533	1.52628013	0.28410051	0.12694012	1	1	FALSE
-0.15779231	0.25307274	0.87462046	0.80021201	1	1	FALSE
0.45387681	-0.63099396	0.6499175	0.52804448	1	1	FALSE
-1.35553122	1.93346809	0.17524837	0.05317856	1	1	FALSE
-0.51613027	0.7550895	0.60576343	0.45019528	1	1	FALSE
-0.39184484	0.56568533	0.69517286	0.57160771	1	1	FALSE
0.60588177	-0.84301465	0.54459327	0.39922025	1	1	FALSE
-1.02194761	1.46667642	0.3068057	0.1424641	1	1	FALSE
0.9382997	-1.31365232	0.34809041	0.18896323	1	1	FALSE
0.75755399	-1.05534144	0.44871803	0.29126919	1	1	FALSE
-0.10199896	0.15889901	0.9187575	0.87374844	1	1	FALSE
0.35141484	-0.47200025	0.72527715	0.63692661	1	1	FALSE
1.24137368	-1.73797484	0.21446774	0.08221525	1	1	FALSE
0.66237628	-0.92490105	0.5077301	0.35501738	1	1	FALSE
0.27083001	-0.36450024	0.78652178	0.7154845	1	1	FALSE
0.15530431	-0.20390949	0.87658142	0.83842423	1	1	FALSE
1.09498463	-1.53753342	0.27352337	0.12416274	1	1	FALSE
-0.70455687	1.02198609	0.48108604	0.30678748	1	1	FALSE
1.30674698	-1.8324603	0.19129866	0.06688287	1	1	FALSE
2.31354955	-3.2598682	0.02069244	0.00111464	1	0.07691016	FALSE
-0.58036553	0.83374044	0.56166815	0.40442727	1	1	FALSE
0.16482789	-0.216765	0.86907945	0.82839149	1	1	FALSE
-0.51291818	0.7325796	0.60800856	0.46381488	1	1	FALSE
0.84466216	-1.17523577	0.39829949	0.2399004	1	1	FALSE
0.8647586	-1.21375299	0.3871713	0.22484206	1	1	FALSE
1.43926728	-2.02284277	0.15007481	0.04308936	1	1	FALSE
0.70510637	-0.9824653	0.48074405	0.32587067	1	1	FALSE
0.69015466	-0.96054735	0.49009693	0.33677981	1	1	FALSE
0.06267589	-0.07674809	0.9500246	0.93882395	1	1	FALSE
0.03752847	-0.02845252	0.97006364	0.97730123	1	1	FALSE
0.57798526	-0.80353718	0.56327407	0.42166432	1	1	FALSE
0.45004707	-0.61618523	0.6526765	0.53777228	1	1	FALSE
-0.57910935	0.83825856	0.56251539	0.4018855	1	1	FALSE
-0.68814171	0.98564801	0.49136355	0.32430587	1	1	FALSE
1.23668114	-1.73549286	0.21620548	0.08265355	1	1	FALSE
0.24200033	-0.32462188	0.80877991	0.74546727	1	1	FALSE
-1.7453552	2.48358602	0.08092306	0.01300669	1	0.88445504	FALSE
0.41061649	-0.56018388	0.68135377	0.57535402	1	1	FALSE
0.09037144	-0.09653031	0.92799205	0.92309941	1	1	FALSE
0.6183831	-0.86269338	0.53632283	0.38830607	1	1	FALSE
0.56563813	-0.79285436	0.5716398	0.42786268	1	1	FALSE
0.02522672	-0.02662753	0.97987413	0.97875682	1	1	FALSE
0.13136032	-0.1662455	0.89549028	0.86796375	1	1	FALSE
-1.3204737	1.88142108	0.18667691	0.05991466	1	1	FALSE
1.6481376	-2.3177735	0.09932444	0.02046163	1	1	FALSE
-0.05524669	0.09782975	0.95594193	0.92206749	1	1	FALSE
-0.83634371	1.19459544	0.40296157	0.23224513	1	1	FALSE
-0.52450933	0.75596646	0.59992433	0.44966931	1	1	FALSE

0.74002766	-1.03096232	0.45928321	0.30255849	1	1	FALSE
0.61243619	-0.85568384	0.54024921	0.39217268	1	1	FALSE
0.61733905	-0.85571058	0.53701111	0.39215789	1	1	FALSE
-0.14127295	0.21099231	0.88765432	0.83289327	1	1	FALSE
-0.30784641	0.44703116	0.75819921	0.65485256	1	1	FALSE
1.02692354	-1.44331978	0.30445647	0.14893041	1	1	FALSE
1.5518031	-2.18020225	0.12070934	0.02924247	1	1	FALSE
2.61822507	-3.69242627	0.00883885	0.00022212	0.61871939	0.01554873	FALSE
0.98618172	-1.38349589	0.32404395	0.16651287	1	1	FALSE
0.82065897	-1.13301857	0.41184055	0.25720646	1	1	FALSE
0.60785904	-0.84583708	0.54328097	0.39764364	1	1	FALSE
0.87492635	-1.22354376	0.38161398	0.22112439	1	1	FALSE
-0.29067206	0.41858766	0.77130214	0.67551751	1	1	FALSE
-0.95983344	1.36351676	0.33713905	0.17271971	1	1	FALSE
0.74077388	-1.0412716	0.45883056	0.29774951	1	1	FALSE

diff_pre-diet-AFR
FALSE
FALSE
FALSE
FALSE
FALSE
FALSE
FALSE
FALSE
FALSE
FALSE
FALSE
FALSE
FALSE
FALSE
FALSE
FALSE
FALSE
FALSE
FALSE
FALSE
FALSE
FALSE
FALSE
FALSE
FALSE
FALSE
FALSE
FALSE
FALSE
FALSE
FALSE
FALSE
FALSE
FALSE
FALSE
FALSE
FALSE
FALSE
FALSE
FALSE
FALSE
FALSE
FALSE
FALSE
FALSE
FALSE
FALSE
FALSE
FALSE
FALSE
FALSE
FALSE
FALSE
FALSE
FALSE

genus
FALSE
FALSE
FALSE
FALSE
FALSE
FALSE
FALSE
TRUE
FALSE
FALSE
FALSE
FALSE
FALSE
FALSE
FALSE
