# Supplementary material for: Analysis of Intestinal Microbiota and Metabolic Pathways before and after a 2-Month-Long Hydrolyzed Fish and Rice Starch Hypoallergenic Diet Trial in Pruritic Dogs
Source: Vet Sci. 2023 Jul 21;10(7):478. doi: 10.3390/vetsci10070478 (PMC10384699; doi:10.3390/vetsci10070478)
Supplement: Supplementary file 1 [file vetsci-10-00478-s001.zip › Table S8.pdf]

phylum

	taxon	lfc_(Intercept)	lfc_pre-diet-D	se_(Intercept)	se_pre-diet-D	W_(Intercept)
1	Bacteroidota	0.04778	-0.0954802	0.26233189	0.37099332	0.1821357
2	Campilobacte	-0.20511632	0.41031245	0.68652726	0.97089616	-0.29877375
3	Desulfobacter	0.34917732	-0.69827483	0.50441812	0.71335495	0.69223785
4	Proteobacteria	-0.04289453	0.08586887	0.63538964	0.89857665	-0.06750902
5	Deferribacterc	-0.08279186	0.16566354	0.42576429	0.60212163	-0.1944547
6	Firmicutes	-0.0827604	0.16560062	0.34678467	0.49042758	-0.2386507
7	Actinobacteric	0.19947441	-0.39886902	0.51061171	0.722114	0.39065774
8	Fusobacteriota	0.10310105	-0.20612229	0.80365498	1.13653977	0.12829019

phylum

W_pre-diet-D	p_(Intercept)	p_pre-diet-D	q_(Intercept)	q_pre-diet-D	diff_(Intercept	diff_pre-diet-D
-0.25736366	0.85547623	0.79689805	1	1	FALSE	FALSE
0.42261208	0.76511268	0.67257831	1	1	FALSE	FALSE
-0.97886028	0.48878797	0.32764902	1	1	FALSE	FALSE
0.09556098	0.94617649	0.92386926	1	1	FALSE	FALSE
0.27513301	0.84581987	0.78321405	1	1	FALSE	FALSE
0.33766579	0.81137645	0.73561506	1	1	FALSE	FALSE
-0.55236295	0.69605024	0.58069971	1	1	FALSE	FALSE
-0.18135951	0.89791933	0.8560854	1	1	FALSE	FALSE

class

	taxon	lfc_(Intercept)	lfc_pre-diet-D	se_(Intercept)	se_pre-diet-D
1	Bacteroidia	0.04778	-0.0954802	0.30588145	0.43258169
2	Campylobacte	-0.20511632	0.41031245	0.7043189	0.99605734
3	Desulfovibrior	0.34917732	-0.69827483	0.52837778	0.74723903
4	Gammaprote(	-0.04289453	0.08586887	0.65457271	0.92570561
5	Deferribactere	-0.08279186	0.16566354	0.45389499	0.64190446
6	Clostridia	-0.04889799	0.09787578	0.43423893	0.61410658
7	Negativicutes	-0.02821856	0.05651693	0.73690916	1.04214693
8	Coriobacteriia	0.15135592	-0.30263203	0.52246341	0.73887484
9	Fusobacteriia	0.10310105	-0.20612229	0.81890584	1.15810774
10	Bacilli	-0.05301648	0.10611277	0.57110267	0.80766114

class

W_(Intercept)	W_pre-diet-D	p_(Intercept)	p_pre-diet-D	q_(Intercept)	q_pre-diet-D	diff_(Intercept
0.15620432	-0.22072177	0.87587197	0.82530908	1	1	FALSE
-0.29122649	0.41193657	0.77087811	0.68038591	1	1	FALSE
0.66084784	-0.93447318	0.5087099	0.35005987	1	1	FALSE
-0.06553058	0.09276045	0.94775156	0.92609387	1	1	FALSE
-0.18240312	0.2580813	0.85526637	0.79634416	1	1	FALSE
-0.11260618	0.15937914	0.91034279	0.87337017	1	1	FALSE
-0.03829313	0.05423125	0.96945397	0.95675092	1	1	FALSE
0.28969668	-0.40958496	0.77204829	0.68211043	1	1	FALSE
0.12590098	-0.17798197	0.8998103	0.85873714	1	1	FALSE
-0.09283179	0.13138279	0.9260372	0.8954725	1	1	FALSE

diff_pre-diet-D
FALSE
FALSE
FALSE
FALSE
FALSE
FALSE
FALSE
FALSE
FALSE
FALSE

order

	taxon	lfc_(Intercept)	lfc_pre-diet-D	se_(Intercept)	se_pre-diet-D	W_(Intercept)
1	Bacteroidales	0.04778	-0.0954802	0.32939562	0.46583575	0.14505355
2	Campylobacte	-0.20511632	0.41031245	0.71484483	1.01094325	-0.28693824
3	Desulfovibrion	0.34917732	-0.69827483	0.54232932	0.76696949	0.64384738
4	Aeromonadale	-0.37822114	0.75652208	0.87823316	1.24200924	-0.43066142
5	Burkholderiale	0.01581636	-0.03155291	0.93745939	1.32576778	0.01687152
6	Enterobactera	0.1776562	-0.3552326	0.87322477	1.23492632	0.20344842
7	Deferribactera	-0.08279186	0.16566354	0.47006242	0.66476865	-0.17612951
8	Acidaminococ	0.01528328	-0.03048675	1.02034843	1.44299059	0.01497849
9	Coriobacterial	0.15135592	-0.30263203	0.53656876	0.75882281	0.28208112
10	Peptococcales	0.30566747	-0.61125512	0.49381061	0.69835366	0.61899737
11	Peptostreptoc	0.15409849	-0.30811716	0.41137051	0.58176575	0.3745978
12	Fusobacteriale	0.10310105	-0.20612229	0.82797632	1.17093534	0.12452174
13	Erysipelotricha	-0.08269802	0.16547584	0.57064742	0.80701732	-0.14491964
14	Acholeplasma	0.1729701	-0.3458604	0.74288755	1.05060164	0.23283484
15	Lactobacillales	-0.30117984	0.60243948	0.55088388	0.77906745	-0.5467211
16	Veillonellales-	-0.18749222	0.37506426	0.74820811	1.05812606	-0.25058833
17	Clostridiales	0.23445791	-0.46883601	0.87222003	1.23350539	0.26880592
18	Lachnospirale	-0.02422813	0.04853606	0.42888197	0.60653069	-0.05649136
19	Clostridia_UC	-0.10061116	0.20130212	0.75526299	1.06810316	-0.13321341
20	Oscillospirales	-0.18466422	0.36940824	0.79509836	1.12443888	-0.2322533

W_pre-diet-D	p_(Intercept)	p_pre-diet-D	q_(Intercept)	q_pre-diet-D	diff_(Intercept	diff_pre-diet-D
-0.20496537	0.88466859	0.83759918	1	1	FALSE	FALSE
0.4058709	0.77415961	0.68483746	1	1	FALSE	FALSE
-0.91043365	0.5196744	0.36259386	1	1	FALSE	FALSE
0.60911148	0.66671458	0.54245055	1	1	FALSE	FALSE
-0.02379973	0.98653912	0.98101236	1	1	FALSE	FALSE
-0.28765489	0.83878456	0.77361092	1	1	FALSE	FALSE
0.2492048	0.8601922	0.80320237	1	1	FALSE	FALSE
-0.02112748	0.98804934	0.98314397	1	1	FALSE	FALSE
-0.39881777	0.77788131	0.69002748	1	1	FALSE	FALSE
-0.87528019	0.53591809	0.38142147	1	1	FALSE	FALSE
-0.5296241	0.70795961	0.59637258	1	1	FALSE	FALSE
-0.17603217	0.90090219	0.86026867	1	1	FALSE	FALSE
0.20504621	0.88477432	0.83753602	1	1	FALSE	FALSE
-0.32920222	0.81588966	0.74200284	1	1	FALSE	FALSE
0.77328283	0.58457036	0.43935501	1	1	FALSE	FALSE
0.35446084	0.8021324	0.72299354	1	1	FALSE	FALSE
-0.38008428	0.78807903	0.70388285	1	1	FALSE	FALSE
0.08002243	0.95495038	0.93621942	1	1	FALSE	FALSE
0.18846693	0.89402461	0.85051064	1	1	FALSE	FALSE
0.32852674	0.81634128	0.74251343	1	1	FALSE	FALSE

order

family

	taxon	lfc_(Intercept)	lfc_pre-diet-D	se_(Intercept)	se_pre-diet-D	W_(Intercept)
1	Bacteroidacea	0.17137143	-0.34266306	0.34719574	0.49100893	0.49358737
2	Prevotellacea	-0.30473244	0.60954469	1.19203481	1.68579179	-0.25564056
3	Tannerellacea	0.47615961	-0.95223941	0.68904907	0.97446254	0.69103875
4	Muribaculacea	-0.00903978	0.01815937	0.62934745	0.8900317	-0.01436374
5	Campylobacte	0.24005097	-0.48002212	0.84086325	1.18916021	0.28548158
6	Helicobactera	-0.41523109	0.83054199	0.71998744	1.018216	-0.57671991
7	Desulfovibrion	0.34917732	-0.69827483	0.55078537	0.77892813	0.63396259
8	Succinivibrion	-0.37822114	0.75652208	0.88347999	1.24942939	-0.42810379
9	Sutterellaceae	0.01581636	-0.03155291	0.94237653	1.33272167	0.01678348
10	Enterobacteria	0.1776562	-0.3552326	0.87850152	1.24238877	0.2022264
11	Deferribactera	-0.08279186	0.16566354	0.47979381	0.67853092	-0.17255717
12	Acidaminococ	0.01528328	-0.03048675	1.02486797	1.44938218	0.01491244
13	Coriobacteria	0.15135592	-0.30263203	0.54511417	0.77090785	0.27765912
14	Peptococcace	0.30566747	-0.61125512	0.50308284	0.71146657	0.60758874
15	Anaerovoraca	0.09481379	-0.18954778	0.45605259	0.64495575	0.20790101
16	Peptostreptoc	0.16529989	-0.33051997	0.42308995	0.59833954	0.3906968
17	Fusobacteriac	0.10310105	-0.20612229	0.83353956	1.17880296	0.12369065
18	Erysipelatoclo	-0.27768842	0.55545665	0.68271777	0.96550873	-0.40673969
19	Erysipelotricha	0.02484879	-0.04961777	0.60279062	0.85247466	0.04122292
20	uncultured	-0.18196097	0.36400176	0.58352589	0.82523023	-0.31183016
21	Acholeplasma	0.1729701	-0.3458604	0.74908299	1.05936333	0.23090913
22	Streptococcac	-0.3114745	0.6230288	0.5404569	0.76432147	-0.576317
23	Selenomonad	-0.18749222	0.37506426	0.75435986	1.06682595	-0.2485448
24	Clostridiaceae	0.23445791	-0.46883601	0.87750282	1.24097639	0.26718764
25	Lachnospirace	-0.02422813	0.04853606	0.43952607	0.62158372	-0.0551233
26	Clostridia_UC	-0.10061116	0.20130212	0.76135774	1.07672244	-0.13214702
27	Oscillospirace	0.35187378	-0.70366775	0.80386118	1.13683139	0.43772953
28	Butyricicoccac	0.07718654	-0.15429327	0.58777693	0.83124211	0.13131944
29	Ruminococca	-0.40132067	0.80272115	0.88272327	1.24835922	-0.45463928

family

W_pre-diet-D	p_(Intercept)	p_pre-diet-D	q_(Intercept)	q_pre-diet-D	diff_(Intercept	diff_pre-diet-D
-0.69787542	0.62159762	0.48525511	1	1	FALSE	FALSE
0.36157768	0.7982284	0.71766764	1	1	FALSE	FALSE
-0.97719448	0.48954119	0.32847288	1	1	FALSE	FALSE
0.02040306	0.98853979	0.98372184	1	1	FALSE	FALSE
-0.4036648	0.77527522	0.68645922	1	1	FALSE	FALSE
0.8156835	0.56412868	0.41468118	1	1	FALSE	FALSE
-0.89645603	0.52610524	0.37000925	1	1	FALSE	FALSE
0.60549407	0.66857555	0.54485077	1	1	FALSE	FALSE
-0.02367555	0.98660935	0.98111141	1	1	FALSE	FALSE
-0.28592708	0.83973973	0.77493397	1	1	FALSE	FALSE
0.24415032	0.86299952	0.8071144	1	1	FALSE	FALSE
-0.02103431	0.98810204	0.98321829	1	1	FALSE	FALSE
-0.39256576	0.78127405	0.69464023	1	1	FALSE	FALSE
-0.85914806	0.54346027	0.39025883	1	1	FALSE	FALSE
-0.29389269	0.83530626	0.76883991	1	1	FALSE	FALSE
-0.55239533	0.69602137	0.58067753	1	1	FALSE	FALSE
-0.17485729	0.90156022	0.86119177	1	1	FALSE	FALSE
0.57529945	0.68419918	0.56508879	1	1	FALSE	FALSE
-0.05820439	0.96711818	0.95358582	1	1	FALSE	FALSE
0.44109116	0.7551696	0.65914701	1	1	FALSE	FALSE
-0.32647949	0.8173854	0.74406161	1	1	FALSE	FALSE
0.81513973	0.56440094	0.41499233	1	1	FALSE	FALSE
0.35157024	0.80371291	0.72516059	1	1	FALSE	FALSE
-0.37779608	0.78932469	0.70558209	1	1	FALSE	FALSE
0.07808451	0.95604024	0.93776083	1	1	FALSE	FALSE
0.18695823	0.894868	0.85169338	1	1	FALSE	FALSE
-0.61897283	0.66158236	0.53593426	1	1	FALSE	FALSE
-0.18561773	0.89552261	0.85274455	1	1	FALSE	FALSE
0.64302097	0.64936877	0.52021049	1	1	FALSE	FALSE

family

genus

	taxon	lfc_(Intercept)	lfc_pre-diet-D	se_(Intercept)	se_pre-diet-D	W_(Intercept)
1	Genus:Bacter	0.17137143	-0.34266306	0.31261267	0.44210107	0.54819095
2	Genus:Prevot	0.07918588	-0.15829196	0.925011	1.3081631	0.08560534
3	Genus:Parapr	0.51959183	-1.03910384	0.46019638	0.65081597	1.12906543
4	Genus:Allopre	0.01583012	-0.03158043	1.20328583	1.70170313	0.01315574
5	Genus:Prevot	-0.93536962	1.87081904	1.22884957	1.73785573	-0.76117504
6	Genus:Paraba	0.47615961	-0.95223941	0.67228745	0.95075803	0.70826788
7	Genus:Muriba	-0.00903978	0.01815937	0.61095008	0.8640139	-0.01479627
8	Genus:Campy	0.24005097	-0.48002212	0.82718366	1.16981435	0.29020274
9	Genus:Helicol	-0.41523109	0.83054199	0.70396286	0.99555383	-0.58984801
10	Genus:Anaer	-0.13065087	0.26138155	0.89915736	1.27160053	-0.14530368
11	Genus:Succin	-0.22147902	0.44303786	0.60272794	0.85238603	-0.36746102
12	Genus:Suttere	0.0265671	-0.05305439	0.91539246	1.29456043	0.02902263
13	Genus:Parasu	-0.33697347	0.67402674	0.89740904	1.26912804	-0.37549596
14	Genus:Escher	0.17758748	-0.35509514	0.86360117	1.22131648	0.20563598
15	Genus:Mucisp	-0.08279186	0.16566354	0.45539422	0.64402469	-0.18180262
16	Genus:Phasco	0.01528328	-0.03048675	1.01367458	1.43355233	0.01507711
17	Genus:Collins	0.15135592	-0.30263203	0.52376641	0.74071756	0.28897599
18	Genus:Peptoc	0.30566747	-0.61125512	0.47986887	0.67863707	0.63698123
19	Genus:[Eubac	0.051533	-0.10298619	0.3749672	0.53028369	0.13743335
20	Genus:Rombo	0.05881644	-0.11755308	0.62534467	0.88437091	0.09405444
21	Genus:Peptoc	0.16476753	-0.32945525	0.37148298	0.52535627	0.44353992
22	Genus:Terrisp	-0.12080314	0.24168609	0.37099822	0.52467071	-0.32561649
23	Genus:Fusoba	0.10310105	-0.20612229	0.81973776	1.15928426	0.12577321
24	Genus:Erysipe	-0.26598577	0.53205134	0.62205083	0.87971273	-0.4275949
25	Genus:Erysipe	-0.10867539	0.21743059	0.48414134	0.68467924	-0.22447038
26	Genus:Candic	0.06152286	-0.12296591	0.4416303	0.62455956	0.13930851
27	Genus:Cateni	-0.57722286	1.15452553	0.71575937	1.01223661	-0.8064482
28	Genus:Faecal	0.36637436	-0.73266891	0.68539477	0.96929458	0.53454502
29	Genus:uncultu	-0.18196097	0.36400176	0.56363487	0.79710007	-0.32283484
30	Genus:Holder	-0.13429599	0.26867179	0.67991981	0.96155181	-0.1975174
31	Genus:Allobac	-0.11215098	0.22438176	0.79803316	1.12858932	-0.14053423
32	Genus:uncultu	0.04413384	-0.08818786	0.55055914	0.7786082	0.08016185
33	Genus:Anaer	0.1729701	-0.3458604	0.73369416	1.03760024	0.23575232
34	Genus:Turicib	-0.29012119	0.58032218	0.561337	0.79385039	-0.5168396
35	Genus:Strepto	-0.3114745	0.6230288	0.51891758	0.73386028	-0.60023886
36	Genus:Megan	-0.18749222	0.37506426	0.73908091	1.04521825	-0.25368295
37	Genus:Clostri	0.3426045	-0.6851292	0.80102836	1.13282516	0.42770584
38	Genus:Sarcina	-0.10577639	0.21163259	0.63672934	0.90047127	-0.16612457
39	Family:Lachno	0.11093739	-0.22179497	0.76629928	1.08371083	0.14477032
40	Genus:Anaer	0.15426751	-0.30845521	0.50475934	0.7138375	0.30562586
41	Genus:Tuzzer	0.03784466	-0.07560952	0.60603924	0.85706891	0.0624459
42	Genus:Clostri	-0.10061116	0.20130212	0.74622209	1.0553174	-0.13482737
43	Genus:Oscillib	0.39971425	-0.79934868	0.51731829	0.73159855	0.77266598
44	Family:Oscillo	0.37529177	-0.75050373	0.45260556	0.64008092	0.82918065
45	Genus:Flavon	0.26149546	-0.52291112	0.52117174	0.73704814	0.50174528
46	Genus:uncultu	0.0567556	-0.1134314	0.41273494	0.58369535	0.13751102
47	Genus:Intestir	-0.06968209	0.139444	0.39659798	0.56087424	-0.17569957
48	Genus:Colide:	0.32406602	-0.64805223	0.56596429	0.80039438	0.57259092
49	Genus:UCG-0	0.19731847	-0.39455713	0.77020349	1.08923222	0.25619005
50	Genus:Butyric	0.05211308	-0.10414636	0.56205651	0.79486794	0.09271858
51	Genus:uncultu	0.13106051	-0.26204122	0.71921132	1.0171184	0.1822281
52	Genus:uncultu	0.14371904	-0.28735828	0.58759355	0.83098277	0.24458921
53	Family:Rumin	-0.04974502	0.09956984	0.6303394	0.89143453	-0.07891783
54	Genus:Anaer	-0.72328253	1.44664486	0.57433939	0.81223856	-1.25932947
55	Genus:Faecal	-0.61239097	1.22486176	0.9846826	1.39255149	-0.62191713

56	Genus:Negati	0.53194878	-1.06381775	0.73603886	1.04091614	0.72271833
57	Genus:Tyzzer	-0.35106934	0.70221848	0.52543027	0.74307062	-0.6681559
58	Genus:Lachno	-0.42205044	0.84418069	0.53821794	0.7611551	-0.78416272
59	Genus:unculti	0.11096439	-0.22184897	0.49567698	0.70099311	0.22386432
60	Genus:GCA-9	0.1927289	-0.385378	0.39406463	0.55729155	0.48907943
61	Genus:[Rumir	-0.01418295	0.02844571	0.55632953	0.78676877	-0.02549379
62	Genus:Sellimo	0.04393963	-0.08779945	0.32624687	0.46138275	0.13468215
63	Genus:Blautia	-0.18290302	0.36588585	0.46648282	0.65970634	-0.39208951
64	Genus:[Rumir	0.1483421	-0.2966044	0.51192608	0.7239728	0.28977251
65	Genus:[Rumir	0.10845873	-0.21683765	0.53082305	0.75069716	0.20432181
66	Genus:Roseb	0.08834754	-0.17661528	0.63400986	0.89662535	0.13934727
67	Genus:Lachno	0.03106349	-0.06204718	0.48118658	0.68050059	0.06455602
68	Genus:Lachno	0.23086522	-0.46165064	0.38427016	0.54344007	0.60078885
69	Genus:Lachno	-0.09544466	0.19096913	0.62359569	0.88189748	-0.15305536

genus

W_pre-diet-D	p_(Intercept)	p_pre-diet-D	q_(Intercept)	q_pre-diet-D	diff_(Intercept	diff_pre-diet-D
-0.77507855	0.5835608	0.43829324	1	1	FALSE	FALSE
-0.12100323	0.93178015	0.90368848	1	1	FALSE	FALSE
-1.59661701	0.25887023	0.1103511	1	1	FALSE	FALSE
-0.01855813	0.98950354	0.9851936	1	1	FALSE	FALSE
1.07650998	0.44655253	0.28169924	1	1	FALSE	FALSE
-1.0015581	0.47877892	0.31655706	1	1	FALSE	FALSE
0.02101746	0.98819471	0.98323173	1	1	FALSE	FALSE
-0.41034043	0.77166114	0.68155624	1	1	FALSE	FALSE
0.83425122	0.55529255	0.40413943	1	1	FALSE	FALSE
0.20555319	0.88447111	0.83713994	1	1	FALSE	FALSE
0.51976199	0.71327516	0.60322948	1	1	FALSE	FALSE
-0.04098255	0.97684654	0.96730981	1	1	FALSE	FALSE
0.53109436	0.70729165	0.59535339	1	1	FALSE	FALSE
-0.29074785	0.83707527	0.77124417	1	1	FALSE	FALSE
0.25723166	0.85573763	0.79699995	1	1	FALSE	FALSE
-0.02126658	0.98797067	0.98303301	1	1	FALSE	FALSE
-0.40856602	0.77259975	0.68285817	1	1	FALSE	FALSE
-0.90070989	0.52413707	0.36774259	1	1	FALSE	FALSE
-0.19420961	0.89068827	0.84601176	1	1	FALSE	FALSE
-0.13292282	0.92506591	0.89425442	1	1	FALSE	FALSE
-0.62710825	0.65737525	0.53058828	1	1	FALSE	FALSE
0.46064337	0.74471453	0.64505449	1	1	FALSE	FALSE
-0.17780134	0.89991145	0.858879	1	1	FALSE	FALSE
0.60480123	0.66894608	0.54531107	1	1	FALSE	FALSE
0.31756562	0.82239131	0.75081446	1	1	FALSE	FALSE
-0.1968842	0.88920636	0.84391816	1	1	FALSE	FALSE
1.14056883	0.41998446	0.2540494	1	1	FALSE	FALSE
-0.75587848	0.59296451	0.44972206	1	1	FALSE	FALSE
0.45665754	0.74682032	0.64791722	1	1	FALSE	FALSE
0.27941479	0.84342267	0.77992653	1	1	FALSE	FALSE
0.19881613	0.88823791	0.84240658	1	1	FALSE	FALSE
-0.11326347	0.93610854	0.90982168	1	1	FALSE	FALSE
-0.33332722	0.81362487	0.7388873	1	1	FALSE	FALSE
0.7310221	0.60526814	0.46476565	1	1	FALSE	FALSE
0.84897469	0.54834706	0.39589538	1	1	FALSE	FALSE
0.35883822	0.79974051	0.71971612	1	1	FALSE	FALSE
-0.60479695	0.6688653	0.54531392	1	1	FALSE	FALSE
0.23502426	0.86805892	0.81418991	1	1	FALSE	FALSE
-0.2046625	0.88489222	0.83783582	1	1	FALSE	FALSE
-0.43210844	0.75988952	0.6656626	1	1	FALSE	FALSE
-0.08821872	0.95020775	0.92970284	1	1	FALSE	FALSE
0.19075031	0.89274837	0.84872122	1	1	FALSE	FALSE
-1.09260562	0.43972009	0.27456699	1	1	FALSE	FALSE
-1.17251384	0.40700219	0.24099082	1	1	FALSE	FALSE
-0.70946671	0.61584671	0.47803491	1	1	FALSE	FALSE
-0.19433322	0.89062688	0.84591497	1	1	FALSE	FALSE
0.24861901	0.86052997	0.8036555	1	1	FALSE	FALSE
-0.80966614	0.56692171	0.41813208	1	1	FALSE	FALSE
-0.36223417	0.79780409	0.71717705	1	1	FALSE	FALSE
-0.13102347	0.92612714	0.89575674	1	1	FALSE	FALSE
-0.25763099	0.85540372	0.79669171	1	1	FALSE	FALSE
-0.34580534	0.80677452	0.72948901	1	1	FALSE	FALSE
0.11169619	0.93709798	0.9110643	1	1	FALSE	FALSE
1.78105907	0.20791135	0.0749028	1	1	FALSE	FALSE
0.87958095	0.53399636	0.37908636	1	1	FALSE	FALSE

			genus			
-1.02200139	0.46985295	0.30678024	1	1	FALSE	FALSE
0.94502254	0.50403408	0.34464737	1	1	FALSE	FALSE
1.1090784	0.43294464	0.26739636	1	1	FALSE	FALSE
-0.31647811	0.82286288	0.75163964	1	1	FALSE	FALSE
-0.69151955	0.62478547	0.4892391	1	1	FALSE	FALSE
0.0361551	0.9796611	0.97115869	1	1	FALSE	FALSE
-0.19029635	0.89286318	0.84907691	1	1	FALSE	FALSE
0.55461928	0.69499208	0.5791551	1	1	FALSE	FALSE
-0.40968998	0.77199028	0.68203338	1	1	FALSE	FALSE
-0.28884837	0.83810202	0.77269742	1	1	FALSE	FALSE
-0.19697779	0.88917574	0.84384492	1	1	FALSE	FALSE
-0.09117873	0.9485275	0.92735058	1	1	FALSE	FALSE
-0.84949688	0.54798063	0.39560486	1	1	FALSE	FALSE
0.21654346	0.87835462	0.82856415	1	1	FALSE	FALSE
