# Supplementary material for: Analysis of Intestinal Microbiota and Metabolic Pathways before and after a 2-Month-Long Hydrolyzed Fish and Rice Starch Hypoallergenic Diet Trial in Pruritic Dogs
Source: Vet Sci. 2023 Jul 21;10(7):478. doi: 10.3390/vetsci10070478 (PMC10384699; doi:10.3390/vetsci10070478)
Supplement: Supplementary file 1 [file vetsci-10-00478-s001.zip › Table S9.pdf]

phylum

	taxon	lfc_(Intercept)	lfc_pre-diet-C/	se_(Intercept)	se_pre-diet-C/	W_(Intercept)
1	Bacteroidota	-0.25107393	0.56393274	0.28322094	0.41286192	-0.88649493
2	Actinobacteric	1.02748442	-2.15300374	0.8258819	1.20391941	1.24410574
3	Desulfobacter	-0.44059816	0.96667173	0.89700604	1.30759977	-0.4911875
4	Proteobacteria	-0.08404393	0.20899399	0.45859138	0.66850607	-0.18326539
5	Campilobacte	0.29058285	-0.5870879	0.77464006	1.12922223	0.37511983
6	Firmicutes	0.28417081	-0.57346233	0.43303626	0.6312534	0.65622868
7	Fusobacteriota	0.00333183	0.02332051	0.32913674	0.47979512	0.01012293

W_pre-diet-CA	p_(Intercept)	p_pre-diet-CA	q_(Intercept)	q_pre-diet-CA	diff_(Intercept	diff_pre-diet-C
1.36591125	0.37535089	0.17196682	1	1	FALSE	FALSE
-1.78832878	0.21346065	0.07372298	1	0.51606085	FALSE	FALSE
0.73927187	0.62329384	0.45974193	1	1	FALSE	FALSE
0.31262842	0.85458979	0.75456299	1	1	FALSE	FALSE
-0.51990466	0.70757135	0.60313003	1	1	FALSE	FALSE
-0.90845028	0.511677	0.36364037	1	1	FALSE	FALSE
0.04860514	0.99192321	0.96123397	1	1	FALSE	FALSE

:AD

class

	taxon	lfc_(Intercept)	lfc_pre-diet-C/	se_(Intercept)	se_pre-diet-C/	W_(Intercept)
1	Bacteroidia	-0.25107393	0.56393274	0.29057122	0.4235767	-0.86407018
2	Coriobacteriia	1.31644719	-2.76704963	0.7700593	1.12254468	1.70954002
3	Actinobacteria	-0.20935363	0.47527711	0.57348571	0.8359919	-0.36505466
4	Desulfovibrion	-0.44059816	0.96667173	0.89935387	1.31102228	-0.48990522
5	Gammaprotec	-0.08355521	0.20795547	0.46304338	0.67499591	-0.18044791
6	Campylobacte	0.29058285	-0.5870879	0.77735754	1.13318361	0.37380848
7	Clostridia	0.29654272	-0.59975264	0.40311787	0.58764023	0.73562287
8	Bacilli	0.07166026	-0.12187741	0.71185481	1.03769778	0.10066696
9	Fusobacteriia	0.00333183	0.02332051	0.33548253	0.48904562	0.00993145
10	Negativicutes	0.73702854	-1.53578499	0.87719643	1.27872254	0.84020923

W_pre-diet-C/	p_(Intercept)	p_pre-diet-CA	q_(Intercept)	q_pre-diet-CA	diff_(Intercept	diff_pre-diet-C
1.3313592	0.38754934	0.18307085	1	1	FALSE	FALSE
-2.46497949	0.08735097	0.01370211	0.87350966	0.13702109	FALSE	FALSE
0.5685188	0.71507061	0.56968274	1	1	FALSE	FALSE
0.73734195	0.62420097	0.46091443	1	1	FALSE	FALSE
0.30808404	0.85680094	0.75801839	1	1	FALSE	FALSE
-0.51808718	0.70854681	0.60439744	1	1	FALSE	FALSE
-1.02061195	0.46196024	0.30743832	1	1	FALSE	FALSE
-0.11744981	0.91981484	0.90650361	1	1	FALSE	FALSE
0.04768576	0.99207598	0.96196668	1	1	FALSE	FALSE
-1.20103067	0.40079108	0.2297393	1	1	FALSE	FALSE

class

:AD

order

	taxon	lfc_(Intercept)	lfc_pre-diet-CAD	se_(Intercept)	se_pre-diet-C.	W_(Intercept)
1	Bacteroidales	-0.25107393	0.56393274	0.316063166	0.46073728	-0.79437895
2	Coriobacterial	1.31644719	-2.767049625	0.78003565	1.13708759	1.68767567
3	Corynebacteri	-0.23536254	0.530546051	0.559678801	0.81586504	-0.42053146
4	Desulfovibrion	-0.44059816	0.966671729	0.907910604	1.32349576	-0.48528804
5	Pseudomonac	-0.17017987	0.392032868	0.507972382	0.74049063	-0.33501796
6	Burkholderiale	0.43268679	-0.889058788	0.965377432	1.40726734	0.44820479
7	Campylobacte	0.29058285	-0.5870879	0.787241412	1.1475917	0.36911529
8	Enterobactera	-1.14892714	2.471870813	0.939163657	1.36905453	-1.22335136
9	Aeromonadale	-0.29684247	0.661190888	0.981705948	1.43107004	-0.30237411
10	Clostridiales	0.09205506	-0.165216356	0.857931419	1.25063921	0.10729886
11	Erysipelotricha	0.13186643	-0.249815517	0.713018389	1.03939398	0.18494113
12	Acholeplasma	0.41593249	-0.853455897	0.65843352	0.95982355	0.63170006
13	Lactobacillales	-0.51091309	1.116090972	0.62520339	0.91138272	-0.81719502
14	Fusobacteriale	0.00333183	0.023320511	0.357788788	0.5215623	0.00931228
15	Veillonellales-	0.19025485	-0.3738909	1.041684754	1.51850342	0.18264148
16	Acidaminococ	1.21981745	-2.561711434	0.927607563	1.35220877	1.31501456
17	Peptostreptoc	0.38324422	-0.783993331	0.533866834	0.77823796	0.71786483
18	Clostridia_UC	0.71816816	-1.495706692	0.831824718	1.21258248	0.86336477
19	Oscillospirales	0.57144992	-1.183930434	0.705218679	1.02802405	0.81031592
20	Peptococcales	0.35327078	-0.720299758	0.428946661	0.62529184	0.82357741
21	Lachnospirale	0.53186392	-1.099810174	0.638648918	0.93098278	0.83279545

W_pre-diet-C/	p_(Intercept)	p_pre-diet-CA	q_(Intercept)	q_pre-diet-CA	diff_(Intercept	diff_pre-diet-C
1.22397897	0.42697485	0.22096016	1	1	FALSE	FALSE
-2.43345337	0.09147351	0.01495556	1	0.31406679	FALSE	FALSE
0.65028654	0.67409725	0.51550715	1	1	FALSE	FALSE
0.73039276	0.62747204	0.46515014	1	1	FALSE	FALSE
0.52942313	0.73761154	0.59651196	1	1	FALSE	FALSE
-0.63176254	0.6540054	0.52754206	1	1	FALSE	FALSE
-0.51158256	0.71204179	0.60894319	1	1	FALSE	FALSE
1.80553131	0.22119702	0.07099158	1	1	FALSE	FALSE
0.46202553	0.76236689	0.64406302	1	1	FALSE	FALSE
-0.13210553	0.91455189	0.89490082	1	1	FALSE	FALSE
-0.24034728	0.85327521	0.81006104	1	1	FALSE	FALSE
-0.88918	0.52758289	0.37390635	1	1	FALSE	FALSE
1.22461283	0.41381699	0.22072113	1	1	FALSE	FALSE
0.0447128	0.99256999	0.96433623	1	1	FALSE	FALSE
-0.24622328	0.85507934	0.80550939	1	1	FALSE	FALSE
-1.89446444	0.18850501	0.05816339	1	1	FALSE	FALSE
-1.00739539	0.47284063	0.3137448	1	1	FALSE	FALSE
-1.23348862	0.38793694	0.21739352	1	1	FALSE	FALSE
-1.15165636	0.41775863	0.24946231	1	1	FALSE	FALSE
-1.15194173	0.41017972	0.24934502	1	1	FALSE	FALSE
-1.1813432	0.4049601	0.23746641	1	1	FALSE	FALSE

order

:AD

family

	taxon	lfc_(Intercept)	lfc_pre-diet-C/	se_(Intercept)	se_pre-diet-C/	W_(Intercept)
1	Muribaculacea	0.11563226	-0.21531792	0.80698817	1.1763773	0.14328867
2	Porphyromona	-0.30208165	0.67232416	0.63971767	0.93254074	-0.47221089
3	Tannerellacea	1.08097584	-2.26667301	0.85196295	1.24193875	1.26880616
4	Rikenellaceae	-0.00627142	0.04372741	0.49367855	0.71965397	-0.01270344
5	Bacteroidacea	-0.22745105	0.51373413	0.45088659	0.6572745	-0.50445291
6	Prevotellacea	0.71985804	-1.49929768	1.16369514	1.69636259	0.61859675
7	Coriobacteriac	1.35115081	-2.84079482	0.80432228	1.17249113	1.67986246
8	Eggerthellace	0.3412521	-0.69476007	0.55643946	0.81114293	0.61327805
9	Corynebacteri	-0.23536254	0.53054605	0.58820534	0.85744927	-0.4001367
10	Desulfovibrion	-0.44059816	0.96667173	0.92576824	1.34952752	-0.47592706
11	Pseudomonac	-0.17017987	0.39203287	0.53924123	0.78607242	-0.31559135
12	Comamonada	-0.17593599	0.40426462	0.57245529	0.83448981	-0.30733577
13	Sutterellaceae	0.44650275	-0.91841769	0.97931794	1.42758895	0.45593237
14	Campylobacte	0.8238054	-1.72018582	0.92513488	1.34860424	0.89047059
15	Helicobactera	0.36190886	-0.73865569	0.75819284	1.1052465	0.47733089
16	Enterobacteria	-1.15436284	2.48342168	0.9501677	1.38509553	-1.21490432
17	Morganellacea	-0.42593252	0.93550726	0.55734783	0.8124671	-0.76421312
18	Succinivibrion	-0.29684247	0.66119089	0.99824433	1.45517866	-0.29736454
19	Clostridiaceae	0.09205506	-0.16521636	0.87680755	1.27815567	0.1049889
20	Erysipelatoclo	0.07984389	-0.13926763	0.84521201	1.23209764	0.09446611
21	Acholeplasma	0.41593249	-0.8534559	0.68284688	0.99541183	0.60911531
22	Erysipelotricha	0.13690685	-0.26052641	0.7120178	1.03793539	0.1922801
23	uncultured_1	0.42299843	-0.86847102	0.60190099	0.87741392	0.70277079
24	Lactobacillace	-0.24149224	0.54357166	0.58069341	0.84649884	-0.41586874
25	Streptococcac	-0.28886926	0.64424783	0.517885	0.75494063	-0.5577865
26	Enterococcac	-0.18149955	0.41608719	0.39736331	0.57925159	-0.45675971
27	Fusobacteriac	0.00333183	0.02332051	0.40094636	0.58447473	0.00830991
28	Veillonellacea	-0.11977745	0.28492774	0.4464098	0.65074851	-0.26831278
29	Selenomonad	0.07573395	-0.130534	1.1083379	1.61566625	0.0683311
30	Acidaminococ	1.21981745	-2.56171143	0.94509309	1.37769809	1.29068497
31	Peptostreptoc	0.39724843	-0.81375228	0.55728608	0.81237708	0.71282676
32	Clostridia_UC	0.71816816	-1.49570669	0.85127993	1.24094309	0.84363337
33	Butyricicoccac	0.27946237	-0.56345689	0.61714392	0.89963413	0.45283176
34	Oscillospirace	0.38164753	-0.78060036	0.77350776	1.12757164	0.49339845
35	Ruminococca	0.72951449	-1.51981764	0.80094578	1.16756907	0.91081632
36	[Eubacterium]	0.00339609	0.02318396	0.51919959	0.75685696	0.006541
37	Peptococcace	0.35327078	-0.72029976	0.46555387	0.67865555	0.75881827
38	Lachnospirace	0.53186392	-1.09981017	0.66379033	0.96763237	0.80125288

family

W_pre-diet-C/	p_(Intercept)	p_pre-diet-CA	q_(Intercept)	q_pre-diet-CA	diff_(Intercept	diff_pre-diet-C
-0.18303474	0.8860622	0.85477076	1	1	FALSE	FALSE
0.72095955	0.63677627	0.4709344	1	1	FALSE	FALSE
-1.82510854	0.20451021	0.06798465	1	1	FALSE	FALSE
0.06076171	0.98986439	0.95154899	1	1	FALSE	FALSE
0.78161276	0.61394314	0.43444219	1	1	FALSE	FALSE
-0.8838309	0.53618204	0.3767875	1	1	FALSE	FALSE
-2.42287105	0.09298408	0.01539839	1	0.58513888	FALSE	FALSE
-0.85651992	0.53969251	0.39171026	1	1	FALSE	FALSE
0.6187492	0.68905584	0.53608159	1	1	FALSE	FALSE
0.71630383	0.63412634	0.47380376	1	1	FALSE	FALSE
0.4987236	0.7523127	0.61797412	1	1	FALSE	FALSE
0.48444524	0.75858781	0.62806991	1	1	FALSE	FALSE
-0.64333483	0.64843861	0.52000686	1	1	FALSE	FALSE
-1.27553048	0.37321326	0.20212154	1	1	FALSE	FALSE
-0.6683176	0.63312652	0.50393088	1	1	FALSE	FALSE
1.79296057	0.22440259	0.07297923	1	1	FALSE	FALSE
1.15144018	0.44474025	0.24955119	1	1	FALSE	FALSE
0.45437093	0.76618821	0.64956187	1	1	FALSE	FALSE
-0.12926153	0.91638462	0.89715071	1	1	FALSE	FALSE
-0.11303295	0.9247389	0.91000443	1	1	FALSE	FALSE
-0.85738975	0.54244801	0.39122952	1	1	FALSE	FALSE
-0.25100446	0.84752281	0.80181066	1	1	FALSE	FALSE
-0.98980766	0.4821986	0.32226814	1	1	FALSE	FALSE
0.64214105	0.67750605	0.5207816	1	1	FALSE	FALSE
0.85337548	0.57699018	0.39345111	1	1	FALSE	FALSE
0.7183186	0.64784377	0.47256087	1	1	FALSE	FALSE
0.03989995	0.99336973	0.96817289	1	1	FALSE	FALSE
0.43784616	0.78845857	0.66149781	1	1	FALSE	FALSE
-0.08079268	0.94552207	0.93560683	1	1	FALSE	FALSE
-1.85941423	0.19681294	0.06296845	1	1	FALSE	FALSE
-1.0016928	0.47595296	0.31649198	1	1	FALSE	FALSE
-1.20529838	0.39887431	0.22808813	1	1	FALSE	FALSE
-0.62631782	0.65066989	0.5311065	1	1	FALSE	FALSE
-0.69228449	0.62173108	0.48875869	1	1	FALSE	FALSE
-1.30169398	0.36239216	0.19302102	1	1	FALSE	FALSE
0.03063189	0.99478107	0.97556311	1	1	FALSE	FALSE
-1.0613628	0.44796128	0.28852505	1	1	FALSE	FALSE
-1.13659919	0.42298526	0.25570588	1	1	FALSE	FALSE

family

:AD

genus

	taxon	lfc_(Intercept)	lfc_pre-diet-C/	se_(Intercept)	se_pre-diet-C/	W_(Intercept)
1	Genus:Muriba	0.11563226	-0.21531792	0.78967794	1.15114352	0.14642965
2	Genus:Porphy	-0.30208165	0.67232416	0.61773783	0.9004999	-0.48901271
3	Genus:Paraba	1.08220433	-2.26928355	0.83485045	1.2169932	1.29628526
4	Genus:Rikene	-0.00627142	0.04372741	0.4648439	0.6776206	-0.01349145
5	Genus:Bacter	-0.22745105	0.51373413	0.41911818	0.61096449	-0.54268954
6	Genus:Prevot	-0.28082534	0.62715449	0.90180374	1.31459356	-0.31140405
7	Genus:Parapr	-0.03543614	0.10570244	0.8272026	1.20584464	-0.04283852
8	Genus:Allopre	0.90118821	-1.88462429	1.3072884	1.90568394	0.68935684
9	Genus:Prevot	0.36048514	-0.73563027	1.25823006	1.83416973	0.28650177
10	Genus:Collins	1.35115081	-2.84079482	0.78695341	1.14717187	1.71693875
11	Genus:Slackia	0.22248962	-0.44238979	0.4012505	0.58491809	0.55449057
12	Genus:Coryne	-0.23297894	0.5254809	0.56157605	0.81863074	-0.41486624
13	Genus:Bilophi	-0.2615086	0.58610642	0.91675918	1.33639466	-0.28525332
14	Genus:Pseud	-0.17017987	0.39203287	0.51297411	0.74778185	-0.33175138
15	Genus:Comar	-0.17547621	0.40328758	0.45821249	0.66795374	-0.38295815
16	Family:Comar	-0.16915413	0.38985317	0.50002912	0.72891143	-0.33828855
17	Genus:Parasu	0.48855196	-1.00777226	0.90307744	1.31645028	0.54098567
18	Genus:Sutter	0.39665113	-0.812483	0.95516931	1.39238657	0.41526787
19	Genus:Campy	0.8238054	-1.72018582	0.91007465	1.32665038	0.90520639
20	Genus:Helicol	0.36190886	-0.73865569	0.73974166	1.07834951	0.48923683
21	Genus:Escher	-0.72827639	1.57798798	0.93615076	1.36466251	-0.77794777
22	Family:Entero	-0.50205159	1.09726028	0.6319656	0.92124026	-0.79442867
23	Genus:Proteu	-0.42593252	0.93550726	0.53197551	0.7754809	-0.80066191
24	Genus:Anaer	0.09793153	-0.17770386	0.97734966	1.42471972	0.10020112
25	Genus:Succin	-0.14521361	0.33897957	0.6471929	0.94343766	-0.22437454
26	Genus:Clostri	-0.1476818	0.34422447	0.89129283	1.29927141	-0.16569392
27	Genus:Erysip	0.69733657	-1.45143957	0.78506818	1.1444237	0.88824969
28	Genus:Erysip	0.24990501	-0.50064749	0.72217854	1.05274709	0.34604324
29	Genus:Candic	0.02089963	-0.01401108	0.55572761	0.81010523	0.03760769
30	Genus:Cateni	-0.42252293	0.92826188	0.65044858	0.9481836	-0.64958698
31	Genus:Anaer	0.41593249	-0.8534559	0.66229993	0.96545976	0.62801228
32	Genus:Holder	-0.12226187	0.29020711	0.33428469	0.48729948	-0.36574175
33	Genus:unculti	0.08456163	-0.14929283	0.48175483	0.70227232	0.17552836
34	Genus:unculti	0.42299843	-0.86847102	0.57848607	0.84328111	0.73121628
35	Genus:Faecal	-0.20960907	0.47581992	0.79398114	1.15741646	-0.26399754
36	Genus:Holder	-0.2178723	0.49337928	0.91170084	1.32902094	-0.23897345
37	Genus:Alloba	0.93256414	-1.95129814	0.95524718	1.39250008	0.97625427
38	Genus:Lactob	-0.24149224	0.54357166	0.55638671	0.81106604	-0.43403668
39	Genus:Strept	-0.28886926	0.64424783	0.49047549	0.71498475	-0.58895758
40	Genus:Turicib	0.64654547	-1.34350847	0.76838869	1.12010938	0.84143022
41	Genus:Entero	-0.18149955	0.41608719	0.36091403	0.52611809	-0.50288859
42	Genus:Fusoba	0.00333183	0.02332051	0.3648552	0.53186328	0.00913192
43	Genus:Allison	-0.11977745	0.28492774	0.41429825	0.6039383	-0.28910924
44	Genus:Megan	0.07573395	-0.130534	1.09579845	1.59738701	0.06911303
45	Genus:Phasc	1.398907	-2.94227674	0.93788668	1.36719303	1.49155227
46	Genus:Terrisp	-0.2425198	0.54575522	0.31836247	0.46408906	-0.76177258
47	Genus:Rombo	0.07572893	-0.13052334	0.85910256	1.25234643	0.08814889
48	Genus:Clostri	0.03316861	-0.04008264	0.55708503	0.812084	0.05953957
49	Genus:Peptoc	0.25163466	-0.504323	0.87902256	1.28138456	0.28626644
50	Genus:Clostri	0.71816816	-1.49570669	0.83488853	1.21704872	0.86019646
51	Genus:Butyric	0.2654759	-0.53373564	0.52579009	0.76646419	0.50490852
52	Genus:unculti	0.13806474	-0.26298694	0.6651826	0.96966194	0.20755916
53	Genus:UCG-0	0.24197623	-0.48379885	0.85577564	1.24749664	0.28275663
54	Family:Oscillo	0.00739839	0.01467907	0.51908709	0.75669296	0.01425269
55	Genus:Oscillib	0.32482705	-0.65985684	0.65966246	0.96161502	0.49241403

56	Genus:Colide:	0.21104707	-0.41807439	0.66363151	0.96740085	0.31801847
57	Genus:Flavon	0.04452692	-0.06421906	0.55491109	0.80891497	0.08024154
58	Genus:Intestir	0.03393467	-0.04171052	0.46037919	0.67111223	0.07371025
59	Family:Rumin	0.21994584	-0.43698427	0.68486657	0.998356	0.32115138
60	Genus:Faecal	0.3609673	-0.73665487	1.00348941	1.46282463	0.35971212
61	Genus:Fourni	0.13785622	-0.26254382	0.45443442	0.66244632	0.30335778
62	Genus:Harryfl	0.18914879	-0.37154052	0.39359725	0.57376166	0.48056429
63	Genus:Negati	0.80008001	-1.66976937	0.85270402	1.24301903	0.93828573
64	Genus:uncultu	0.47211611	-0.97284609	0.59095479	0.86145723	0.79890394
65	Genus:[Eubac	0.00339609	0.02318396	0.49186335	0.71700788	0.00690453
66	Genus:Phoce	-0.17876936	0.41028553	0.43751212	0.63777803	-0.40860435
67	Genus:Peptoc	0.35327078	-0.72029976	0.4348583	0.63390945	0.81238137
68	Family:Lachno	0.67007775	-1.39351456	0.64536344	0.94077079	1.03829518
69	Genus:Tuzzer	0.5098327	-1.05299383	0.42484519	0.61931296	1.20004348
70	Genus:uncultu	0.67061317	-1.39465233	0.57670995	0.840692	1.16282572
71	Genus:Lachno	0.06004815	-0.09720168	0.59023585	0.86040922	0.10173586
72	Genus:[Rumir	0.65891325	-1.36979001	0.62848155	0.91616142	1.04842099
73	Genus:Lachno	0.95498036	-1.99893262	0.60608138	0.88350784	1.57566359
74	Genus:[Rumir	-0.31173998	0.6928481	0.76647001	1.11731244	-0.40672169
75	Genus:[Rumir	0.37606952	-0.76874708	0.37178733	0.54196851	1.01151784
76	Genus:Tyzzer	0.12783817	-0.24125547	0.52074146	0.7591046	0.24549259
77	Genus:Roseb	0.70760973	-1.47327002	0.70213594	1.02353022	1.00779591
78	Genus:Blautia	0.5009673	-1.03415486	0.57733562	0.84160406	0.86772283
79	Genus:Lachno	0.30891974	-0.6260538	0.60118054	0.87636371	0.51385519
80	Genus:Sellimo	-0.13223682	0.31140389	0.43892353	0.6398355	-0.30127531
81	Genus:Lachno	0.17688147	-0.34547248	0.42886554	0.62517358	0.41244039

W_pre-diet-C/	p_(Intercept)	p_pre-diet-CA	q_(Intercept)	q_pre-diet-CA	diff_(Intercept	diff_pre-diet-C
-0.18704698	0.88358222	0.8516238	1	1	FALSE	FALSE
0.74661214	0.6248327	0.45529772	1	1	FALSE	FALSE
-1.86466412	0.19487723	0.0622285	1	1	FALSE	FALSE
0.06453081	0.98923571	0.94854758	1	1	FALSE	FALSE
0.8408576	0.58734358	0.40042771	1	1	FALSE	FALSE
0.47707102	0.75549347	0.63331155	1	1	FALSE	FALSE
0.08765842	0.96583025	0.93014817	1	1	FALSE	FALSE
-0.98894903	0.49059873	0.32268808	1	1	FALSE	FALSE
-0.4010699	0.77449384	0.68836866	1	1	FALSE	FALSE
-2.47634631	0.08599036	0.01327348	1	1	FALSE	FALSE
-0.75632777	0.57924316	0.4494527	1	1	FALSE	FALSE
0.64190223	0.67823983	0.52093666	1	1	FALSE	FALSE
0.43857286	0.77545007	0.66097107	1	1	FALSE	FALSE
0.52426101	0.740077	0.60009701	1	1	FALSE	FALSE
0.60376574	0.70175079	0.5459994	1	1	FALSE	FALSE
0.53484299	0.73514575	0.59275844	1	1	FALSE	FALSE
-0.76552246	0.58851746	0.4439605	1	1	FALSE	FALSE
-0.58351826	0.67794582	0.55954447	1	1	FALSE	FALSE
-1.29663839	0.36535606	0.19475564	1	1	FALSE	FALSE
-0.68498727	0.62467404	0.49335195	1	1	FALSE	FALSE
1.15632104	0.43659981	0.24754986	1	1	FALSE	FALSE
1.19106853	0.42694592	0.23362668	1	1	FALSE	FALSE
1.20635758	0.4233274	0.22767964	1	1	FALSE	FALSE
-0.124729	0.92018465	0.9007381	1	1	FALSE	FALSE
0.35930256	0.82246588	0.71936876	1	1	FALSE	FALSE
0.26493654	0.86839783	0.79105835	1	1	FALSE	FALSE
-1.26827115	0.37440645	0.20470113	1	1	FALSE	FALSE
-0.47556293	0.72931022	0.63438579	1	1	FALSE	FALSE
-0.01729538	0.97000047	0.98620097	1	1	FALSE	FALSE
0.9789896	0.51595904	0.32758512	1	1	FALSE	FALSE
-0.88398909	0.5299959	0.3767021	1	1	FALSE	FALSE
0.5955416	0.71455779	0.5514815	1	1	FALSE	FALSE
-0.21258538	0.86066449	0.83165037	1	1	FALSE	FALSE
-1.02987131	0.46464706	0.30307042	1	1	FALSE	FALSE
0.4111052	0.79178181	0.6809954	1	1	FALSE	FALSE
0.37123515	0.81112617	0.7104624	1	1	FALSE	FALSE
-1.40129122	0.32893847	0.161127	1	1	FALSE	FALSE
0.67019408	0.66426181	0.50273408	1	1	FALSE	FALSE
0.90106513	0.55588973	0.36755369	1	1	FALSE	FALSE
-1.199444	0.40010696	0.23035535	1	1	FALSE	FALSE
0.79086273	0.6150426	0.4290241	1	1	FALSE	FALSE
0.04384682	0.99271388	0.96502651	1	1	FALSE	FALSE
0.47178286	0.77249778	0.63708179	1	1	FALSE	FALSE
-0.0817172	0.94489965	0.9348716	1	1	FALSE	FALSE
-2.15205657	0.13581656	0.0313929	1	1	FALSE	FALSE
1.17597088	0.44619575	0.23960651	1	1	FALSE	FALSE
-0.10422303	0.92975834	0.91699236	1	1	FALSE	FALSE
-0.04935775	0.95252235	0.9606342	1	1	FALSE	FALSE
-0.39357662	0.77467406	0.69389365	1	1	FALSE	FALSE
-1.22896205	0.38968076	0.21908603	1	1	FALSE	FALSE
-0.69636084	0.61362309	0.48620288	1	1	FALSE	FALSE
-0.27121508	0.83557319	0.78622562	1	1	FALSE	FALSE
-0.38781575	0.7773634	0.69815239	1	1	FALSE	FALSE
0.01939898	0.98862838	0.98452282	1	1	FALSE	FALSE
-0.68619647	0.62242668	0.49258922	1	1	FALSE	FALSE

-0.43216252	0.75047093	0.6656233	1	1	FALSE	FALSE
-0.07938914	0.93604515	0.93672311	1	1	FALSE	FALSE
-0.06215133	0.94124094	0.95044232	1	1	FALSE	FALSE
-0.43770386	0.74809567	0.66160097	1	1	FALSE	FALSE
-0.50358386	0.71906243	0.61455384	1	1	FALSE	FALSE
-0.39632467	0.76161721	0.69186553	1	1	FALSE	FALSE
-0.64755202	0.6308262	0.51727474	1	1	FALSE	FALSE
-1.34331763	0.34809759	0.17916915	1	1	FALSE	FALSE
-1.12930283	0.42434611	0.25877011	1	1	FALSE	FALSE
0.03233432	0.99449102	0.97420544	1	1	FALSE	FALSE
0.64330458	0.68283004	0.52002648	1	1	FALSE	FALSE
-1.13628177	0.41657283	0.25583866	1	1	FALSE	FALSE
-1.48124769	0.29913265	0.13854058	1	1	FALSE	FALSE
-1.70026126	0.23012245	0.08908179	1	1	FALSE	FALSE
-1.65893375	0.24490022	0.09712914	1	1	FALSE	FALSE
-0.11297145	0.91896634	0.91005319	1	1	FALSE	FALSE
-1.49514046	0.29444469	0.13487779	1	1	FALSE	FALSE
-2.26249562	0.11510335	0.0236668	1	1	FALSE	FALSE
0.62010237	0.68421241	0.53519039	1	1	FALSE	FALSE
-1.41843495	0.31176865	0.15606382	1	1	FALSE	FALSE
-0.31781584	0.80607505	0.75062464	1	1	FALSE	FALSE
-1.43940061	0.31355245	0.15003705	1	1	FALSE	FALSE
-1.22879025	0.38554609	0.21915046	1	1	FALSE	FALSE
-0.71437669	0.60735324	0.47499428	1	1	FALSE	FALSE
0.48669368	0.76320457	0.62647543	1	1	FALSE	FALSE
-0.55260249	0.68001666	0.58053563	1	1	FALSE	FALSE

genus

:AD
